# Efficient fdCas9 Synthetic Endonuclease with Improved Specificity for Precise Genome Engineering

**DOI:** 10.1371/journal.pone.0133373

**Published:** 2015-07-30

**Authors:** Mustapha Aouida, Ayman Eid, Zahir Ali, Thomas Cradick, Ciaran Lee, Harshavardhan Deshmukh, Ahmed Atef, Dina AbuSamra, Samah Zeineb Gadhoum, Jasmeen Merzaban, Gang Bao, Magdy Mahfouz

**Affiliations:** 1 Laboratory for Genome Engineering, Division of Biological Sciences & Center for Desert Agriculture, 4700 King Abdullah University of Science and Technology, Thuwal, 23955–6900, Kingdom of Saudi Arabia; 2 Department of Biomedical Engineering, Georgia Institute of Technology and Emory University, Atlanta, GA, 30332, United States of America; 3 Laboratory of Cell Signaling and Migration, Division of Biological and Environmental Sciences and Engineering, King Abdullah University of Science and Technology, Thuwal, 23955, Saudi Arabia; McGill University, CANADA

## Abstract

The Cas9 endonuclease is used for genome editing applications in diverse eukaryotic species. A high frequency of off-target activity has been reported in many cell types, limiting its applications to genome engineering, especially in genomic medicine. Here, we generated a synthetic chimeric protein between the catalytic domain of the FokI endonuclease and the catalytically inactive Cas9 protein (fdCas9). A pair of guide RNAs (gRNAs) that bind to sense and antisense strands with a defined spacer sequence range can be used to form a catalytically active dimeric fdCas9 protein and generate double-strand breaks (DSBs) within the spacer sequence. Our data demonstrate an improved catalytic activity of the fdCas9 endonuclease, with a spacer range of 15–39 nucleotides, on surrogate reporters and genomic targets. Furthermore, we observed no detectable fdCas9 activity at known Cas9 off-target sites. Taken together, our data suggest that the fdCas9 endonuclease variant is a superior platform for genome editing applications in eukaryotic systems including mammalian cells.

## Introduction

The development of precise and highly efficient genome editing tools is transforming biological research and expediting biotechnological applications ranging from superior crop production to genomic medicine uses [1]. Genome editing tools allow precise alteration of DNA sequences on a single nucleotide level. Such control permits functional characterization of genes and their variants and linking a particular genotype to a particular phenotype. Several genome-engineering approaches have been developed including zinc finger nucleases (ZFNs) and transcription activator-like effector nucleases (TALENs) [2–7]. ZFNs are used in a variety of organisms and cell types but are difficult to design or select, and suffer from a high failure rate, rendering them impractical for targeted genome modification [6]. TALENs can be easily engineered to bind almost any user-defined sequence and have proven efficient in genome editing applications across eukaryotic species [2, 8, 9]. Both ZFNs and TALENs require protein engineering and depend on protein DNA recognition and binding. Several repeat assembly protocols have been developed for TALEN engineering [7, 10–14]. However, the requirement of engineering two TALEN proteins with simultaneous expression in single cells for each individual target complicates their applications, especially in genomic medicine. Moreover, TALEN off-target binding and cleavage has been detected, as have effects of TALEN monomer binding [10].

Clustered regularly interspaced short palindromic repeats (CRISPRs) are involved in adaptive immunity in bacteria and archaea against invading foreign DNA from phages and conjugative plasmids [15]. Recently, a type II CRISPR/Cas genome engineering platform was developed for genome editing applications [16–23]. This genome editing system is composed of the Cas9 endonuclease and two RNA molecules, i.e., a CRISPR RNA targeting molecule and a trans-activating CRISPR RNA processing molecule [21, 23–25]. These two RNA molecules can be combined into a single-guide RNA (gRNA) molecule capable of efficiently directing the Cas9 endonuclease to its genomic target [18, 26, 27]. The Cas9 endonuclease possesses two nuclease domains, the HNH and RuvC domains. The HNH domain cleaves the complementary strand and the RuvC domain cleaves the non-complementary strand [26]. The gRNA-guided Cas9 system is used with high efficiency for targeted genome modification across eukaryotic species [28]. A major drawback of this genome editing system is the frequent off-targeting activity of the Cas9 endonuclease [29–34]. Activity at off-target sites complicates the analysis of the intended modification and can produce unwanted effects, limiting the applications of this technology, especially in genomic medicine, where specificity is an essential characteristic of an editing reagent. Two gRNA molecules are required to ensure the proximity of the Cas9 nickases and to cleave the sense and antisense strand simultaneously, thereby generating DSBs [35]. SSB repair is not always effective and can result in deleterious effects in the target gene or other loci. It was recently reported that SSBs introduce unwanted insertions and/or deletions (indels) and point mutations [29, 36]. Thus, a dependable Cas9 platform that is highly specific and efficient is desired to ensure the broad applicability of this technology and to realize its potential in genomic medicine applications. Accordingly, we generated a chimeric protein referred to as fdCas9 by fusing the fully inactivated dead Cas9 protein (dCas9) to the catalytic domain of the *FokI* endonuclease. The fdCas9 protein can be guided to the target sites using two gRNA molecules that bind to the sense and antisense strands in forward and reverse conformations. When the gRNA molecules are in close proximity and the correct orientation, they facilitate homodimer formation of the FokI catalytic domain and thus DSB formation within the intervening sequence. The synthetic fdCas9 chimeric protein was capable of mediating efficient genome editing with improved specificity in mammalian cells. In conclusion, our data show that fdCas9 provides a superb alternative to existing platforms and may overcome limitations that have prevented their use in a wide variety of genome engineering applications.

## Results

### Design and construction of synthetic fdCas9 endonuclease variants

**Fig 1:**
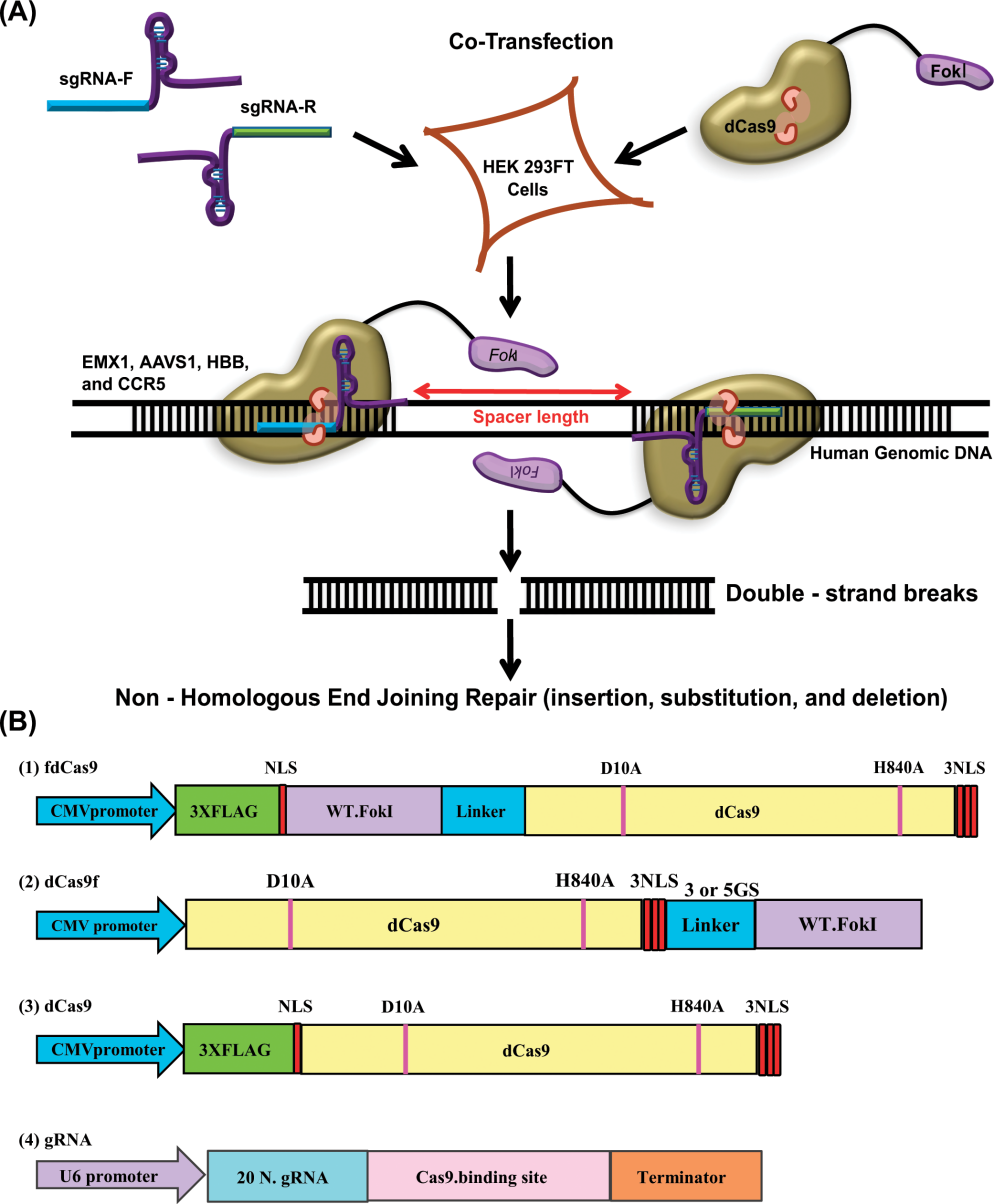
Schematic representation of different dCas9 and FokI fusion variant architectures. (A) Schematic strategy used to test dCas9 and FokI fusion variants for homodimer formation and double-strand break (DSB) generation within the target sequence. A pair of gRNAs capable of guiding the dCas9 and FokI fusion variants and binding to the sense and antisense DNA strands to facilitate dimerization of the FokI catalytic domain is shown. (B) Schematic representation of dCas9 and FokI fusion variants. The FokI catalytic domain was fused to either the C- or N-terminus of dCas9 with different linker sequences to facilitate dimer formation. The fdCas9 variant was cloned under the CMV promoter with a linker of 16 amino acids and 4 NLSs, one in the N-terminal domain and three in the C-terminal domain. dCas9 was also cloned under the CMV promoter and used as a negative control. NLSs were included on either or both ends of the fusion protein to boost its nuclear localization.

A Cas9 nuclease dead (dCas9) version was generated by simultaneously disrupting the HNH and the RuvC catalytic domains [37]. The dCas9 protein variant was incapable of cleaving the DNA but retained the ability to be targeted by gRNAs. Since the Cas9 nuclease and the Cas9 nickases exhibit significant off-target activity, we attempted to design and constructed dCas9.FokI protein variants by using the dCas9 as a DNA targeting module and the non-specific catalytic domain of the FokI endonuclease as a cleaving module. Such biomodular protein variants, with architectures reminiscent of ZFN and TALEN architectures, can be used to generate homodimers on a DNA target sequence defined by the specificities of gRNA sequences on sense and antisense strands and the length of the intervening spacer sequence, (Fig 1A) [7]. We used the previously reported dCas9 backbone and generated different C- and N-terminus fusions of the FokI catalytic domain [37]. To generate the dCas9.FokI chimeric protein, we PCR-amplified and cloned the full-length dCas9 fragment into the pENTR/D TOPO vector (Life Technologies, Carlsbad, CA, USA) to facilitate subcloning and gateway recombination into a pDEST26 destination vector for mammalian cell expression (S1 File for sequencing data and map). To produce an in-frame dCas9 C-terminus fusion, we sub-cloned synthetic fragments composed of the catalytic domain of wild-type FokI preceded by three nuclear localization signals (3NLS) and a linker sequence (Fig 1B and S1 File). To produce an N-terminus fusion, we cloned a synthetic fragment composed of 3XFLAG, 1 NLS, 2GS, the wild-type FokI catalytic domain, and linker sequences into the N-terminus of dCas9 using the NcoI restriction enzyme (Fig 1B and S1 File). Subsequently, we cloned different C- and N-terminus fusions of dCas9 and FokI in pENTR/D into pDEST26 under the control of the *CMV* promoter for mammalian cell expression (Fig 1B). We tested dCas9 and FokI fusion variants for dimerization and catalytic activity on episomal and genomic DNA targets.

### fdCas9 endonuclease variant exhibited robust catalytic activity in surrogate reporter system assays

**Fig 2:**
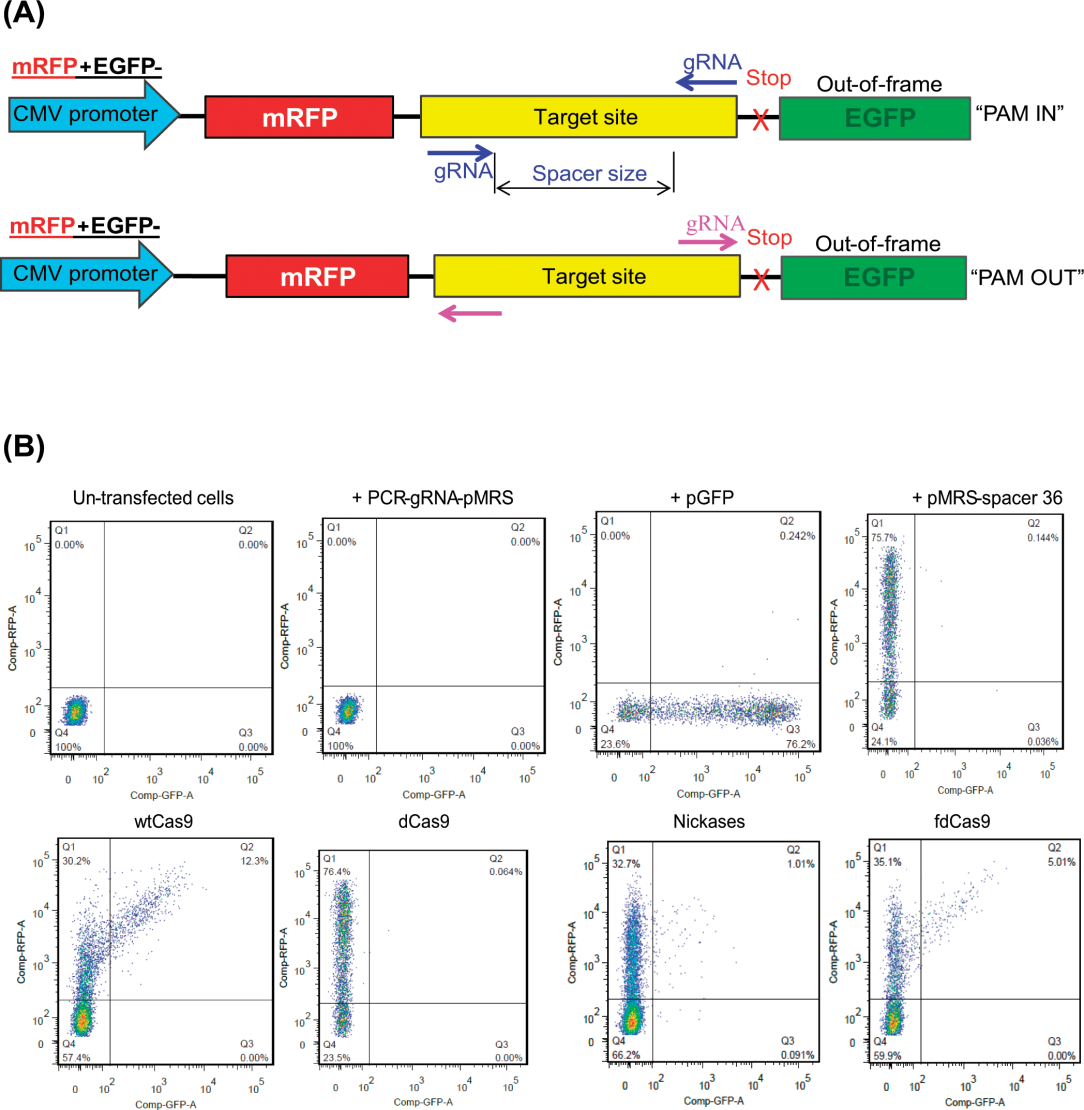
fdCas9 fusion variant exhibited robust catalytic activity in surrogate reporter assays. (A) Schematic representation of the pMRS plasmid used in surrogate reporter assays. Different versions were used including those with different spacer lengths in the intervening sequence between the monomeric red fluorescent protein (mRFP) and enhanced green fluorescent protein (eGFP) reporters. gRNA pairs in PAM-in and PAM-out orientations were tested. (B) Flow cytometry of HEK293 cells at 3 d post-co-transfection with fdCas9 and the PCR amplicon of gRNA under the U6 promoter with a 36-bp spacer target. Paired Cas9 nickases and wtCas9 were used as positive controls and dCas9 as a negative control. The percentage of cells expressing both mRFP and eGFP is shown in the Q2 area of each panel.

The catalytic activities of heterodimeric ZFNs and TALENs are sensitive to spacer length [5, 7]; therefore, we sought to determine the genome editing efficiency of different N- and C-terminus variants. We used a surrogate reporter system with various spacers (i.e., sizes) and orientations (i.e., protospacer adjacent motif (PAM)-in and PAM-out) (Fig 2A and S1 File) [38–40]. Briefly, this system was composed of two red and green fluorescent proteins (RFPs and GFPs) with their coding sequences separated by an intervening target sequence that contains a stop codon and renders GFP out of frame (Fig 2A). Targeted DSBs in the intervening sequence due to nuclease activity followed by non-homologous end joining makes 1/3 of the repair events in frame with the second GFP reporter, resulting in functional copies and green fluorescence. We transfected HEK293 cells with the DNA of dCas9 and FokI variants, pairs of gRNAs targeting the fusion proteins to the intervening sequence, and the pMRS reporter plasmid. All gRNA pairs were designed to bind in either PAM-in or PAM-out orientations, bringing the two dCas9.FokI monomers in close proximity to allow FokI dimer formation and subsequent catalytic activity (S1 File). Since FokI activity is dependent on dimerization of the FokI domain, we designed various combinations of forward and reverse gRNAs to test various spacer lengths with PAM-in and PAM-out orientations (S1 File). It has been reported previously that the optimal spacer length for ZFNs is 6–8 bp and for TALENs is 16–24 bp [7]. We therefore designed spacer lengths that ranged from 2–39 bp (S1 File). Binding of the dCas9 and FokI fusion monomers that permit dimer formation of the FokI domain results in DSBs, frameshift mutations, and the rescue of GFP expression.

We measured GFP fluorescence 72 hours post-transfection using flow cytometric analysis to determine the level of nuclease activity. For all experiments, we used dCas9 as a negative control and wild-type (wt) Cas9 and Cas9 nickases as positive controls for analyses of activity and to ensure the proper design and capability of gRNAs in targeting Cas9 monomers. Using the surrogate reporter system, we confirmed the cleavage activities of wtCas9 and paired nickases, as well as the lack of dCas9-mediated cleavage (Fig 2B and S1 File). The C-terminus fusions dCas9f did not exhibit any detectable catalytic activity in either PAM-in or PAM-out orientations, for all spacer lengths (data not shown). Conversely, the N-terminus fusion fdCas9 exhibited robust catalytic activity in the PAM-out orientation, though only for spacer lengths ranging from 15–39 bps. Surprisingly, the catalytic activity of the fdCas9 variant was better than that of the Cas9 paired nickases and very close to that of wtCas9 (Fig 2B).

### fdCas9 endonuclease exhibits robust catalytic activity on genomic DNA

**Fig 3:**
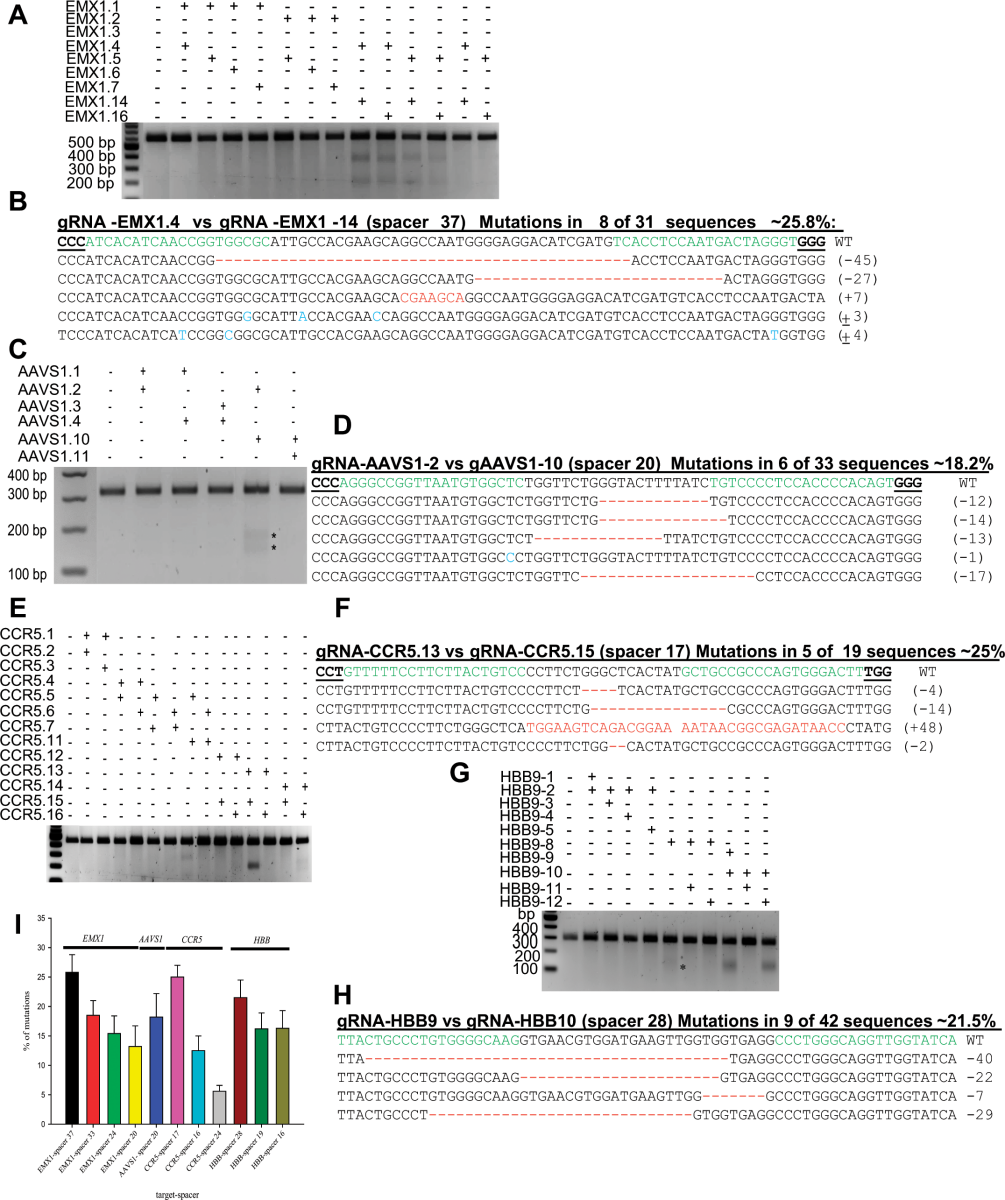
Robust catalytic activity of the fdCas9 variant on endogenous genomic targets. (A, C, E, and G) T7EI assays for the *EMX1*, *AAVS1*, *CCR5*, and *HBB* genomic targets, respectively with fdCas9 using several combinations of gRNAs in PAM-in and PAM-out orientations. Arrows in (C) indicate the expected size of the DNA bands of *AAVS1* amplicons cleaved by T7EI. (B, D, F, and H) Alignment of Sanger sequencing reads of PCR amplicons encompassing the *EMX1*, *AAVS1*, *CCR5*, and *HBB* target sequences showing indels within the 37-, 20-, 17-, and 28-bp spacer sequences, respectively. gRNA targets are highlighted in green, the PAM sequence is shown in bold and underlined, dashes indicate nucleotide deletions, nucleotides highlighted in red indicate insertions, and nucleotides highlighted in blue indicate substitutions. Mutation frequencies were estimated as the number of mutant clones divided by the total number of sequenced clones. (I) Catalytic activities of fdCas9 on different genomic targets using gRNA pairs with different spacer sizes (represented in percentage).

We included all FokI.dCas9 fusion variants to test and confirm whether the catalytic activity and target specificity were similar to those observed in the surrogate reporter system assays. To this end, we selected four genes, *CCR5*, *HBB*, *AAVS1*, and *EMX1*, and employed the T7 endonuclease I (T7EI) surveyor assay to determine the catalytic activities of the dCas9 and FokI fusion variants, as previously described [30]. Our T7EI assays demonstrated activity of wtCas9 and some activity of the paired nickases, the positive controls, and no detectable activity of dCas9, the negative control, confirming correct gRNA designs and as well as the validity of the T7EI assay (Figs A and D in S1 File). Furthermore, our data on the modification of the four genomic targets corroborate our previous data obtained using the surrogate reporter system (Fig 2B). None of the dCas9f variants exhibited any catalytic activity on any genomic target for various spacer lengths in PAM-in and PAM-out orientations (Fig D in S1 File). In contrast, the fdCas9 variant produced robust catalytic activity on the genomic targets only in the PAM-out orientation of the dual gRNAs, consistent with our data using the surrogate reporter system (Fig 3A, 3C, 3E and 3G for *EMX1*, *AAVS1*, *CCR5*, and *HBB*, respectively). It is worth noting that the fdCas9 variant exhibited robust catalytic activity for different spacer lengths (17–37 bp) on genomic targets (Fig 3). No detectable activity was observed for the fdCas9 variant with a single gRNA, verifying that fdCas9 monomers were catalytically inactive (Fig C in S1 File). To precisely determine the cleavage site and the nature of indels, we PCR-amplified fragments encompassing the target site only with the combination of gRNAs that exhibited the highest fdCas9 activity for *EMX1*, *AAVS1*, *CCR5*, and *HBB* and cloned the amplicons into plasmid vectors using the TOPO TA Cloning Kit for Sequencing (Life Technologies). Based on Sanger sequencing data for these clones, fdCas9-induced DSBs were within the spacer sequence and resulted in indels of various lengths (Fig 3B, 3D, 3F and 3H for *EMX1*, *AAVS1*, *CCR5*, and *HBB*, respectively).

### fdCas9 variant possesses improved specificity in genome editing

**Fig 4:**
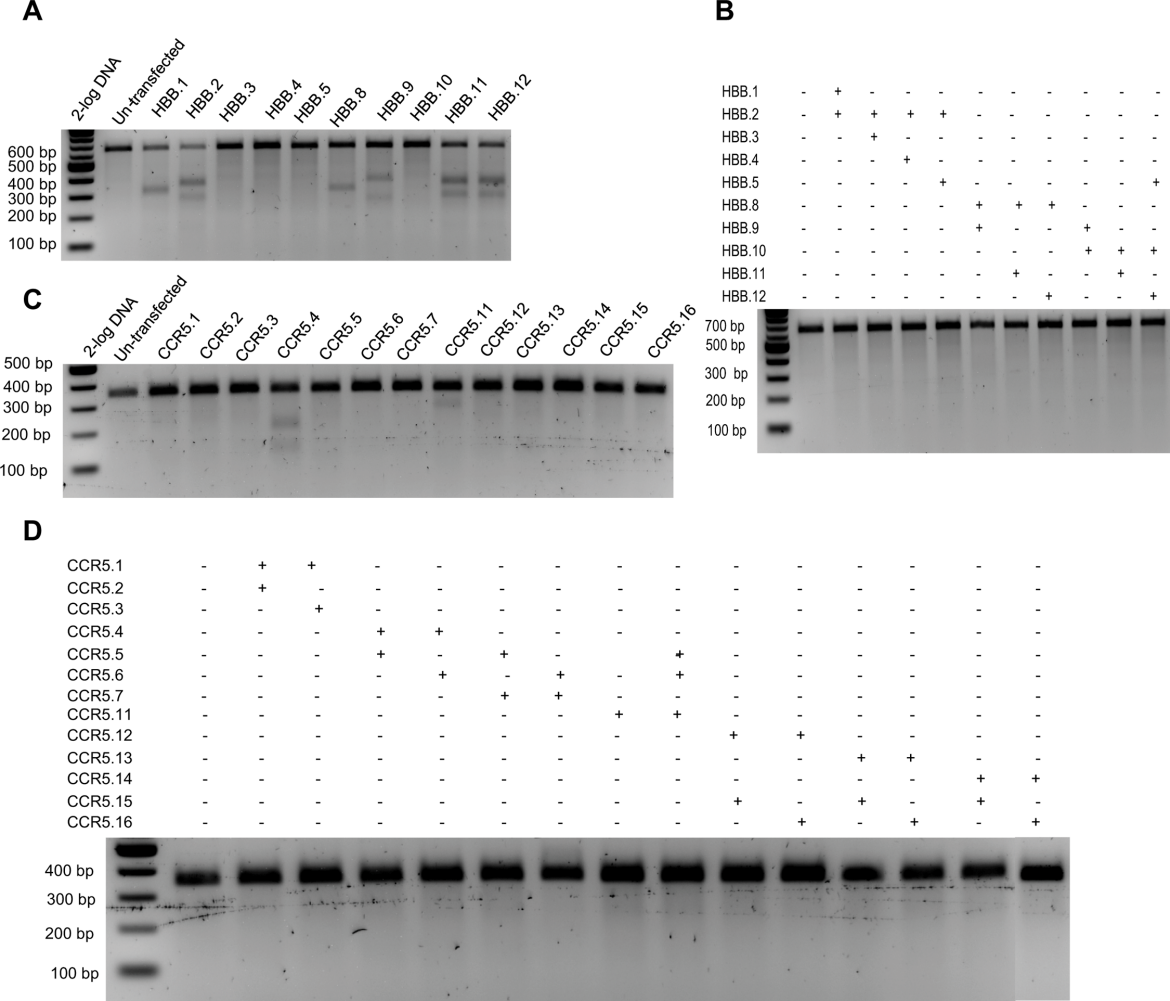
fdCas9 exhibited robust activity with high target specificity. The genome modification activities of fdCas9 were assayed at known Cas9 off-targets. (A) T7EI assays at *HBD*, a known off-target for the *HBB* genomic target, showed high modification frequencies using wtCas9 with various gRNAs targeting *HBB*. (B) T7EI assays at *HBD* showed no detectable modification activities using fdCas9. (C) T7EI assays at *CCR2*, a known off-target for the *CCR5* genomic target showed high modification frequencies with wtCas9 using various gRNAs targeting *CCR5*. (D) T7EI assays at *HBD* showed no detectable modification using fdCas9 guided by different combinations of gRNAs.

To investigate whether fdCas9 catalytic activity is reduced at potential genomic off-target sites, we selected several genomic targets with known off-targets. As we have previously reported, wtCas9 exhibits substantial genomic modification at off-target sequences [30]. Therefore, we designed gRNA pairs with optimal spacer lengths for efficient fdCas9 activity at *HBB* and *CCR5* that were capable of binding to the *HBD* and *CCR2* off-targets as single gRNAs. Furthermore, we selected gRNA pairs with spacer lengths ranging from 31–39 bp. We included paired nickases and wtCas9 to compare the specificities of these reagents at the on- and off-target sites to ensure that each gRNA could direct Cas9 cleavage at the intended sites. As expected, wtCas9 produced substantial off-target activities ranging from 25% to 30% for different targets, *CCR5* and *HBB* sites, as previously reported (Fig 4A and 4C and A and Fig G in S1 File). In contrast, the fdCas9 variant did not exhibit any catalytic activity at the off-target sequence. Moreover, the Cas9 nickases did not exhibit detectable off-target activity, as previously reported [35]. It might be possible to detect off-target events using deep sequencing coverage of the target sites. Nevertheless, fdCas9 is highly specific when compared to wtCas9.

**Fig 5:**
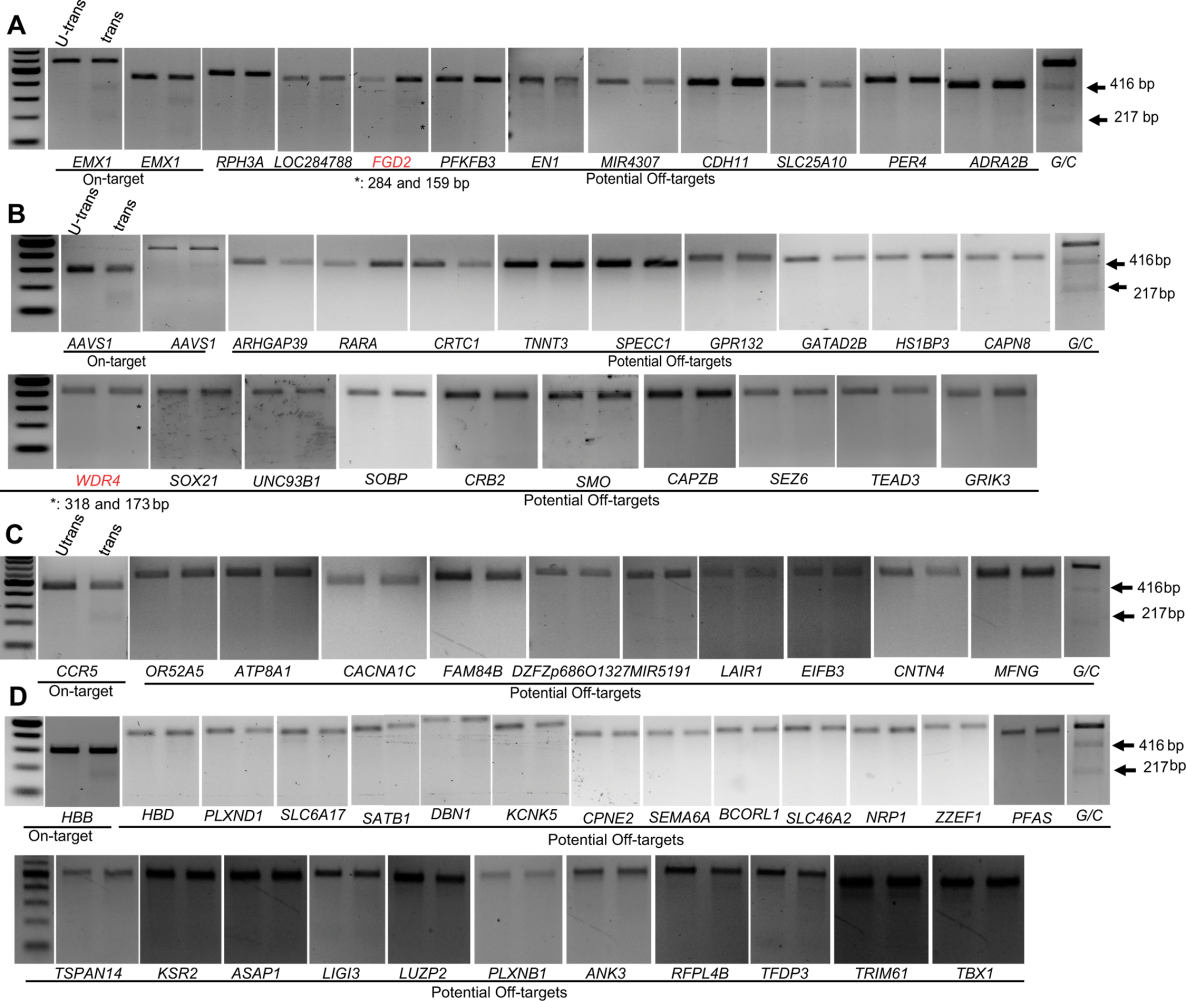
fdCas9 exhibited significantly improved specificity. T7EI assays to determine the catalytic activity of fdCas9 on potential off-targets, identified by PROGNOS web-tool, for *EMX1*, *AAVS1*, *CCR5* and *HBB* genomic targets. T7EI mutation detection assays for potential off-targets of EMX1.4 and EMX1.14 gRNA pair (Fig 5A). T7EI mutation detection assays for potential off-targets of AAVS1.2 and AAVS1.10 gRNA pair (Fig 5B). T7EI mutation detection assays for potential off-targets of CCR5.13 and CCR5.15 gRNA pair (Fig 5C). T7EI mutation detection assays for potential off-targets of HBB9 and HBB10 gRNA pair (Fig 5D). Note: * indicates the expected size of the DNA bands of corresponding amplicons cleaved by T7EI.

Moreover, we employed our recently developed PROGNOS web-tool to interrogate the genome for potential off-targets by allowing different mismatches (up to 6 nts) and spacer lengths (0–50 nts). We used the gRNA pair that produced the highest catalytic activity on each genomic target (Fig 3I). We identified 10 potential off-targets for *EMX1*, 19 off-targets for *AAVS1*, 9 off-targets for *CCR5*, and 24 off-targets for *HBB* (Tables I-L in S2 File). We used the T7EI assay to test the catalytic activity at each potential off-target. Our data reveal that the fdCas9 exhibited significant improvement of specificity. For example, fdCas9 did not exhibit any catalytic activity on *CCR5* and *HBB* off-targets (Fig 5C and 5D). However, very weak activity has been observed with 1 off-target of *EMX1* and *AVVS1* (Fig 5A and 5B). Therefore, fdCas9 exhibited a significant specificity compared to wtCas9.

## Discussion

The RNA-programmable Cas9 system holds much promise in genome engineering especially in genomic medicine applications. However, such applications are hampered by substantial off-target activity of the Cas9 endonuclease. Thus, attempts have been made to overcome this problem including paired Cas9 nickases and titrations of the Cas9 activities using different strategies including the control of the level and the efficiency of the gRNAs or Cas9 expression [35]. Paired Cas9 nickases provide a feasible solution to this problem but it is not without its drawbacks. Another approach involves the titration of Cas9 to control its expression or by controlling targeting efficiency by manipulating the gRNA length and structure [34, 41]. However, these approaches need further improvements in terms of specificity and efficiency for wider applications in genomic medicine. Generation of Cas9 architectures that necessitate more specific binding requirements for catalytic activity would substantially broaden the applications of precise genome engineering.

Here, we describe a robust and highly efficient system for genome engineering applications that overcomes the off-targeting problems associated with the present platforms. We developed a synthetic chimeric endonuclease variant (fdCas9), a fusion of the FokI catalytic domain and dCas9, that exhibits robust catalytic activity in a surrogate reporter system and on genomic targets with spacer lengths from 15–39 bp. Using two gRNAs, an obligate homodimer formed around the spacer sequence that brought the fdCas9 monomers of the FokI catalytic domain into close proximity and allowed their dimerization and catalytic activity, resulting in DSBs within the spacer sequence. The fdCas9 variant is quite similar to recently reported fCas9 and RFN endonucleases [42–44]. The NLS-GGS-FokI-XTEN-dCas9 variant exhibited 10% modification efficiency compared with 15% and 25% for Cas9 and wtCas9, respectively [43]. Our fdCas9 exhibited up to 20% modification efficiency in the surrogate reporter system compared with 15% for paired nickases, and possessed robust catalytic activity on a wider range of long spacer sequences (15–39 bp). This elevated catalytic activity might be attributed to the fdCas9 architecture and the presence of 1 NLS at the N-terminus and 3 NLSs at the C-terminus [18, 45]. The efficiency of the fdCas9 could be further boosted using the recently described tRNA processing system for multiple gRNAs [46]. In our experiments, fdCas9 variant exhibited robust catalytic activity, albeit slightly lower than that of wtCas9, but higher than that reported for fCas9 and RFN, indicating that several parameters still need to be optimized to produce architectures that possess equal catalytic activity to that of wtCas9 with improved specificity. Such improved catalytic activity of obligate homodimers with dual single-guide (sg) RNAs would substantially improve and extend the applications of CRISPR/Cas9 for precise genome engineering in mammalian cells and for genomic medicine, where specificity is an essential requirement.

We have previously reported that Cas9 exhibits off-target activity, including DNA and sgRNA bulges in addition to base mismatches [30, 32]. Although our results indicate that the specificity of the fdCas9 variant is similar to that of Cas9 paired nickases, a detailed genome-wide study will be needed to confirm this. Indeed, researchers have reported that the Cas9 nickase using single gRNAs exhibits lower modification levels than that observed using paired nickases, indicating that the latter increases the overall off-targets and could lead to mutagenic effects in the genome via unknown mechanisms [44]. The fdCas9 variant and the previously reported fdCas9 and RFN variants are ideal for precise and efficient genome engineering because they are active only when two gRNAs are in the PAM-out orientation with a spacer sequence length of 15–39 bp. This range of spacer lengths increases the number of targetable sequences in the mammalian genome. Future studies should focus on a detailed genome-wide analysis to assess the off-target activities and specificities of fdCas9 variants and paired nickases. Such analysis could help generate highly robust and specific fdCas9 variants with flexible spacer lengths to increase the number of targetable sites in the genome.

We attempted to further improve the catalytic activity of fdCas9 by using the FokI- Sharkey variant but we did not demonstrate a significant increase in the catalytic activity of Sharkey fdCas9 compared to fdCas9 (Fig I in S1 File) [47]. It might be possible to increase the efficiency of fdCas9 catalytic activity using additional manipulations such as optimizing the linker length and the PAM sequence and Cas9 backbone. While this study was in preparation, the same linker used in our fdCas9 variant was reported [43, 44]; however, the fdCas9 variant described here possesses elevated catalytic activity, which is likely due to the permissibility of homodimer formation using this architecture. Moreover, the spacer length required for robust catalytic activity of this fdCas9 variant ranged from 15–39 bp or higher. Longer spacers need to be tested to determine the exact spacer range. However, this fdCas9 variant exhibits robust catalytic activity and is sufficient for most genome engineering applications. Accordingly, future improvements should focus on the use of short versions of Cas9 to mediate DNA targeting, thereby improving delivery into different cells types. The fdCas9 endonuclease variant provides a versatile genome-editing tool, with improved specificity and catalytic activity, for precise engineering of mammalian genomes.

## Material and Methods

### Cell culture media, conditions and, DNA transfections

HEK293FT cells were cultured in Dulbecco's Modified Eagle Medium (DMEM) supplemented with 10% fetal bovine serum in a 37°C humidified incubator with 5% CO2. Transient transfection of cells was performed with FuGene HD DNA Transfection Reagent (BioRad, Hercules, CA, USA) following the manufacturer's instructions. Briefly, 80,000 HEK293T cells per well were seeded in a 24 well culture plate and cultured in DMEM supplemented with 2 mM fresh l-glutamine 24 h prior to transfection. Subsequently, cells were transfected with 250 ng of PCR amplicons containing the U6 promoter and sgRNA including a 19-nt guide sequence, and chimeric 85-nt RNA (S1 File), and 500 ng of wtCas9, fdCas9 fusion variants, dCas9, or Cas9 nickase plasmids using 3.4 μl of FuGene HD (Promega, Madison, WI, USA). All primers used to amplify the gRNAs are described in Table G in S1 File and the gRNA sequences are listed in Tables C-F in S1 File for *EMX1*, *AAVS1*, *CCR5*, and *HBB*, respectively.

### Construction of dCas9 and FokI fusion variants

Human-codon-optimized catalytically inactive Cas9 (dCas9) was obtained from Addgene (Cambridge, MA, USA; Product number 44246) (https://www.addgene.org/44246/). dCas9 was sub-cloned into the pENTR-D/TOPO vector (Invitrogen, Paisley, UK) using NcoI and EcoRI restriction enzymes. Sanger sequencing was used to confirm the authenticity of the dCas9 clone using the set of primers highlighted in Table A in S1 File. To construct C-terminus fusions (dCas9-FokI), dCas9 was cloned into the pENTR-D/TOPO vector following the same procedure as the FokI-dCas9 N-terminal fusion and verified by Sanger sequencing using the primers described in Table A in S1 File. The MluI/EcoRI C-terminus fragment of dCas9 was amplified by PCR with modified reverse primers (Table A in S1 File) to insert ApaI immediately before the EcoRI sites and remove the stop codon, keeping the NLS from the EcoRI site, using the Phusion Polymerase from New England Biolabs (Ipswich, MA, USA). Next, the MluI/EcoRI PCR fragment was re-inserted back into the original backbone to generate a new backbone with ApaI sites added and the stop codon removed. The FokI catalytic domain from dHax3 was PCR-amplified with forward primers containing the ApaI site and reverse primers containing the EcoRI site and a stop codon (Table A in S1 File) using the Phusion Polymerase from New England Biolabs. Finally, the FokI fragment was sub-cloned into the dCas9 backbone with a stop codon to generate the dCas9.FokI chimeric construct (S1 File). To construct N-terminus fusions, 3FLAG-NLS-WT.FokI-L16 fragments (Supplementary information; Gene synthesis from Blue Heron, Bothell, WA, USA) in the N-terminal region of dCas9.3NLS were custom synthesized in the pENTR/D plasmid using the NcoI restriction enzyme. The N-terminal fusion of 3FLAG-NLS-WT.FokI-L16 was then confirmed by sequencing using one reverse primer, dCas9-R (5′-CGGGTTGCTTCAGCGGTCTCCC-3′), and subsequently cloned into the pDEST26 human expression vector by LR-Gateway recombination cloning (S1 File).

### Episomal surrogate reporter assays

The RFP-GFP reporter plasmids used in this study were constructed as described previously [38]. The target DNA with various spacer lengths between the two gRNA binding sites was cloned into the pMRS plasmid between the EcoRI and BamHI restriction enzyme sites using primer cloning (Table B in S1 File for the PAM-out orientation). Sanger sequencing was used to confirm the cloning of the targets using primers described in Table A in S1 File.

To determine the catalytic activities of dCas9 and FokI C- and N-terminus fusion variants, HEK 293FT cells were co-transfected with each fusion variant plasmid (500 ng) and the RFP-GFP reporter plasmid (200 ng) and 150 ng of gRNA PCR amplicon containing the U6 promoter in both orientations (PAM-in and PAM-out) and G followed by a 19-nt guide sequence 85-nt chimeric RNA in a 24-well plate using FuGene (Promega). At 3 d post-transfection, transfected cells were subjected to flow cytometry and cells expressing both RFP and GFP were counted and quantified.

### Mutation detection analysis using T7EI assays

Genomic DNA was purified 3 d post-transfection using QuickExtract (EpiCentre, Madison, WI, USA) [48]. T7EI mutation detection reactions were performed, as previously described [14], and the digestion products were resolved on 2% agarose gels. Unless otherwise stated, all PCR reactions were performed using high-fidelity AccuPrime Taq DNA Polymerase (Life Technologies) according to the manufacturer's instructions for 40 cycles (94°C, 30 s; 58–64°C depending on the target, 30 s; 68°C, 60 s) in a 50-μl reaction volume containing 1.5 μl of cell lysate, 3% dimethyl sulfoxide, and 1.5 μl of each 10 μM target region amplification primer or off-target region amplification primer (Table H in S1 File). To corroborate our T7EI assays, the PCR products were sub-cloned in TOPO TA Cloning or Zero Blunt vectors (Life Technologies), according to the manufacturer's instructions. Sanger sequencing reactions were performed using the M13F primer (5′-TGTAAAACGACGGCCAGT-3′).

### Off-target analysis for *CCR5* and *HBB*

*CCR5* and *HBB* off-targets, known as *CCR2* and *HBD*, respectively, were analyzed using a bioinformatics-based search tool to select potential off-target sites, which were evaluated using the T7EI mutation detection assay [30]. Sanger sequencing was used to confirm the gene modification frequencies for the CRISPR/Cas9 system, the nickases, and the chimeric FokI.dCas9. The primers used to amplify *CCR2* and *HBD* are listed in Table H in S1 File.

### Potential off-target analysis of fdCas9 using T7EI assays

Potential paired fdCas9 off-target sites in the human genome (hg19) were identified using the recently developed bioinformatics program PROGNOS, up to 6 base mismatches per target half site were permitted, with a spacer range of 0–50 bp and top ranked sites were further investigated [49]. Primers used for target region amplification were designed by PROGNOS (Tables I-L in S2 File for *EMX1*, *AAVS1*, *CCR5* and *HBB* respectively). The cleavage activity of RNA-guided fdCas9 at off-target loci was assessed based on the mutation rates resulting from the imprecise repair of DSBs by NHEJ. T7EI mutation detection assays were performed, as described previously and the digestion products were resolved on 2% agarose gels [48]. We used the G/C control provided in the SURVEYOR Mutation Detection Kit as a positive control for the T7EI assays [10, 50].

## Supporting Information

S1 File T7EI assays and sequencing information. (DOCX)

S2 File Potential off-targets of EMX1, AAVS1, CCR5 and HBB. (XLSX)
