# Supplementary material for: Efficient fdCas9 Synthetic Endonuclease with Improved Specificity for Precise Genome Engineering
Source: PLoS One. 2015 Jul 30;10(7):e0133373. doi: 10.1371/journal.pone.0133373 (PMC4520497; doi:10.1371/journal.pone.0133373)
Supplement: S1 File — (DOCX) [file pone.0133373.s001.docx]

SUPPLEMENTARY INFORMATION

Efficient fdCas9 Synthetic Endonuclease with Improved Specificity for Precise Genome Engineering Applications

Mustapha Aouida, Ayman Eid, Zahir Ali, Thomas Cradick, Ciaran Lee, Harshavardhan Deshmukh, Ahmed Atef, Dina AbuSamra, , Samah Zeineb Gadhoum, Jasmeen Merzaban, Gang Bao and Magdy M. Mahfouz*

*Author for correspondence: Magdy Mahfouz (Magdy.mahfouz@kaust.edu.sa)

| Supplementary Fig. A | T7EI assays at EMX1, AAVS1, CCR5, and HBB targets with wtCas9 |
| --- | --- |
| Supplementary Fig. B | Target genome modification of EMX1, AAVS1, CCR5, and HBB using wtCas9 and various gRNAs |
| Supplementary Fig. C | T7EI assays at EMX1, AAVS1, CCR5, and HBB with fdCas9 using a single gRNA |
| Supplementary Fig. D | T7EI assays at EMX1, AAVS, CCR5, and HBB targets with dCas9, Cas9 nickases, and dCas9f and a pair of gRNAs in PAM-in and PAM-out orientations. |
| Supplementary Fig. E | Target genome modification of EMX1, AAVS1, CCR5, and HBB using Cas9 nickases and a pair of gRNAs |
| Supplementary Fig. F | Comparison of the genome editing activities of fdCas9 and the Cas9 nickases with a pair of gRNAs in the PAM-out orientation. |
| Supplementary Fig. G | Target genome modification of HBD and CCR2 using wtCas9 and a pair of gRNAs for HBB and CCR5, respectively |
| Supplementary Fig. H | T7EI assays in HBD and CCR2 targets using Cas9 nickases |
| Supplementary Fig. I | Sharkey FokI variant displayed same activity as the wt.FokI |
| Supplementary Tables | Table A; B; C; D; E; F; G; H |
| Additional Supplementary information | pMRS reporter system, gRNA combinations of targets, Constructs, DNA and amino acid sequencing results for dCas9, fdCas9, and dCas9f variants |


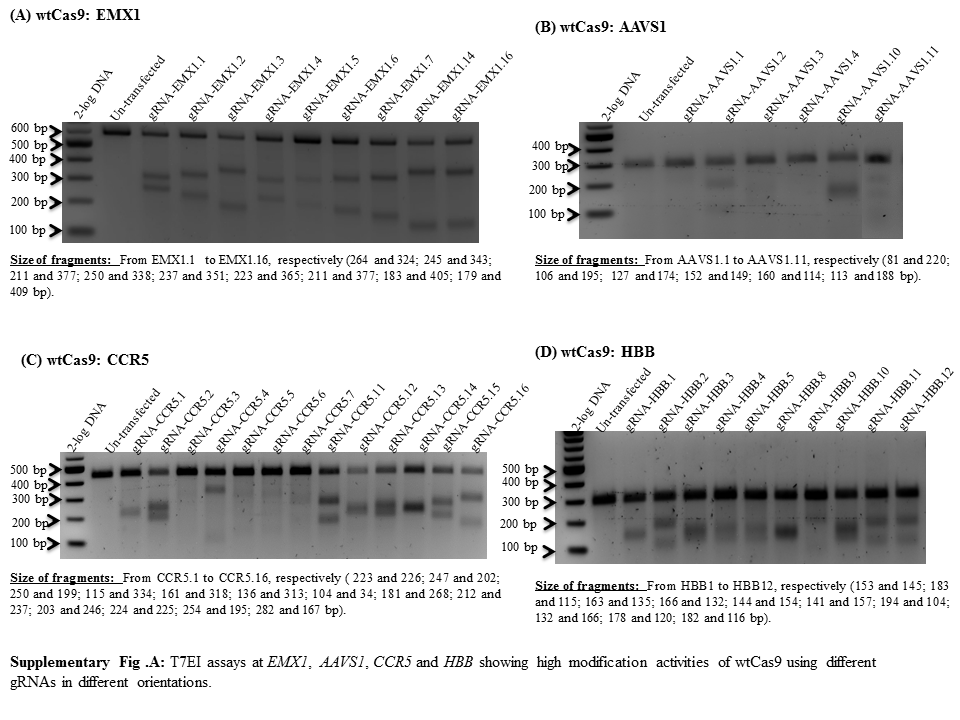


**
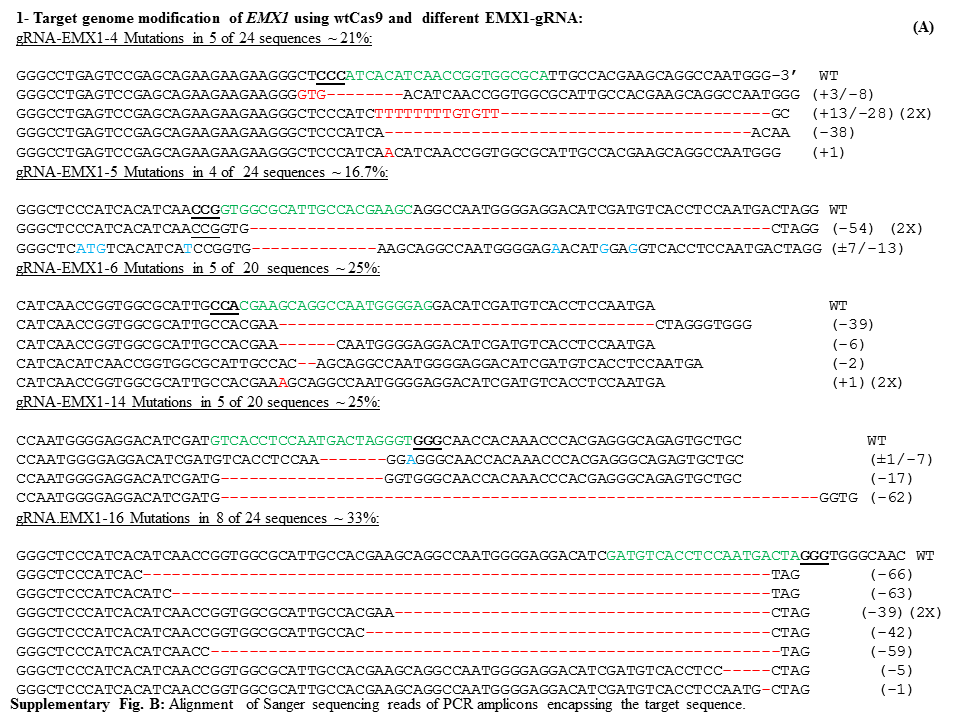
**

**
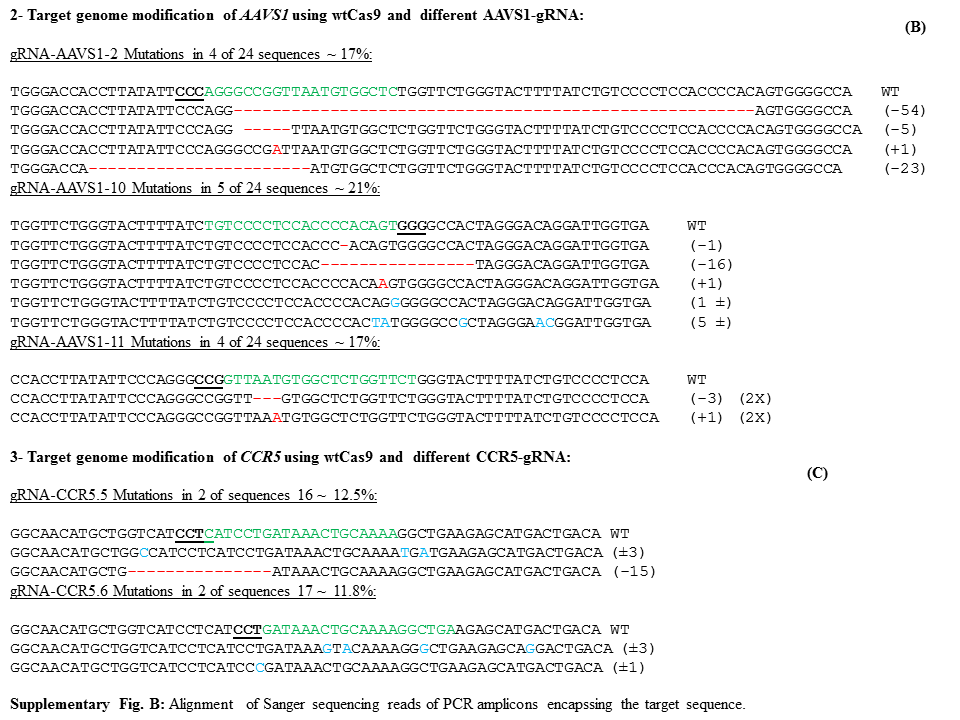
**

**
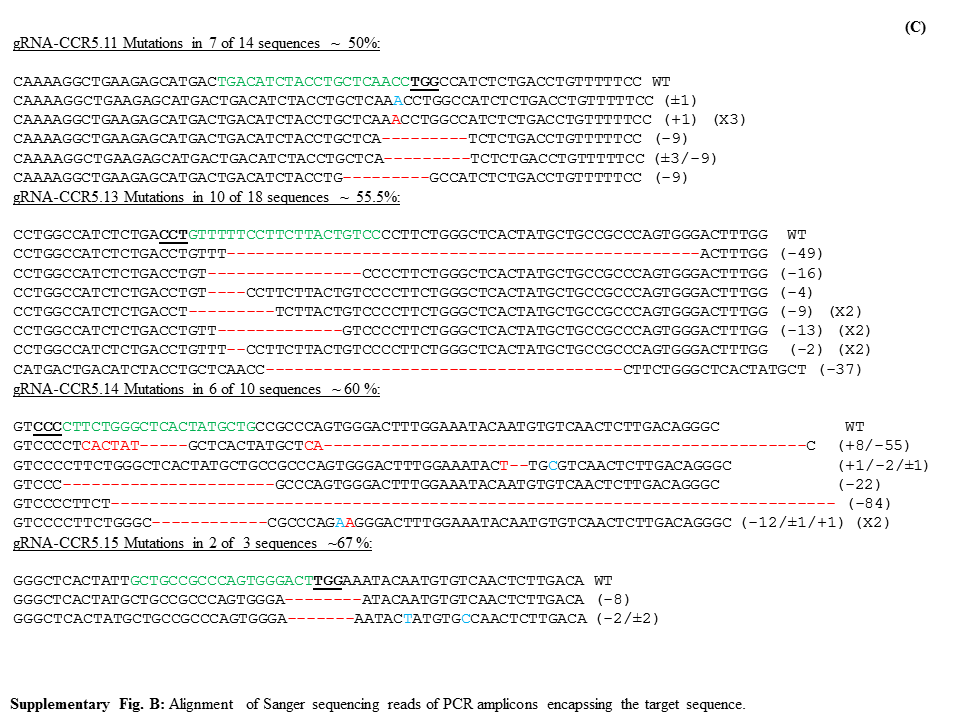
**

**
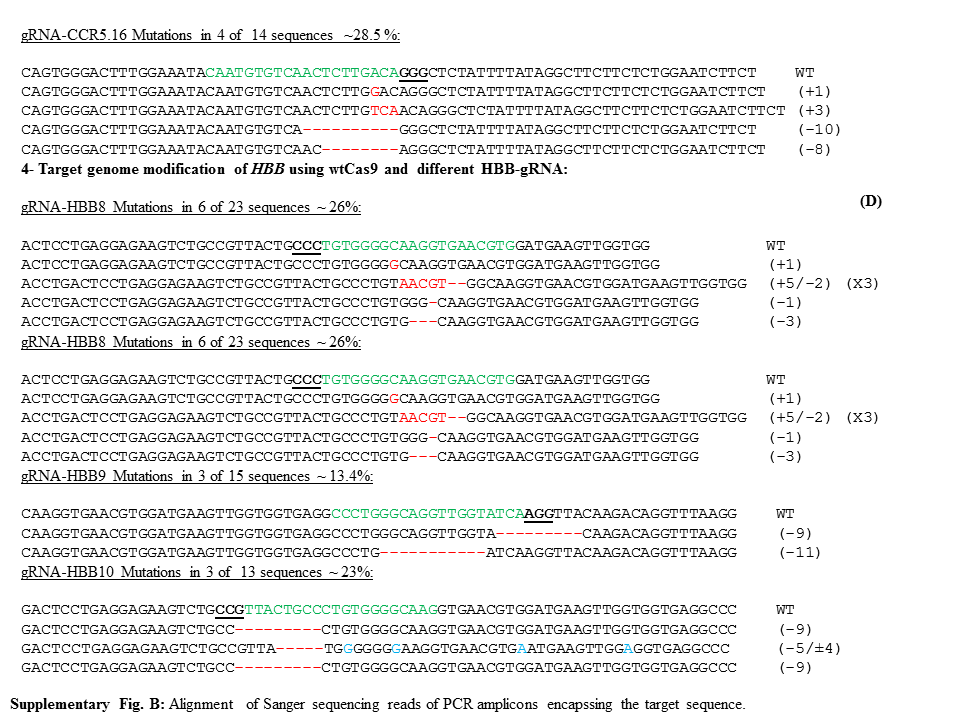
**

**
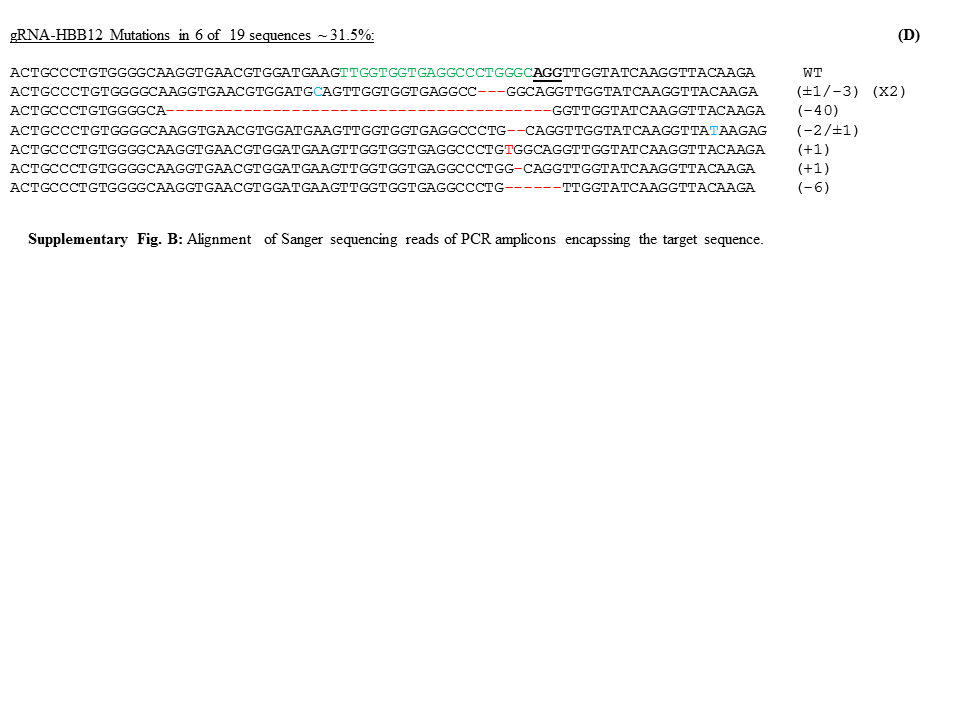
**

**
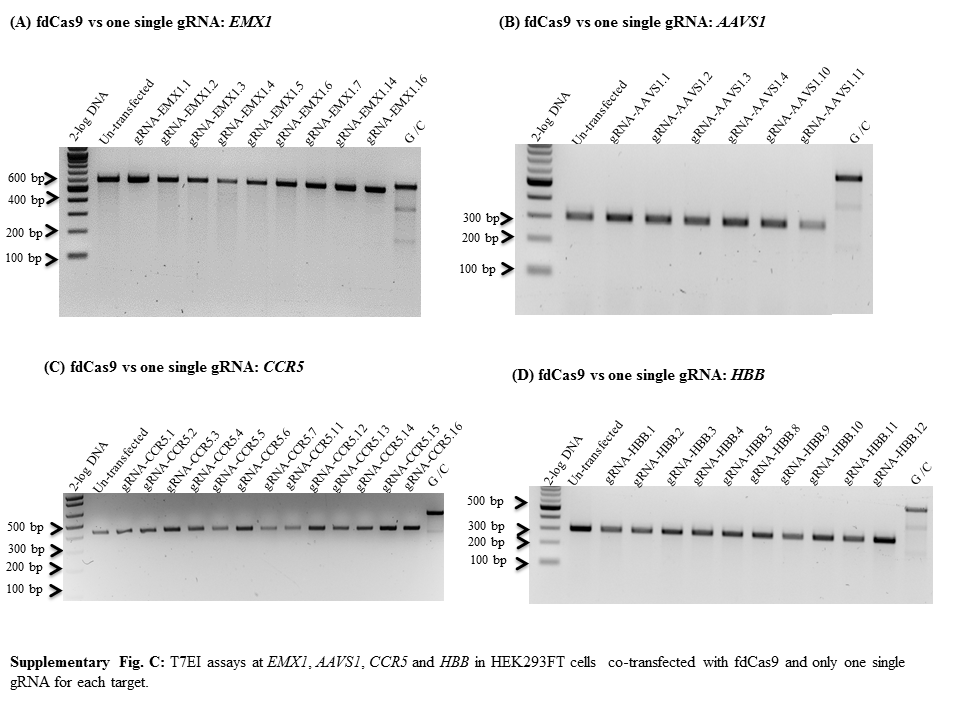
**

**
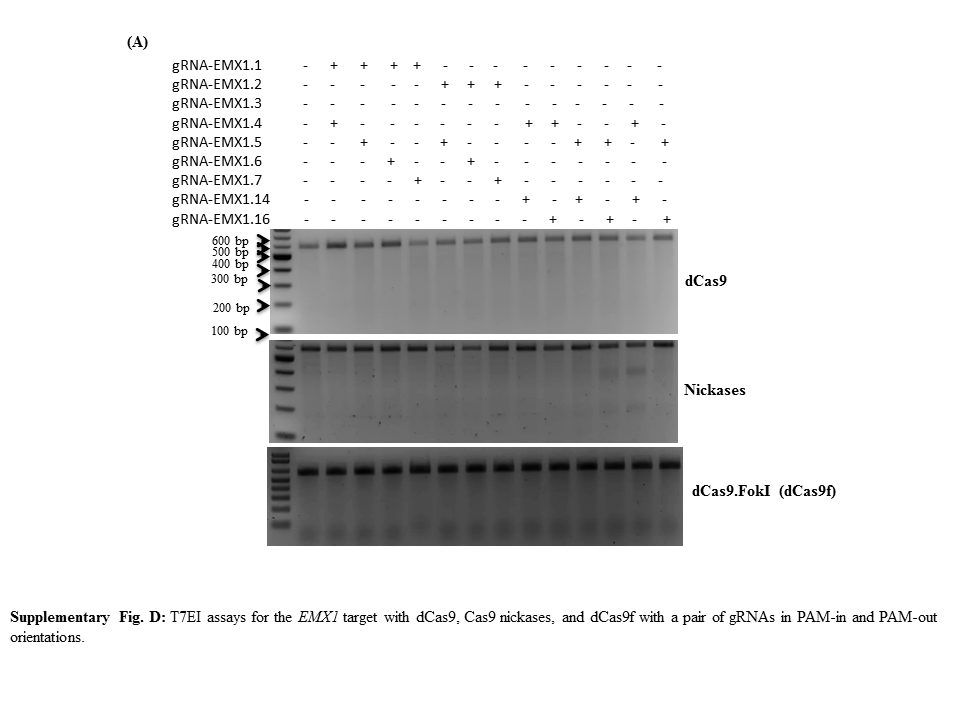
**

**
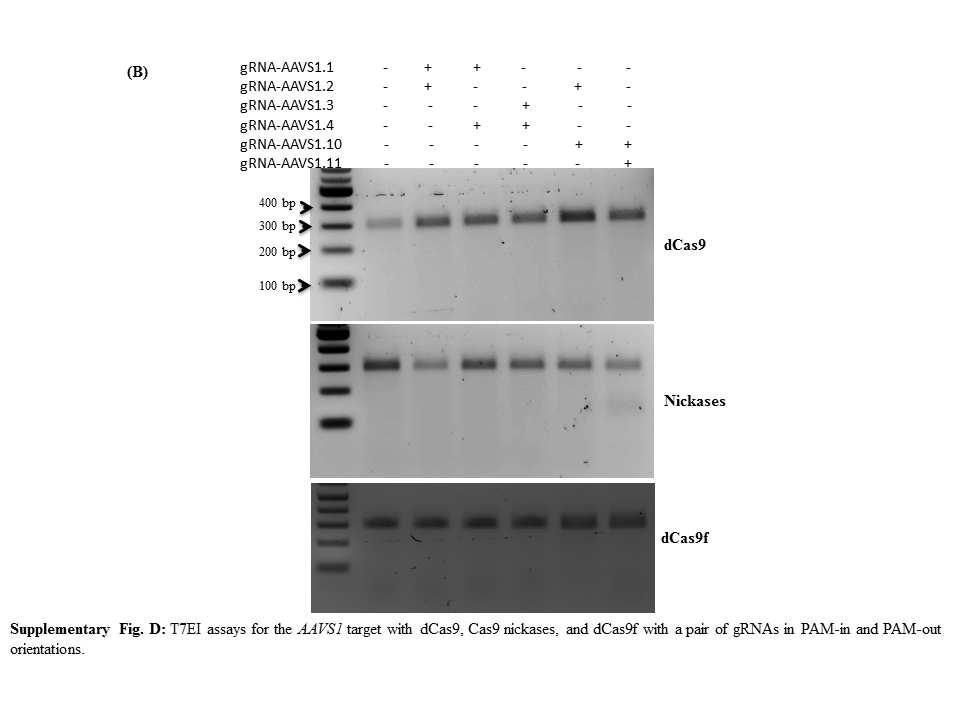
**

**
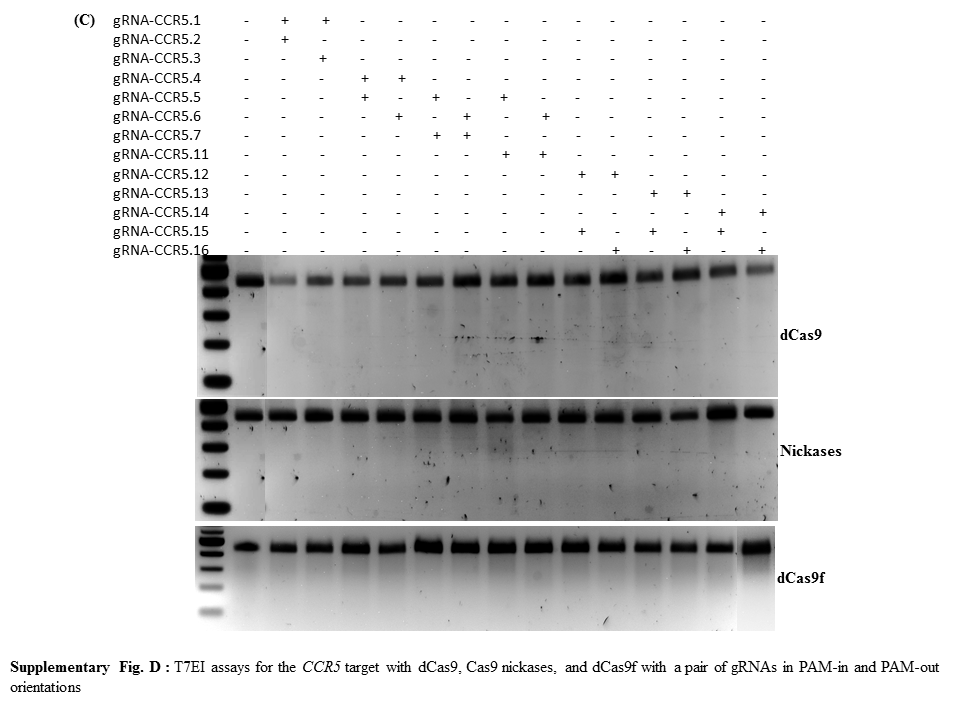
**

**
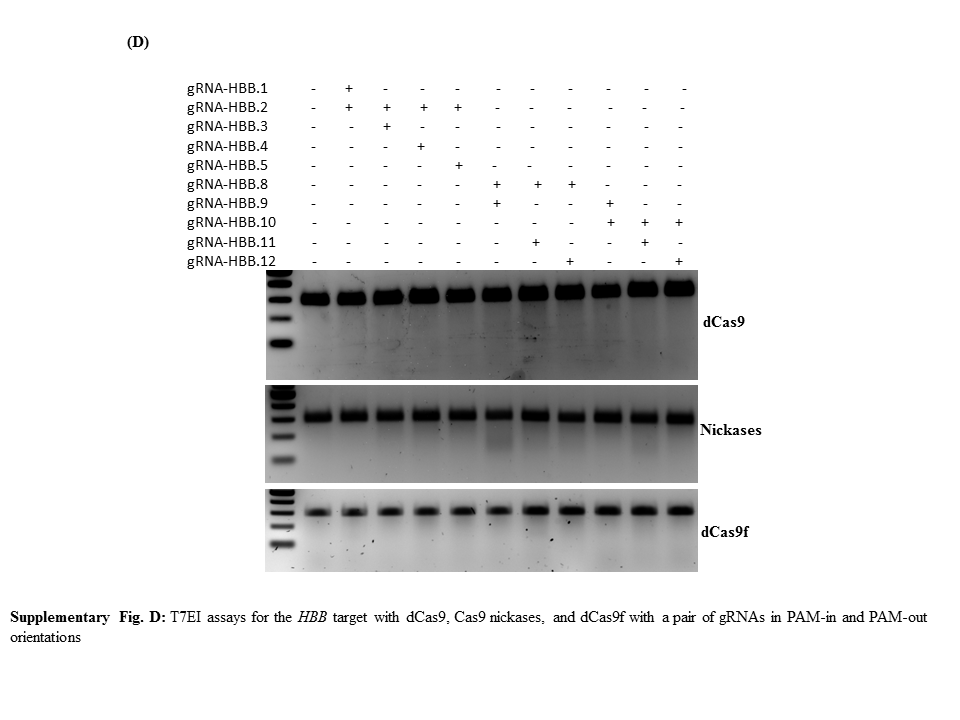
**

**
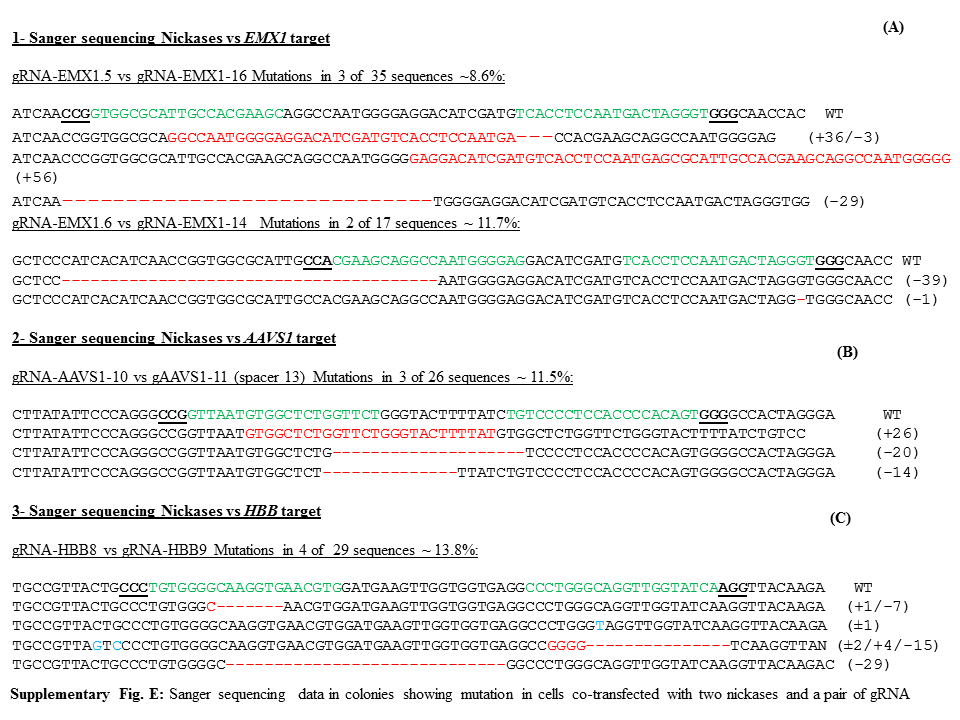
**

**
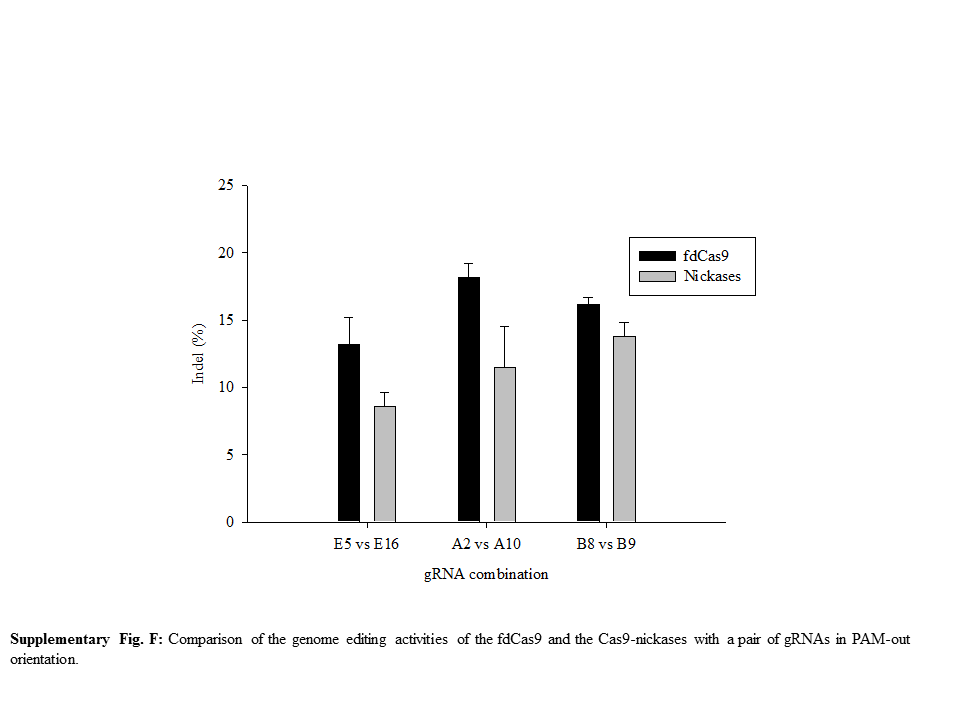
**

**
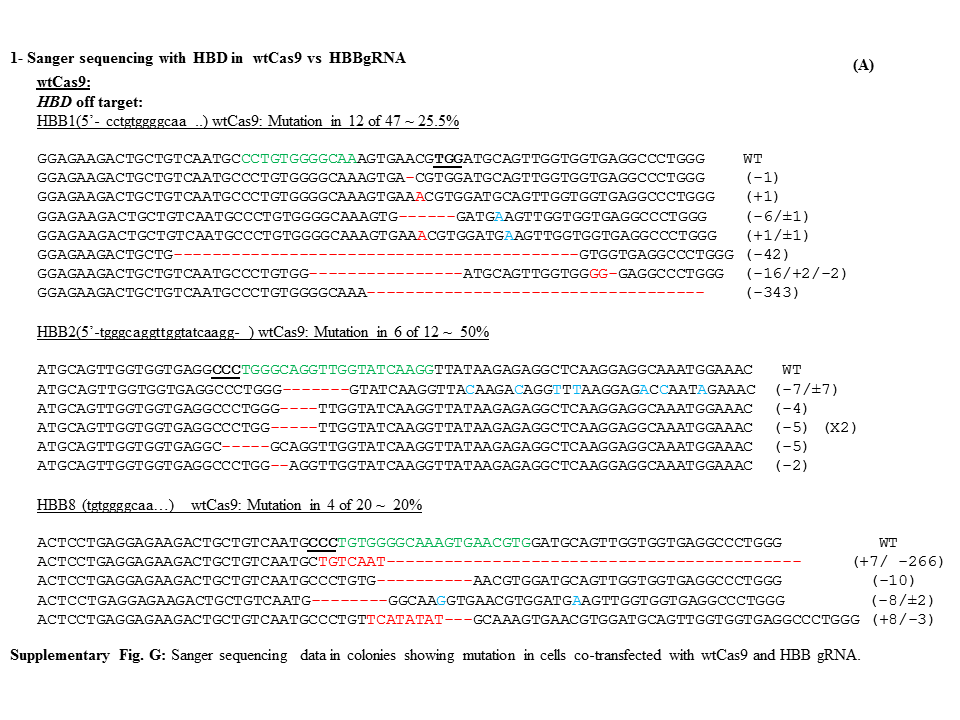
**

**
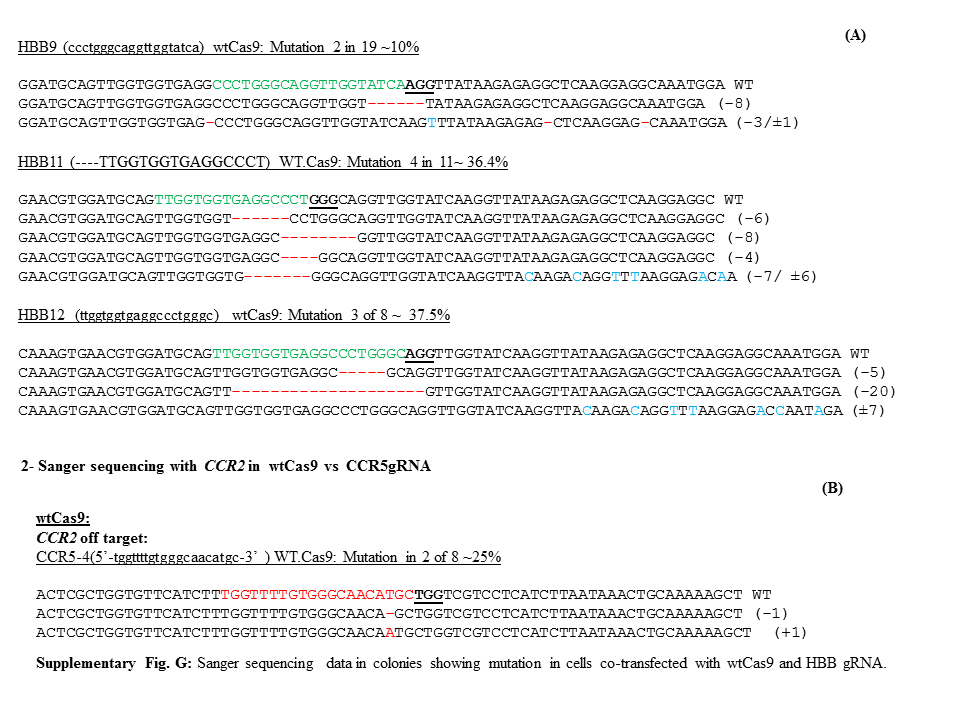
**

**
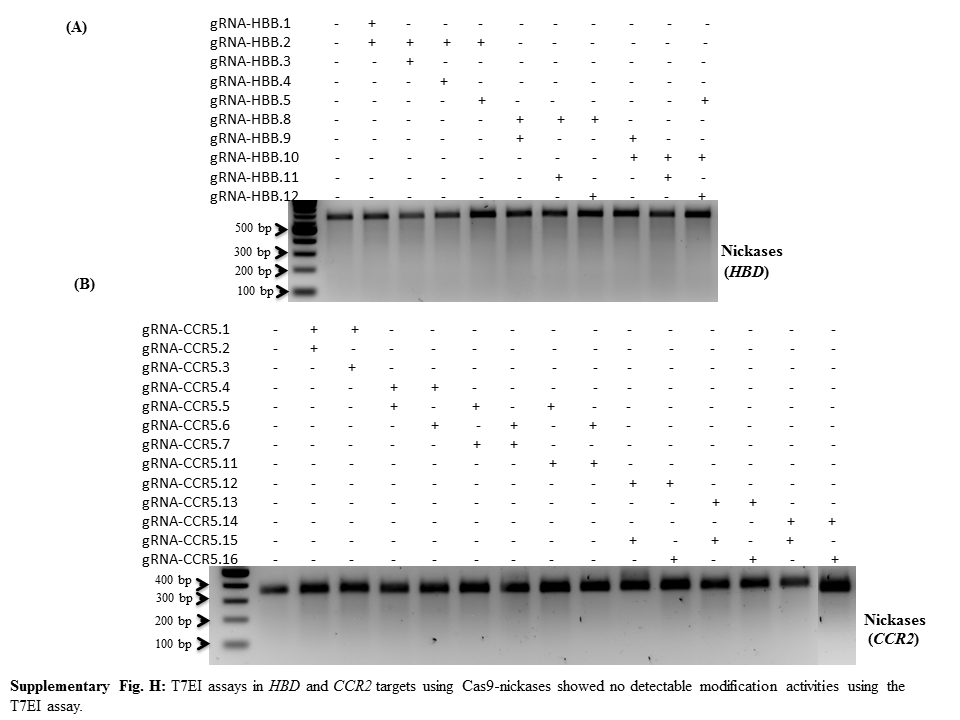
**

**
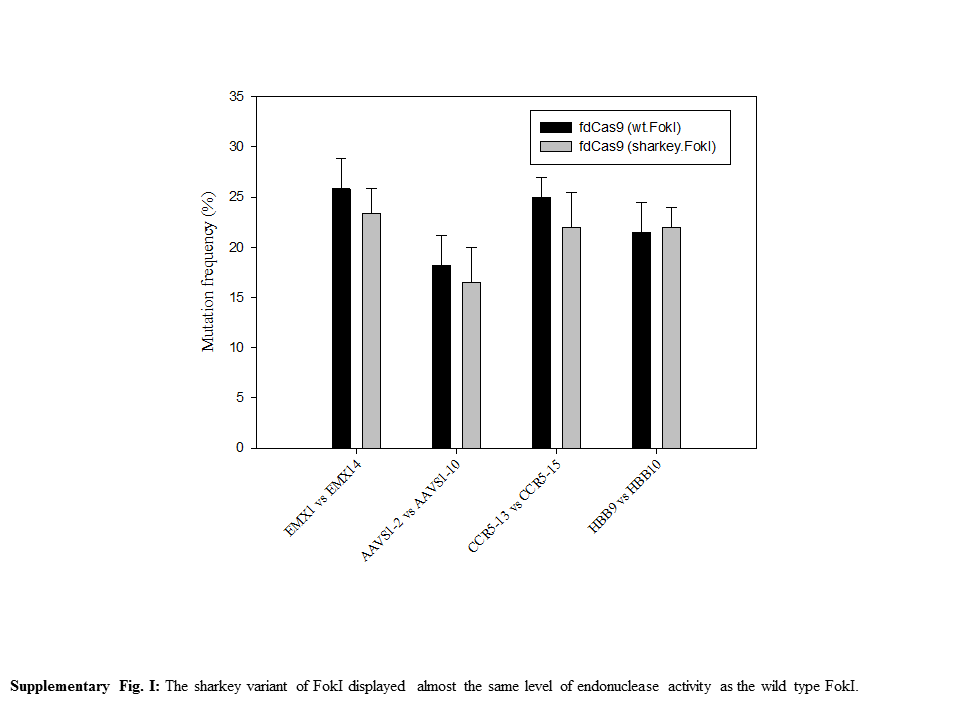
**

Table A: Primers used for cloning and sequencing of fdCas9, dCas9f, and different spacers for pMRS.

| Name | Primer Sequence | Used |
| --- | --- | --- |
|  |  |  |
| dCas9H.MluI.F | CCGAGTTTTCTAAACGCGTCATTCTCGCTGATGC | Amplify human dCas9 fragment MluI XhoI to remove the codon stop from dCas9 |
| dCas9H.RI.X.ApI.R | GCGGAATTCTCGAGGGGCCCTACCTTTCTCTTCTTTTTTGGATCTACC | Amplify human dCas9 fragment MluI XhoI to remove the codon stop from dCas9 |
| FokI.FApaI | ACGGGGCCCAAGCAACTAGTCAAAAGTGAACTGGAGGAGAAG | Amplify FokI to be inserted in human dCas9 |
| FokI.R.Xh1.R1 | GCGGAATTCTCGAGTCAAAAGTTTATCTCGCCGTTATTA | Amplify FokI to be inserted in human dCas9 |
| dCas9H. F1 | GTAATGAGCTGGCTCTCCCCTCCAAGTACGTG | To verify the fusion of human dCas9-FoKI |
| dCas9H. F2 | CCCTCATCCACCAGAGCATCACCGGACTTTAC | To verify the fusion of human dCas9-FoKI |
| dCas9H. R1 | GTACTTGAATGCGGCAGGGGCACCAAGATTGG | To verify the fusion of human dCas9-FoKI |
| dCas9H. FokI.R2 | CCTCTATATCCATAAACTTTCATAAAAAATTC | To verify the fusion of human dCas9-FoKI |
| pMRS.seqcF1 | GCTGGACATCACCTCCCACAACGAGGACTACACCAT | To verify the spacer cloning |
| pMRS.seqcR1 | GAAGAAGTCGTGCTGCTTCATGTGGTCGGGGTAG | To verify the spacer cloning |

Primers with restriction EcoRI and BamHI sequence over-hangs used for spacer cloning:

Table B: Primer sequences for spacer cloning in the pMRS reporter plasmid.

Table C: Human gRNA target sequences for EMX1.

| gRNA Name | gRNA Sequence (5′-………-3′) |
| --- | --- |
| gEMX1-1 | GAGTCCGAGCAGAAGAAGAA |
| gEMX1-2 | AGGGCTCCCATCACATCAAC |
| gEMX1-3 | CACGAAGCAGGCCAATGGGG |
| gEMX1-4 | GCGCCACCGGTTGATGTGAT |
| gEMX1-5 | GCTTCGTGGCAATGCGCCAC |
| gEMX1-6 | CTCCCCATTGGCCTGCTTCG |
| gEMX1-7 | GACATCGATGTCCTCCCCAT |
| gEMX1-14 | TCACCTCCAATGACTAGGGT |
| gEMX1-16 | GATGTCACCTCCAATGACTA |

Table D: Human gRNA target sequences for AAVS1.

| gRNA Name | gRNA Sequence (5′-………-3′) |
| --- | --- |
| gAAVS1-1 | GGATCCTGTGTCCCCGAGCT |
| gAAVS1-2 | GAGCCACATTAACCGGCCCT |
| gAAVS1-3 | GTTAATGTGGCTCTGGTTCT |
| gAAVS1-4 | GGCCCCACTGTGGGGTGGAG |
| gAAVS1-10 | TGTCCCCTCCACCCCACAGT |
| gAAVS1-11 | AGAACCAGAGCCACATTAAC |

Table E: Human gRNA target sequences for CCR5.

| gRNA Name | gRNA Sequence (5′-………-3′) |
| --- | --- |
| gCCR5-1 | CCTTCTTACTGTCCCCTTCT |
| gCCR5-2 | ATTTCCAAAGTCCCACTGGG |
| gCCR5-3 | TGTATTTCCAAAGTCCCACT |
| gCCR5-4 | TGGTTTTGTGGGCAACATGC |
| gCCR5-5 | TTTTGCAGTTTATCAGGATG |
| gCCR5-6 | TCAGCCTTTTGCAGTTTATC |
| gCCR5-7 | GTGTTCATCTTTGGTTTTGT |
| gCCR5-11 | TGACATCTACCTGCTCAACC |
| gCCR5-12 | CCCAGAAGGGGACAGTAAGA |
| gCCR5-13 | GGACAGTAAGAAGGAAAAAC |
| gCCR5-14 | CAGCATAGTGAGCCCAGAAG |
| gCCR5-15 | GCTGCCGCCCAGTGGGACTT |
| gCCR5-16 | CAATGTGTCAACTCTTGACA |

Table F: Human gRNA target sequences for HBB.

| gRNA Name | gRNA Sequence (5′-………-3′) |
| --- | --- |
| gHBB-1 | CCTGTGGGGCAAGGTGAACG |
| gHBB-2 | CCTTGATACCAACCTGCCCA |
| gHBB-3 | AAGGTGAACGTGGATGAAGT |
| gHBB-4 | GTGAACGTGGATGAAGTTGG |
| gHBB-5 | GTGAACGTGGATGAAGTTGG |
| gHBB-8 | CACGTTCACCTTGCCCCACA |
| gHBB-9 | CCCTGGGCAGGTTGGTATCA |
| gHBB-10 | CTTGCCCCACAGGGCAGTAA |
| gHBB-11 | GAAGTTGGTGGTGAGGCCCT |
| gHBB-12 | TTGGTGGTGAGGCCCTGGGC |

Table G: Primers used to amplify the U6.gRNAs of AAVS1, EMX1, HBB, and CCR5 from hgRNA plasmids for direct transfection of PCR products.

| Name | Primer Sequence | Used |
| --- | --- | --- |
| U6-EMX1gRNA-F | GGGCCCGCTCTAGAGATCCGACGCGCCATC | Amplify hgRNA by PCR |
| U6-EMX1gRNA-R | AGTTATGTAACGCGGAACTCCATATATGGG | Amplify hgRNA by PCR |
| U6-AAVS1gRNA-F | GGGCCCGCTCTAGAGATCCGACGCGCCATC |  |
| U6-AAVS1gRNA-R | AGTTATGTAACGCGGAACTCCATATATGGG |  |
| U6-HBBgRNA-F | GCGGCCGCGGGCCCGCTCTAGAGAT |  |
| U6-HBBgRNA-R | GGATCCTAGTACTCGAGAAAAAAAG |  |
| U6-CCR5gRNA-F | GCGGCCGCGGGCCCGCTCTAGAGAT |  |
| U6-CCR5gRNA-F | GGATCCTAGTACTCGAGAAAAAAAG |  |

Table H: Primers used to amplify EMX1, AAVS1, HBB, CCR5, HBD, and CCR2 from human genomic DNA for the T7EI endonuclease assay.

| gRNA Name | Primer Sequence (5′-….-3′) | Amplicon size (bp) | |
| --- | --- | --- | --- |
| EMX1-F | GAACAGGAAAACCACCCTTCTCTC | | 590 |
| EMX1-R | CAGCCAGCCCATTGCTTGTC | | 590 |
| AAVS1-F | TAGTCTTCTTCCTCCAACCCGGGCCCCT | | 301 |
| AAVS1-R | TCCAGGAAATGGGGGTGTGTCACCAGA | | 301 |
| AAVS1-2F | TTTAGCCACCTCTCCATCCTCTTG | | 842 |
| AAVS1-2R | TGGCTACTGGCCTTATCTCACAGG | | 842 |
| HBB-F | AGCCAGGGCTGGGCATAAAAG | | 298 |
| HBB-R | CCAATAGGCAGAGAGAGTCAGTG | | 298 |
| CCR5-F | GACATCAATTATTATACATCGGAGC | | 449 |
| CCR5-R | GACGCAAACACAGCCACCACCCAAG | | 449 |
| HBD-F | GTGATGGCCTGGCTCACCTGGACAACCTC | | 653 |
| HBD-R | TACATATCTCCCCACCGCATCTCTTTCAGCAG | | 653 |
| CCR2-F | TTGAACAAGGACGCATTTCCCCAG | | 361 |
| CCR2-R | CAAAGACCCACTCATTTGCAGCAG | | 361 |

Fragment sequences for the N-terminal fusion

FLAG-NLS-WT.FOKI-Linker-dCas9:

CCCTTCACCATGGATGGACTACAAAGACCATGACGGTGATTATAAAGATCATGACATCGATTACAAGGATGACGATGACAAGATGGCCCCCAAGAAGAAGAGGAAGGTGGGCATTCACCGCGGGGTACCTGGAGGTTCTATGGGATCCCAACTAGTCAAAAGTGAACTGGAGGAGAAGAAATCTGAACTTCGTCATAAATTGAAATATGTGCCTCATGAATATATTGAATTAATTGAAATTGCCAGAAATTCCACTCAGGATAGAATTCTTGAAATGAAGGTAATGGAATTTTTTATGAAAGTTTATGGATATAGAGGTAAACATTTGGGTGGATCAAGGAAACCGGACGGAGCAATTTATACTGTCGGATCTCCTATTGATTACGGTGTGATCGTGGATACTAAAGCTTATAGCGGAGGTTATAATCTGCCAATTGGCCAAGCAGATGAAATGCAACGATATGTCGAAGAAAATCAAACACGAAACAAACATATCAACCCTAATGAATGGTGGAAAGTCTATCCATCTTCTGTAACGGAATTTAAGTTTTTATTTGTGAGTGGTCACTTTAAAGGAAACTACAAAGCTCAGCTTACACGATTAAATCATATCACTAATTGTAATGGAGCTGTTCTTAGTGTAGAAGAGCTTTTAATTGGTGGAGAAATGATTAAAGCCGGCACATTAACCTTAGAGGAAGTCAGACGGAAATTTAATAACGGCGAGATAAACTTTAGCGGCAGCGAGACTCCCGGGACCTCAGAGTCCGCCACACCCGAAACCCCATGGCCCTTCA

FLAG-NLS-Sharky.FOKI-Linker-dCas9:

CCCTTCACCATGGATGGACTACAAAGACCATGACGGTGATTATAAAGATCATGACATCGATTACAAGGATGACGATGACAAGATGGCCCCCAAGAAGAAGAGGAAGGTGGGCATTCACCGCGGGGTACCTGGAGGTTCTATGGGATCCCAACTAGTCAAAAGTGAACTGGAGGAGAAGAAATCTGAACTTCGTCATAAATTGAAATATGTGCCTCATGAATATATTGAATTAATTGAAATTGCCAGAAATCCCACTCAGGATAGAATTCTTGAAATGAAGGTAATGGAATTTTTTATGAAAGTTTATGGATATAGAGGTGAACATTTGGGTGGATCAAGGAAACCGGACGGAGCAATTTATACTGTCGGATCTCCTATTGATTACGGTGTGATCGTGGATACTAAAGCTTATAGCGGAGGTTATAATCTGCCAATTGGCCAAGCAGATGAAATGCAACGATATGTCGAAGAAAATCAAACACGAAACAAACATATCAACCCTAATGAATGGTGGAAAGTCTATCCATCTTCTGTAACGGAATTTAAGTTTTTATTTGTGAGTGGTCACTTTAAAGGAAACTACAAAGCTCAGCTTACACGATTAAATCATATCACTAATTGTAATGGAGCTGTTCTTAGTGTAGAAGAGCTTTTAATTGGTGGAGAAATGATTAAAGCCGGCACATTAACCTTAGAGGAAGTGAGACGGAAATTTAATAACGGCGAGATAAACTTT

1. Spacers with PAM-in orientation cloned in pMRS plasmid

pMRS-gRNA PAM-out (F and R): 5′-GAGTCCGAGCAGAAGAAGAA-3′

Spacer 2:


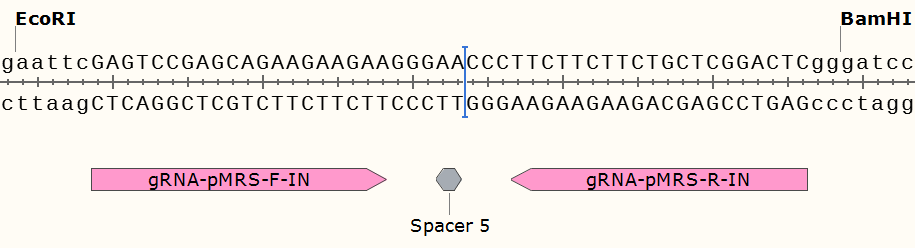


Spacer 5:


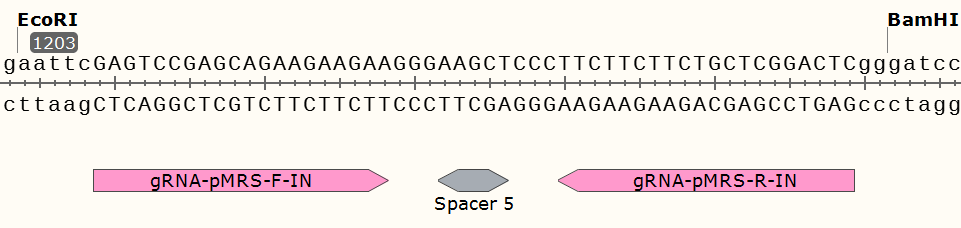


Spacer 8:


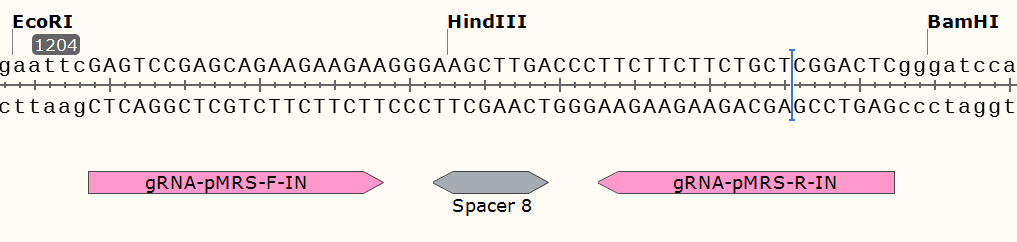


Spacer 11:


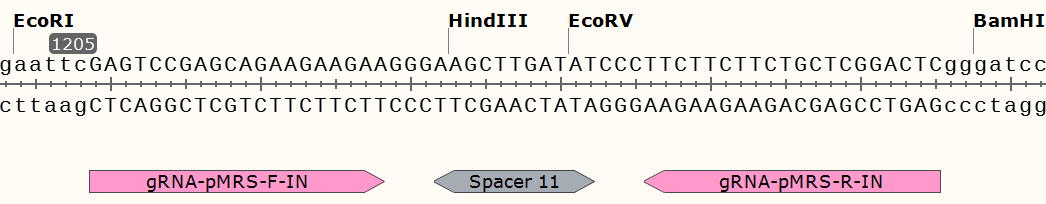


Spacer 14:


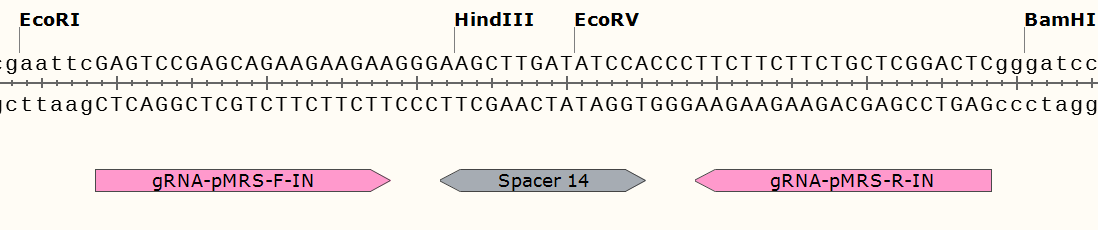


Spacer 17:


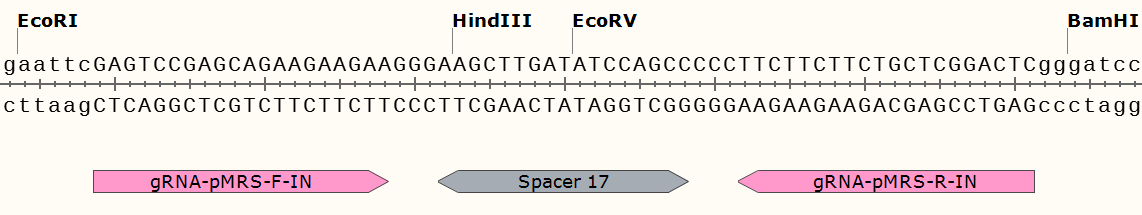


Spacer 20:


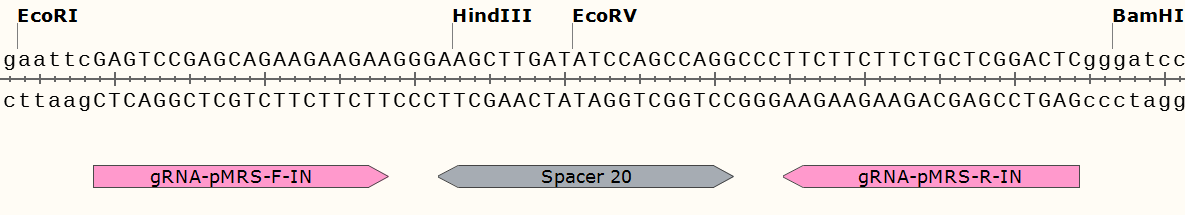


Spacer 23:


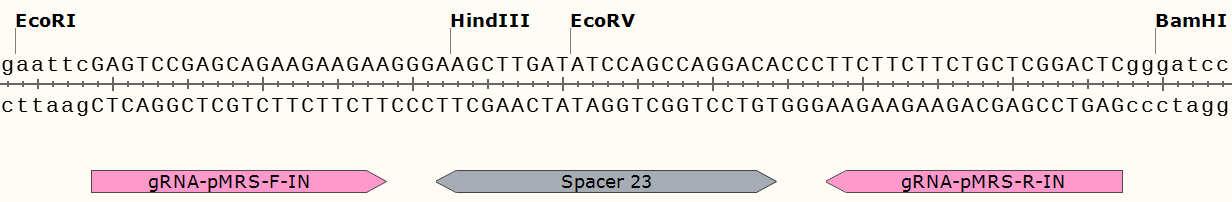


Spacer 26:


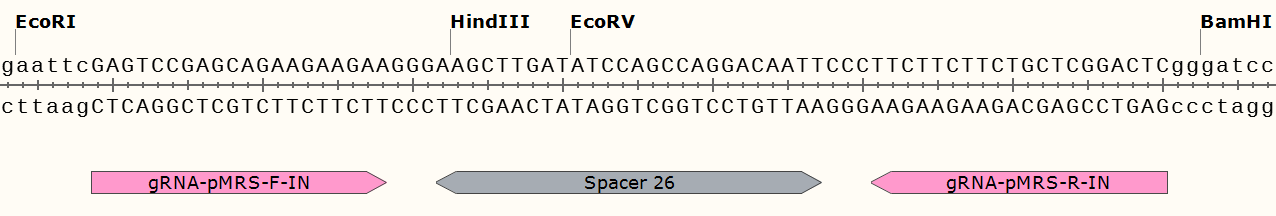


Spacer 29


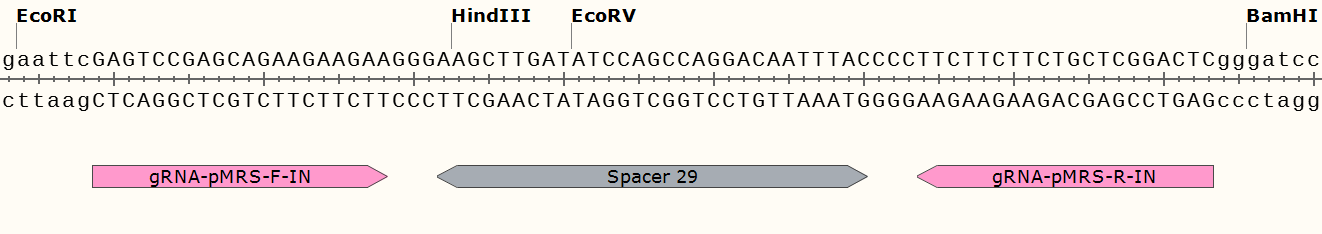


Spacer 32:


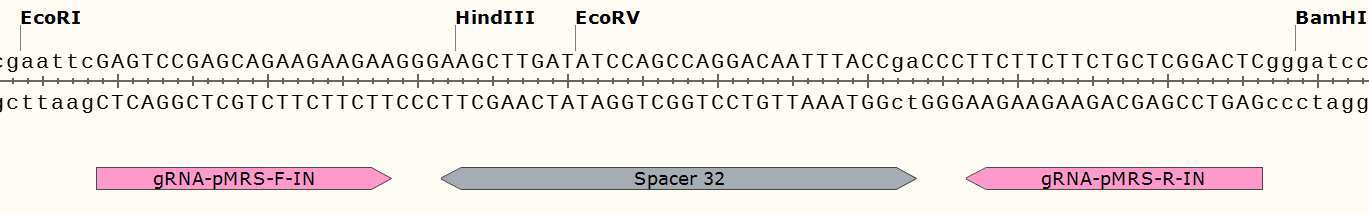


Spacer 35:


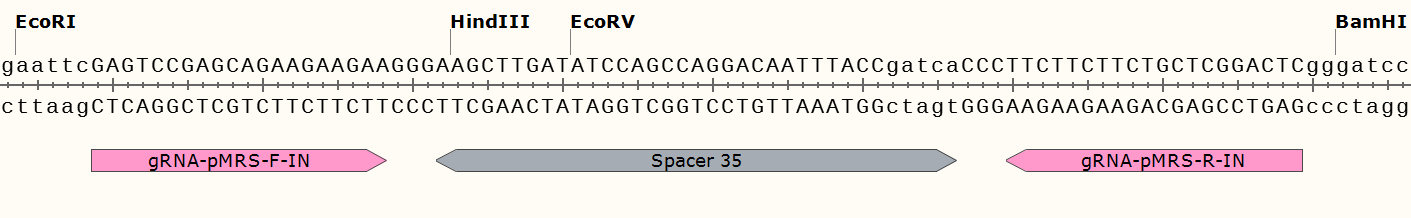


Spacer 38:


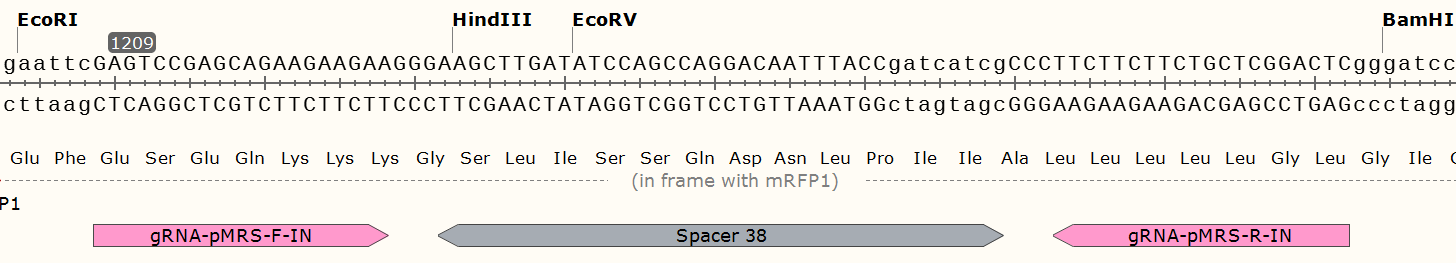


1. Spacers with PAM-out orientation cloned in pMRS plasmid

pMRS-gRNA PAM-out (F and R): 5′-CCATGAGGCCATCGCACTAG-3′

Spacer 3:


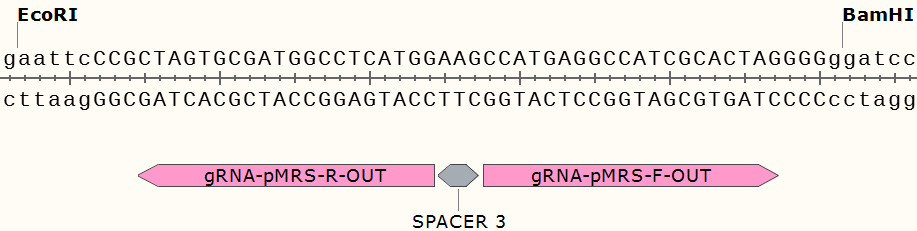


Spacer 6:


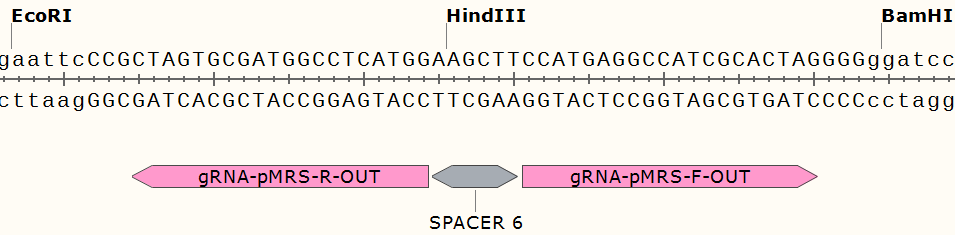


Spacer 9:


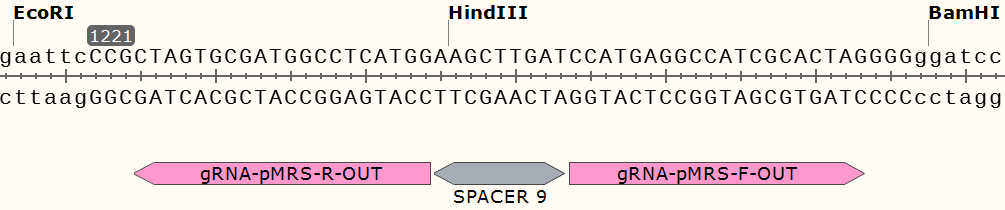


Spacer 12:


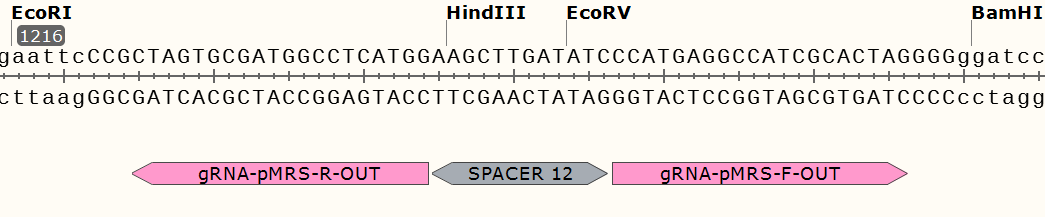


Spacer 15:


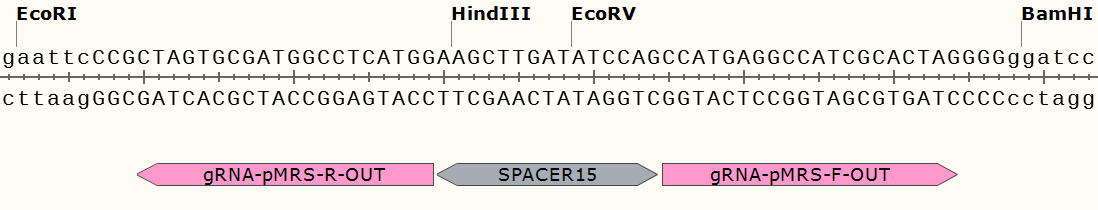


Spacer 18:


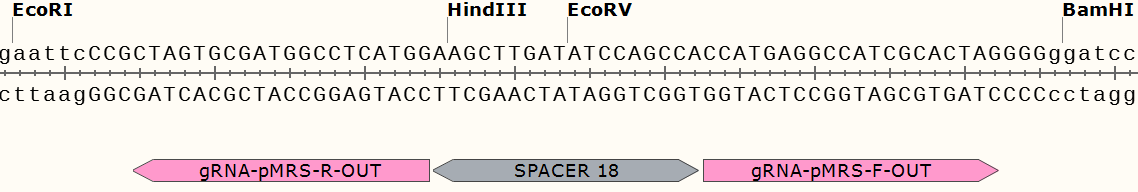


Spacer 21:


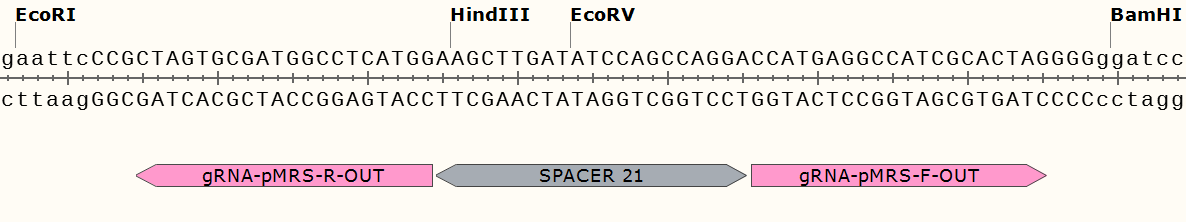


Spacer 24:


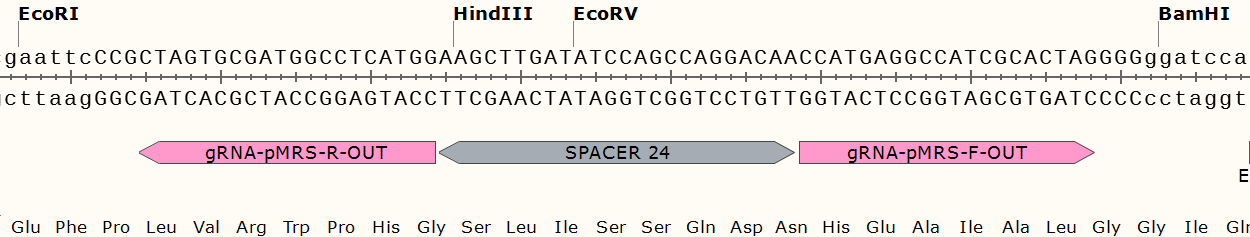


Spacer 27:


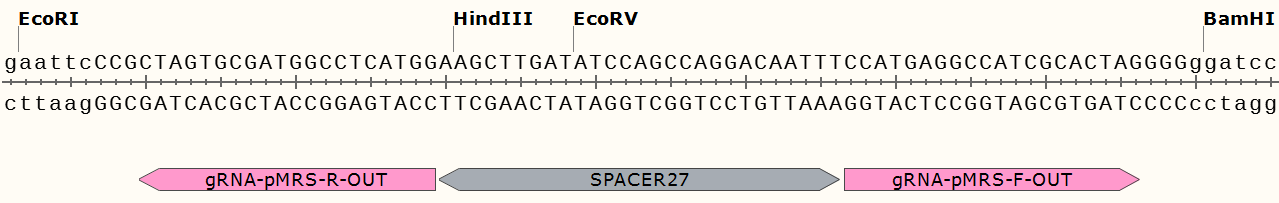
Spacer 30:


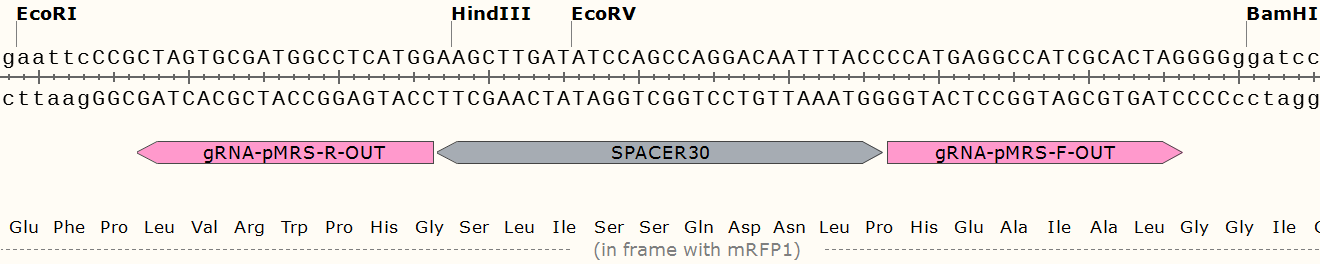


Spacer 33:


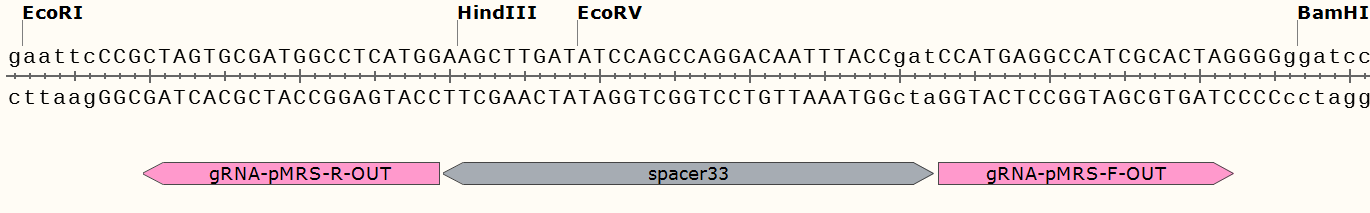


Spacer 36:


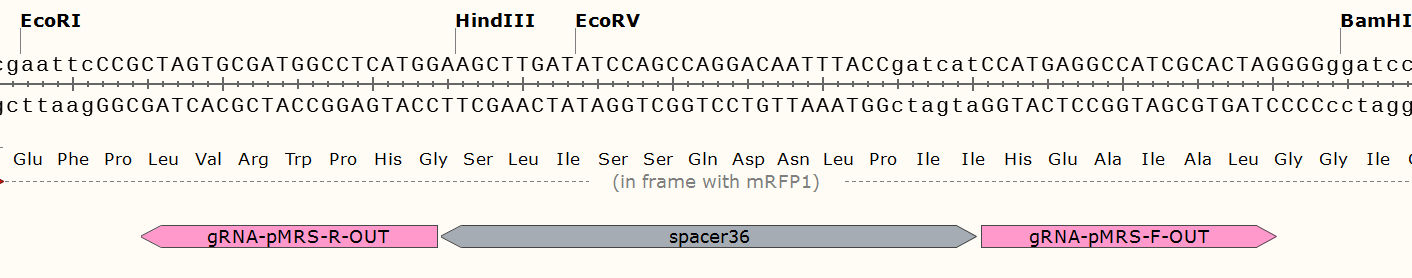


Spacer 39:


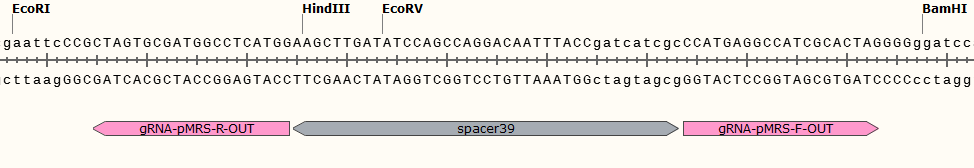


pMRS reporter plasmid used to clone the spacers with targets in the in and out orientations to test the activity of the C- and N-terminus dCas9 and FokI fusions.


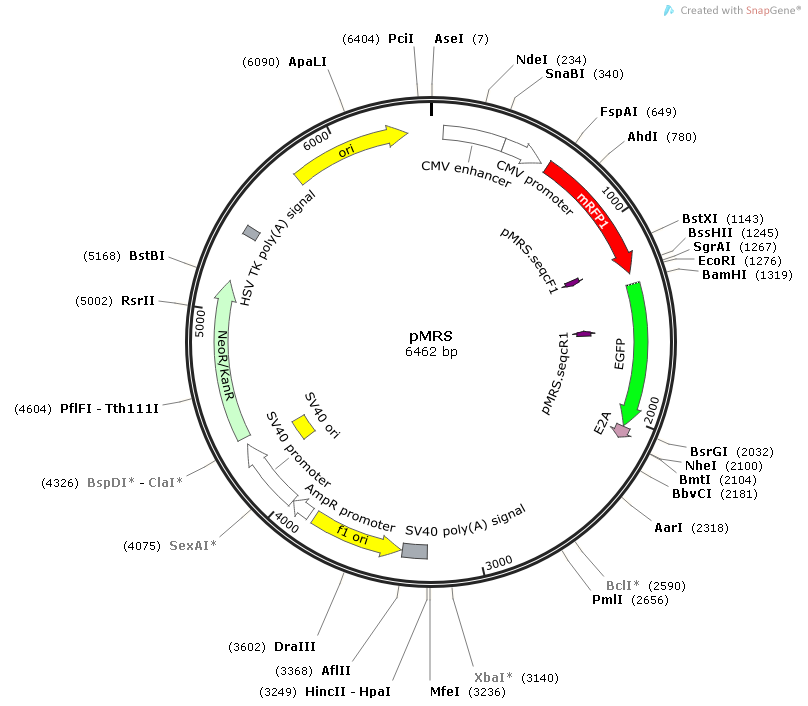


The pMRS reporter system and spacer size ranging from 3–39 bp.

1. EMX1 target sequence:

GAACAGGAAAACCACCCTTCTCTCTGGCCCACTGTGTCCTCTTCCTGCCCTGCCATCCCCTTCTGTGAATGTTAGACCCATGGGAGCAGCTGGTCAGAGGGGACCCCGGCCTGGGGCCCCTAACCCTATGTAGCCTCAGTCTTCCCATCAGGCTCTCAGCTCAGCCTGAGTGTTGAGGCCCCAGTGGCTGCTCTGGGGGCCTCCTGAGTTTCTCATCTGTGCCCCTCCCTCCCTGGCCCAGGTGAAGGTGTGGTTCCAGAACCGGAGGACAAAGTACAAACGGCAGAAGCTGGAGGAGGAAGGGCCTGAGTCCGAGCAGAAGAAGAAGGGCTCCCATCACATCAACCGGTGGCGCATTGCCACGAAGCAGGCCAATGGGGAGGACATCGATGTCACCTCCAATGACTAGGGTGGGCAACCACAAACCCACGAGGGCAGAGTGCTGCTTGCTGCTGGCCAGGCCCCTGCGTGGGCCCAAGCTGGACTCTGGCCACTCCCTGGCCAGGCTTTGGGGAGGCCTGGAGTCATGGCCCCACAGGGCTTGAAGCCCGGGGCCGCCATTGACAGAGGGACAAGCAATGGGCTGGCTG

1. Different target sequences with PAM-in and PAM-out orientations.


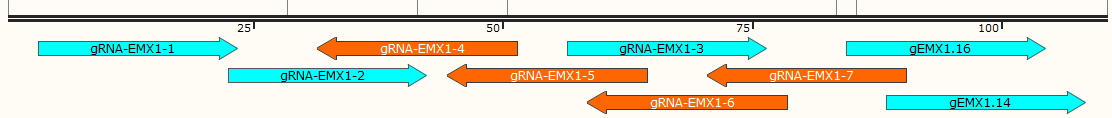


1. Possible combinations of pairs of EMX1 gRNAs in PAM-in and PAM-out orientations:


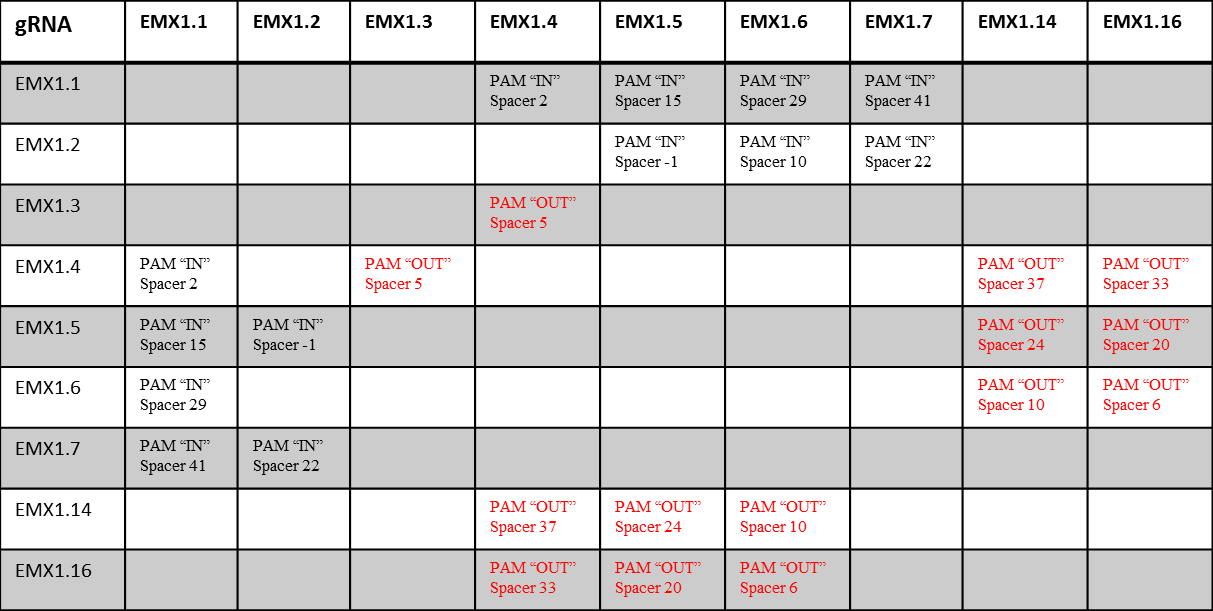


1. AAVS1 target sequence

TAGTCTTCTTCCTCCAACCCGGGCCCCTATGTCCACTTCAGGACAGCATGTTTGCTGCCTCCAGGGATCCTGTGTCCCCGAGCTGGGACCACCTTATATTCCCAGGGCCGGTTAATGTGGCTCTGGTTCTGGGTACTTTTATCTGTCCCCTCCACCCCACAGTGGGGCCACTAGGGACAGGATTGGTGACAGAAAAGCCCCATCCTTAGGCCTCCTCCTTCCTAGTCTCCTGATATTGGGTCTAACCCCCACCTCCTGTTAGGCAGATTCCTTATCTGGTGACACACCCCCATTTCCTGG

1. Different targets sequences with PAM-in and PAM-out orientations.


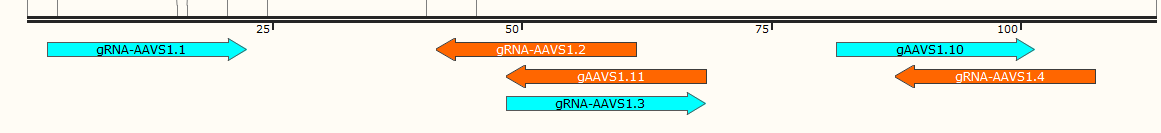


1. Possible combination of a pair of AAVS1 gRNAs in PAM -in and PAM-out from the spacer:


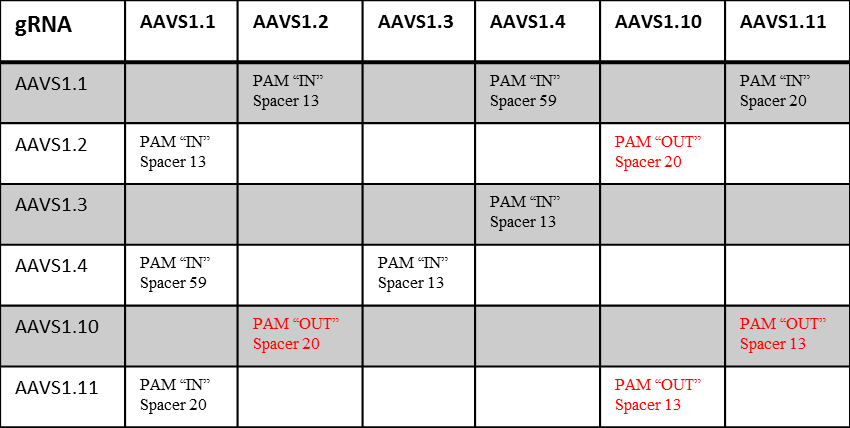


1. CCR5 target sequence

GACATCAATTATTATACATCGGAGCCCTGCCAAAAAATCAATGTGAAGCAAATCGCAGCCCGCCTCCTGCCTCCGCTCTACTCACTGGTGTTCATCTTTGGTTTTGTGGGCAACATGCTGGTCATCCTCATCCTGATAAACTGCAAAAGGCTGAAGAGCATGACTGACATCTACCTGCTCAACCTGGCCATCTCTGACCTGTTTTTCCTTCTTACTGTCCCCTTCTGGGCTCACTATGCTGCCGCCCAGTGGGACTTTGGAAATACAATGTGTCAACTCTTGACAGGGCTCTATTTTATAGGCTTCTTCTCTGGAATCTTCTTCATCATCCTCCTGACAATCGATAGGTACCTGGCTGTCGTCCATGCTGTGTTTGCTTTAAAAGCCAGGACGGTCACCTTTGGGGTGGTGACAAGTGTGATCACTTGGGTGGTGGCTGTGTTTGC

1. Different targets sequences with PAM-in and PAM-out orientations.


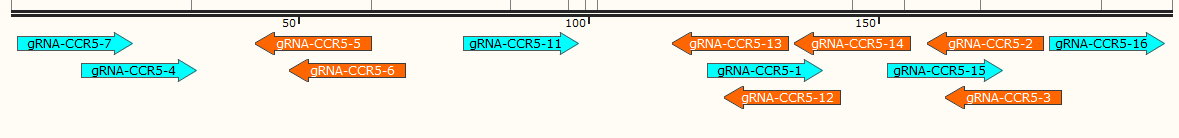


1. Possible combinations of pairs of EMX1 gRNAs in PAM-in and PAM-out orientations:


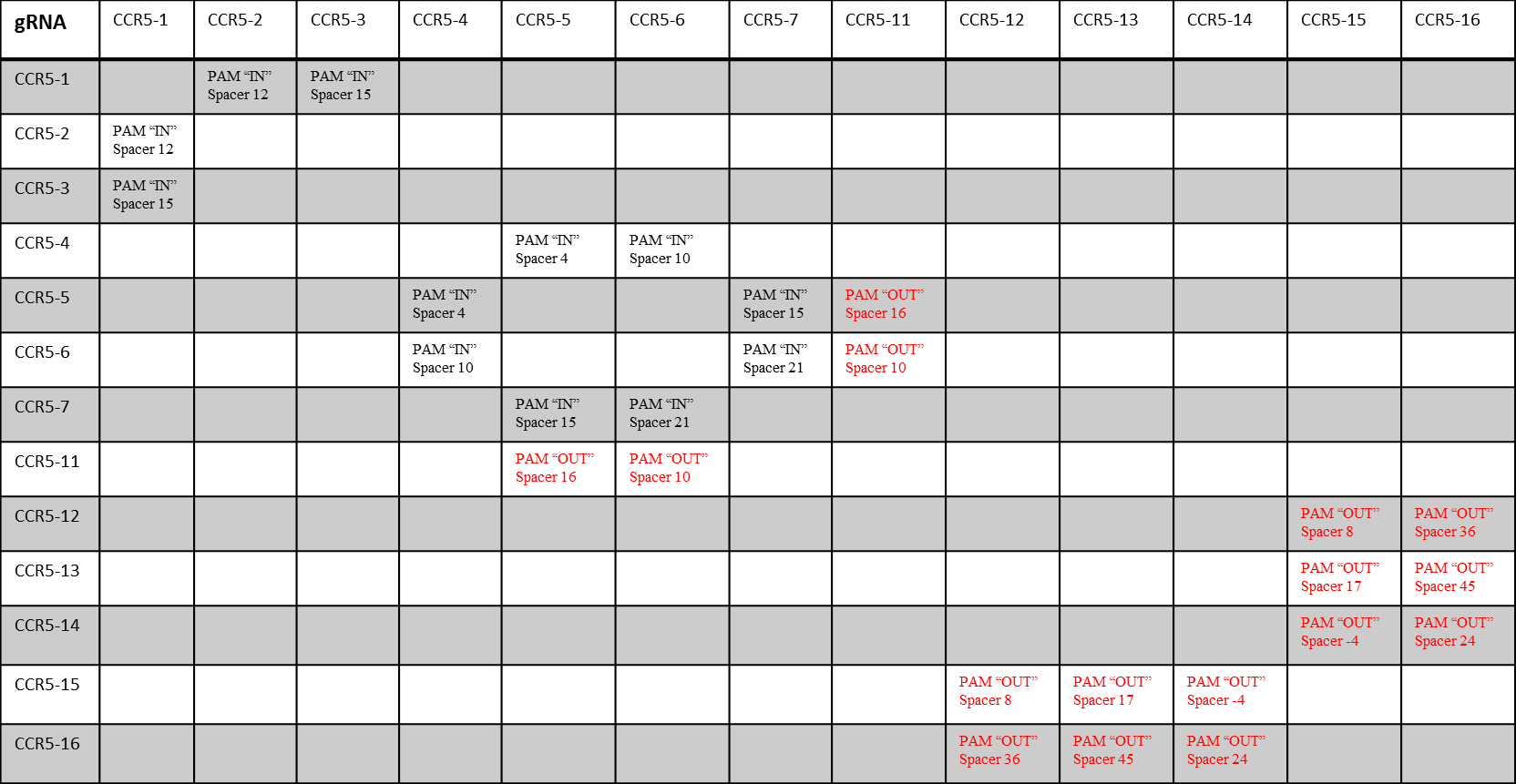


1. HBB target sequence:

AGCCAGGGCTGGGCATAAAAGTCAGGGCAGAGCCATCTATTGCTTACATTTGCTTCTGACACAACTGTGTTCACTAGCAACCTCAAACAGACACCATGGTGCACCTGACTCCTGAGGAGAAGTCTGCCGTTACTGCCCTGTGGGGCAAGGTGAACGTGGATGAAGTTGGTGGTGAGGCCCTGGGCAGGTTGGTATCAAGGTTACAAGACAGGTTTAAGGAGACCAATAGAAACTGGGCATGTGGAGACAGAGAAGACTCTTGGGTTTCTGATAGGCACTGACTCTCTCTGCCTATTGG

1. Different target sequences with PAM-in and PAM-out orientations.


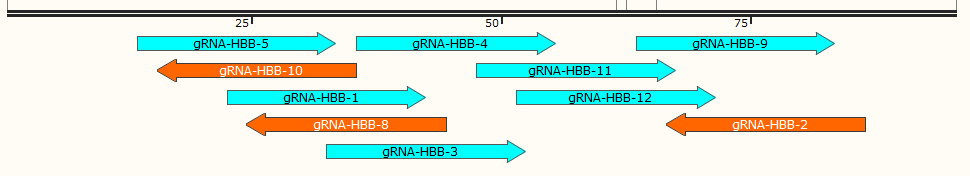


1. Possible combinations of pairs of AAVS1 gRNAs in PAM-in and PAM-out orientations and spacer distances:


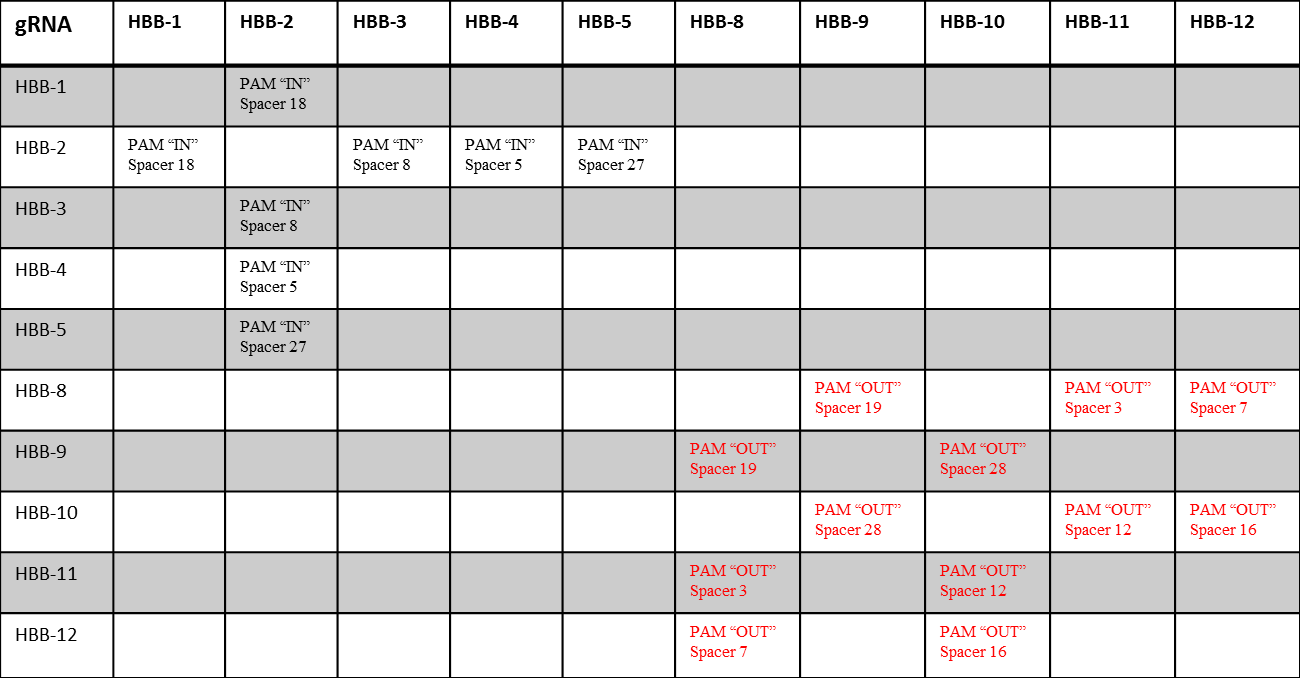


Human gRNA under the U6 promoter:

GAGGGCCTATTTCCCATGATTCCTTCATATTTGCATATACGATACAAGGCTGTTAGAGAGATAATTAGAATTAATTTGACTGTAAACACAAAGATATTAGTACAAAATACGTGACGTAGAAAGTAATAATTTCTTGGGTAGTTTGCAGTTTTAAAATTATGTTTTAAAATGGACTATCATATGCTTACCGTAACTTGAAAGTATTTCGATTTCTTGGGTTTATATATCTTGTGGAAAGGACgNNNNNNNNNNNNNNNNNNNNGTTTTAGAGCTAGAAATAGCAAGTTAAAATAAGGCTAGTCCGTTATCAACTTGAAAAAGTGGCACCGAGTCGGTGCTTTTTTT

(U6 promoter sequence is highlighted in red; guide sequence is highlighted in green, the gRNA scaffold in purple, and the terminator fragment is highlighted in black)

Construct DNA and amino acid sequences used in this study.

1. dCas9
   1. DNA sequence:

ATGGACAAGAAGTATTCTATCGGACTGGCCATCGGGACTAATAGCGTCGGGTGGGCCGTGATCACTGACGAGTACAAGGTGCCCTCTAAGAAGTTCAAGGTGCTCGGGAACACCGACCGGCATTCCATCAAGAAAAATCTGATCGGAGCTCTCCTCTTTGATTCAGGGGAGACCGCTGAAGCAACCCGCCTCAAGCGGACTGCTAGACGGCGGTACACCAGGAGGAAGAACCGGATTTGTTACCTTCAAGAGATATTCTCCAACGAAATGGCAAAGGTCGACGACAGCTTCTTCCATAGGCTGGAAGAATCATTCCTCGTGGAAGAGGATAAGAAGCATGAACGGCATCCCATCTTCGGTAATATCGTCGACGAGGTGGCCTATCACGAGAAATACCCAACCATCTACCATCTTCGCAAAAAGCTGGTGGACTCAACCGACAAGGCAGACCTCCGGCTTATCTACCTGGCCCTGGCCCACATGATCAAGTTCAGAGGCCACTTCCTGATCGAGGGCGACCTCAATCCTGACAATAGCGATGTGGATAAACTGTTCATCCAGCTGGTGCAGACTTACAACCAGCTCTTTGAAGAGAACCCCATCAATGCAAGCGGAGTCGATGCCAAGGCCATTCTGTCAGCCCGGCTGTCAAAGAGCCGCAGACTTGAGAATCTTATCGCTCAGCTGCCGGGTGAAAAGAAAAATGGACTGTTCGGGAACCTGATTGCTCTTTCACTTGGGCTGACTCCCAATTTCAAGTCTAATTTCGACCTGGCAGAGGATGCCAAGCTGCAACTGTCCAAGGACACCTATGATGACGATCTCGACAACCTCCTGGCCCAGATCGGTGACCAATACGCCGACCTTTTCCTTGCTGCTAAGAATCTTTCTGACGCCATCCTGCTGTCTGACATTCTCCGCGTGAACACTGAAATCACCAAGGCCCCTCTTTCAGCTTCAATGATTAAGCGGTATGATGAGCACCACCAGGACCTGACCCTGCTTAAGGCACTCGTCCGGCAGCAGCTTCCGGAGAAGTACAAGGAAATCTTCTTTGACCAGTCAAAGAATGGATACGCCGGCTACATCGACGGAGGTGCCTCCCAAGAGGAATTTTATAAGTTTATCAAACCTATCCTTGAGAAGATGGACGGCACCGAAGAGCTCCTCGTGAAACTGAATCGGGAGGATCTGCTGCGGAAGCAGCGCACTTTCGACAATGGGAGCATTCCCCACCAGATCCATCTTGGGGAGCTTCACGCCATCCTTCGGCGCCAAGAGGACTTCTACCCCTTTCTTAAGGACAACAGGGAGAAGATTGAGAAAATTCTCACTTTCCGCATCCCCTACTACGTGGGACCCCTCGCCAGAGGAAATAGCCGGTTTGCTTGGATGACCAGAAAGTCAGAAGAAACTATCACTCCCTGGAACTTCGAAGAGGTGGTGGACAAGGGAGCCAGCGCTCAGTCATTCATCGAACGGATGACTAACTTCGATAAGAACCTCCCCAATGAGAAGGTCCTGCCGAAACATTCCCTGCTCTACGAGTACTTTACCGTGTACAACGAGCTGACCAAGGTGAAATATGTCACCGAAGGGATGAGGAAGCCCGCATTCCTGTCAGGCGAACAAAAGAAGGCAATTGTGGACCTTCTGTTCAAGACCAATAGAAAGGTGACCGTGAAGCAGCTGAAGGAGGACTATTTCAAGAAAATTGAATGCTTCGACTCTGTGGAGATTAGCGGGGTCGAAGATCGGTTCAACGCAAGCCTGGGTACCTACCATGATCTGCTTAAGATCATCAAGGACAAGGATTTTCTGGACAATGAGGAGAACGAGGACATCCTTGAGGACATTGTCCTGACTCTCACTCTGTTCGAGGACCGGGAAATGATCGAGGAGAGGCTTAAGACCTACGCCCATCTGTTCGACGATAAAGTGATGAAGCAACTTAAACGGAGAAGATATACCGGATGGGGACGCCTTAGCCGCAAACTCATCAACGGAATCCGGGACAAACAGAGCGGAAAGACCATTCTTGATTTCCTTAAGAGCGACGGATTCGCTAATCGCAACTTCATGCAACTTATCCATGATGATTCCCTGACCTTTAAGGAGGACATCCAGAAGGCCCAAGTGTCTGGACAAGGTGACTCACTGCACGAGCATATCGCAAATCTGGCTGGTTCACCCGCTATTAAGAAGGGTATTCTCCAGACCGTGAAAGTCGTGGACGAGCTGGTCAAGGTGATGGGTCGCCATAAACCAGAGAACATTGTCATCGAGATGGCCAGGGAAAACCAGACTACCCAGAAGGGACAGAAGAACAGCAGGGAGCGGATGAAAAGAATTGAGGAAGGGATTAAGGAGCTCGGGTCACAGATCCTTAAAGAGCACCCGGTGGAAAACACCCAGCTTCAGAATGAGAAGCTCTATCTGTACTACCTTCAAAATGGACGCGATATGTATGTGGACCAAGAGCTTGATATCAACAGGCTCTCAGACTACGACGTGGACGCCATCGTCCCTCAGAGCTTCCTCAAAGACGACTCAATTGACAATAAGGTGCTGACTCGCTCAGACAAGAACCGGGGAAAGTCAGATAACGTGCCCTCAGAGGAAGTCGTGAAAAAGATGAAGAACTATTGGCGCCAGCTTCTGAACGCAAAGCTGATCACTCAGCGGAAGTTCGACAATCTCACTAAGGCTGAGAGGGGCGGACTGAGCGAACTGGACAAAGCAGGATTCATTAAACGGCAACTTGTGGAGACTCGGCAGATTACTAAACATGTCGCCCAAATCCTTGACTCACGCATGAATACCAAGTACGACGAAAACGACAAACTTATCCGCGAGGTGAAGGTGATTACCCTGAAGTCCAAGCTGGTCAGCGATTTCAGAAAGGACTTTCAATTCTACAAAGTGCGGGAGATCAATAACTATCATCATGCTCATGACGCATATCTGAATGCCGTGGTGGGAACCGCCCTGATCAAGAAGTACCCAAAGCTGGAAAGCGAGTTCGTGTACGGAGACTACAAGGTCTACGACGTGCGCAAGATGATTGCCAAATCTGAGCAGGAGATCGGAAAGGCCACCGCAAAGTACTTCTTCTACAGCAACATCATGAATTTCTTCAAGACCGAAATCACCCTTGCAAACGGTGAGATCCGGAAGAGGCCGCTCATCGAGACTAATGGGGAGACTGGCGAAATCGTGTGGGACAAGGGCAGAGATTTCGCTACCGTGCGCAAAGTGCTTTCTATGCCTCAAGTGAACATCGTGAAGAAAACCGAGGTGCAAACCGGAGGCTTTTCTAAGGAATCAATCCTCCCCAAGCGCAACTCCGACAAGCTCATTGCAAGGAAGAAGGATTGGGACCCTAAGAAGTACGGCGGATTCGATTCACCAACTGTGGCTTATTCTGTCCTGGTCGTGGCTAAGGTGGAAAAAGGAAAGTCTAAGAAGCTCAAGAGCGTGAAGGAACTGCTGGGTATCACCATTATGGAGCGCAGCTCCTTCGAGAAGAACCCAATTGACTTTCTCGAAGCCAAAGGTTACAAGGAAGTCAAGAAGGACCTTATCATCAAGCTCCCAAAGTATAGCCTGTTCGAACTGGAGAATGGGCGGAAGCGGATGCTCGCCTCCGCTGGCGAACTTCAGAAGGGTAATGAGCTGGCTCTCCCCTCCAAGTACGTGAATTTCCTCTACCTTGCAAGCCATTACGAGAAGCTGAAGGGGAGCCCCGAGGACAACGAGCAAAAGCAACTGTTTGTGGAGCAGCATAAGCATTATCTGGACGAGATCATTGAGCAGATTTCCGAGTTTTCTAAACGCGTCATTCTCGCTGATGCCAACCTCGATAAAGTCCTTAGCGCATACAATAAGCACAGAGACAAACCAATTCGGGAGCAGGCTGAGAATATCATCCACCTGTTCACCCTCACCAATCTTGGTGCCCCTGCCGCATTCAAGTACTTCGACACCACCATCGACCGGAAACGCTATACCTCCACCAAAGAAGTGCTGGACGCCACCCTCATCCACCAGAGCATCACCGGACTTTACGAAACTCGGATTGACCTCTCACAGCTCGGAGGGGATGAGGGAGCTGATCCAAAAAAGAAGAGAAAGGTAGATCCAAAAAAGAAGAGAAAGGTAGATCCAAAAAAGAAGAGAAAGGTATAG

- 1. dCas9 amino acid sequence:

MDKKYSIGLAIGTNSVGWAVITDEYKVPSKKFKVLGNTDRHSIKKNLIGALLFDSGETAEATRLKRTARRRYTRRKNRICYLQEIFSNEMAKVDDSFFHRLEESFLVEEDKKHERHPIFGNIVDEVAYHEKYPTIYHLRKKLVDSTDKADLRLIYLALAHMIKFRGHFLIEGDLNPDNSDVDKLFIQLVQTYNQLFEENPINASGVDAKAILSARLSKSRRLENLIAQLPGEKKNGLFGNLIALSLGLTPNFKSNFDLAEDAKLQLSKDTYDDDLDNLLAQIGDQYADLFLAAKNLSDAILLSDILRVNTEITKAPLSASMIKRYDEHHQDLTLLKALVRQQLPEKYKEIFFDQSKNGYAGYIDGGASQEEFYKFIKPILEKMDGTEELLVKLNREDLLRKQRTFDNGSIPHQIHLGELHAILRRQEDFYPFLKDNREKIEKILTFRIPYYVGPLARGNSRFAWMTRKSEETITPWNFEEVVDKGASAQSFIERMTNFDKNLPNEKVLPKHSLLYEYFTVYNELTKVKYVTEGMRKPAFLSGEQKKAIVDLLFKTNRKVTVKQLKEDYFKKIECFDSVEISGVEDRFNASLGTYHDLLKIIKDKDFLDNEENEDILEDIVLTLTLFEDREMIEERLKTYAHLFDDKVMKQLKRRRYTGWGRLSRKLINGIRDKQSGKTILDFLKSDGFANRNFMQLIHDDSLTFKEDIQKAQVSGQGDSLHEHIANLAGSPAIKKGILQTVKVVDELVKVMGRHKPENIVIEMARENQTTQKGQKNSRERMKRIEEGIKELGSQILKEHPVENTQLQNEKLYLYYLQNGRDMYVDQELDINRLSDYDVDAIVPQSFLKDDSIDNKVLTRSDKNRGKSDNVPSEEVVKKMKNYWRQLLNAKLITQRKFDNLTKAERGGLSELDKAGFIKRQLVETRQITKHVAQILDSRMNTKYDENDKLIREVKVITLKSKLVSDFRKDFQFYKVREINNYHHAHDAYLNAVVGTALIKKYPKLESEFVYGDYKVYDVRKMIAKSEQEIGKATAKYFFYSNIMNFFKTEITLANGEIRKRPLIETNGETGEIVWDKGRDFATVRKVLSMPQVNIVKKTEVQTGGFSKESILPKRNSDKLIARKKDWDPKKYGGFDSPTVAYSVLVVAKVEKGKSKKLKSVKELLGITIMERSSFEKNPIDFLEAKGYKEVKKDLIIKLPKYSLFELENGRKRMLASAGELQKGNELALPSKYVNFLYLASHYEKLKGSPEDNEQKQLFVEQHKHYLDEIIEQISEFSKRVILADANLDKVLSAYNKHRDKPIREQAENIIHLFTLTNLGAPAAFKYFDTTIDRKRYTSTKEVLDATLIHQSITGLYETRIDLSQLGGDEGADPKKKRKVDPKKKRKVDPKKKRKV

1. fdCas9 DNA and amino acid sequence
   1. DNA sequence

ATGGACTACAAAGACCATGACGGTGATTATAAAGATCATGACATCGATTACAAGGATGACGATGACAAGATGGCCCCCAAGAAGAAGAGGAAGGTGGGCATTCACCGCGGGGTACCTGGAGGTTCTATGGGATCCCAACTAGTCAAAAGTGAACTGGAGGAGAAGAAATCTGAACTTCGTCATAAATTGAAATATGTGCCTCATGAATATATTGAATTAATTGAAATTGCCAGAAATTCCACTCAGGATAGAATTCTTGAAATGAAGGTAATGGAATTTTTTATGAAAGTTTATGGATATAGAGGTAAACATTTGGGTGGATCAAGGAAACCGGACGGAGCAATTTATACTGTCGGATCTCCTATTGATTACGGTGTGATCGTGGATACTAAAGCTTATAGCGGAGGTTATAATCTGCCAATTGGCCAAGCAGATGAAATGCAACGATATGTCGAAGAAAATCAAACACGAAACAAACATATCAACCCTAATGAATGGTGGAAAGTCTATCCATCTTCTGTAACGGAATTTAAGTTTTTATTTGTGAGTGGTCACTTTAAAGGAAACTACAAAGCTCAGCTTACACGATTAAATCATATCACTAATTGTAATGGAGCTGTTCTTAGTGTAGAAGAGCTTTTAATTGGTGGAGAAATGATTAAAGCCGGCACATTAACCTTAGAGGAAGTCAGACGGAAATTTAATAACGGCGAGATAAACTTTAGCGGCAGCGAGACTCCCGGGACCTCAGAGTCCGCCACACCCGAAACCATGGACAAGAAGTATTCTATCGGACTGGCCATCGGGACTAATAGCGTCGGGTGGGCCGTGATCACTGACGAGTACAAGGTGCCCTCTAAGAAGTTCAAGGTGCTCGGGAACACCGACCGGCATTCCATCAAGAAAAATCTGATCGGAGCTCTCCTCTTTGATTCAGGGGAGACCGCTGAAGCAACCCGCCTCAAGCGGACTGCTAGACGGCGGTACACCAGGAGGAAGAACCGGATTTGTTACCTTCAAGAGATATTCTCCAACGAAATGGCAAAGGTCGACGACAGCTTCTTCCATAGGCTGGAAGAATCATTCCTCGTGGAAGAGGATAAGAAGCATGAACGGCATCCCATCTTCGGTAATATCGTCGACGAGGTGGCCTATCACGAGAAATACCCAACCATCTACCATCTTCGCAAAAAGCTGGTGGACTCAACCGACAAGGCAGACCTCCGGCTTATCTACCTGGCCCTGGCCCACATGATCAAGTTCAGAGGCCACTTCCTGATCGAGGGCGACCTCAATCCTGACAATAGCGATGTGGATAAACTGTTCATCCAGCTGGTGCAGACTTACAACCAGCTCTTTGAAGAGAACCCCATCAATGCAAGCGGAGTCGATGCCAAGGCCATTCTGTCAGCCCGGCTGTCAAAGAGCCGCAGACTTGAGAATCTTATCGCTCAGCTGCCGGGTGAAAAGAAAAATGGACTGTTCGGGAACCTGATTGCTCTTTCACTTGGGCTGACTCCCAATTTCAAGTCTAATTTCGACCTGGCAGAGGATGCCAAGCTGCAACTGTCCAAGGACACCTATGATGACGATCTCGACAACCTCCTGGCCCAGATCGGTGACCAATACGCCGACCTTTTCCTTGCTGCTAAGAATCTTTCTGACGCCATCCTGCTGTCTGACATTCTCCGCGTGAACACTGAAATCACCAAGGCCCCTCTTTCAGCTTCAATGATTAAGCGGTATGATGAGCACCACCAGGACCTGACCCTGCTTAAGGCACTCGTCCGGCAGCAGCTTCCGGAGAAGTACAAGGAAATCTTCTTTGACCAGTCAAAGAATGGATACGCCGGCTACATCGACGGAGGTGCCTCCCAAGAGGAATTTTATAAGTTTATCAAACCTATCCTTGAGAAGATGGACGGCACCGAAGAGCTCCTCGTGAAACTGAATCGGGAGGATCTGCTGCGGAAGCAGCGCACTTTCGACAATGGGAGCATTCCCCACCAGATCCATCTTGGGGAGCTTCACGCCATCCTTCGGCGCCAAGAGGACTTCTACCCCTTTCTTAAGGACAACAGGGAGAAGATTGAGAAAATTCTCACTTTCCGCATCCCCTACTACGTGGGACCCCTCGCCAGAGGAAATAGCCGGTTTGCTTGGATGACCAGAAAGTCAGAAGAAACTATCACTCCCTGGAACTTCGAAGAGGTGGTGGACAAGGGAGCCAGCGCTCAGTCATTCATCGAACGGATGACTAACTTCGATAAGAACCTCCCCAATGAGAAGGTCCTGCCGAAACATTCCCTGCTCTACGAGTACTTTACCGTGTACAACGAGCTGACCAAGGTGAAATATGTCACCGAAGGGATGAGGAAGCCCGCATTCCTGTCAGGCGAACAAAAGAAGGCAATTGTGGACCTTCTGTTCAAGACCAATAGAAAGGTGACCGTGAAGCAGCTGAAGGAGGACTATTTCAAGAAAATTGAATGCTTCGACTCTGTGGAGATTAGCGGGGTCGAAGATCGGTTCAACGCAAGCCTGGGTACCTACCATGATCTGCTTAAGATCATCAAGGACAAGGATTTTCTGGACAATGAGGAGAACGAGGACATCCTTGAGGACATTGTCCTGACTCTCACTCTGTTCGAGGACCGGGAAATGATCGAGGAGAGGCTTAAGACCTACGCCCATCTGTTCGACGATAAAGTGATGAAGCAACTTAAACGGAGAAGATATACCGGATGGGGACGCCTTAGCCGCAAACTCATCAACGGAATCCGGGACAAACAGAGCGGAAAGACCATTCTTGATTTCCTTAAGAGCGACGGATTCGCTAATCGCAACTTCATGCAACTTATCCATGATGATTCCCTGACCTTTAAGGAGGACATCCAGAAGGCCCAAGTGTCTGGACAAGGTGACTCACTGCACGAGCATATCGCAAATCTGGCTGGTTCACCCGCTATTAAGAAGGGTATTCTCCAGACCGTGAAAGTCGTGGACGAGCTGGTCAAGGTGATGGGTCGCCATAAACCAGAGAACATTGTCATCGAGATGGCCAGGGAAAACCAGACTACCCAGAAGGGACAGAAGAACAGCAGGGAGCGGATGAAAAGAATTGAGGAAGGGATTAAGGAGCTCGGGTCACAGATCCTTAAAGAGCACCCGGTGGAAAACACCCAGCTTCAGAATGAGAAGCTCTATCTGTACTACCTTCAAAATGGACGCGATATGTATGTGGACCAAGAGCTTGATATCAACAGGCTCTCAGACTACGACGTGGACGCCATCGTCCCTCAGAGCTTCCTCAAAGACGACTCAATTGACAATAAGGTGCTGACTCGCTCAGACAAGAACCGGGGAAAGTCAGATAACGTGCCCTCAGAGGAAGTCGTGAAAAAGATGAAGAACTATTGGCGCCAGCTTCTGAACGCAAAGCTGATCACTCAGCGGAAGTTCGACAATCTCACTAAGGCTGAGAGGGGCGGACTGAGCGAACTGGACAAAGCAGGATTCATTAAACGGCAACTTGTGGAGACTCGGCAGATTACTAAACATGTCGCCCAAATCCTTGACTCACGCATGAATACCAAGTACGACGAAAACGACAAACTTATCCGCGAGGTGAAGGTGATTACCCTGAAGTCCAAGCTGGTCAGCGATTTCAGAAAGGACTTTCAATTCTACAAAGTGCGGGAGATCAATAACTATCATCATGCTCATGACGCATATCTGAATGCCGTGGTGGGAACCGCCCTGATCAAGAAGTACCCAAAGCTGGAAAGCGAGTTCGTGTACGGAGACTACAAGGTCTACGACGTGCGCAAGATGATTGCCAAATCTGAGCAGGAGATCGGAAAGGCCACCGCAAAGTACTTCTTCTACAGCAACATCATGAATTTCTTCAAGACCGAAATCACCCTTGCAAACGGTGAGATCCGGAAGAGGCCGCTCATCGAGACTAATGGGGAGACTGGCGAAATCGTGTGGGACAAGGGCAGAGATTTCGCTACCGTGCGCAAAGTGCTTTCTATGCCTCAAGTGAACATCGTGAAGAAAACCGAGGTGCAAACCGGAGGCTTTTCTAAGGAATCAATCCTCCCCAAGCGCAACTCCGACAAGCTCATTGCAAGGAAGAAGGATTGGGACCCTAAGAAGTACGGCGGATTCGATTCACCAACTGTGGCTTATTCTGTCCTGGTCGTGGCTAAGGTGGAAAAAGGAAAGTCTAAGAAGCTCAAGAGCGTGAAGGAACTGCTGGGTATCACCATTATGGAGCGCAGCTCCTTCGAGAAGAACCCAATTGACTTTCTCGAAGCCAAAGGTTACAAGGAAGTCAAGAAGGACCTTATCATCAAGCTCCCAAAGTATAGCCTGTTCGAACTGGAGAATGGGCGGAAGCGGATGCTCGCCTCCGCTGGCGAACTTCAGAAGGGTAATGAGCTGGCTCTCCCCTCCAAGTACGTGAATTTCCTCTACCTTGCAAGCCATTACGAGAAGCTGAAGGGGAGCCCCGAGGACAACGAGCAAAAGCAACTGTTTGTGGAGCAGCATAAGCATTATCTGGACGAGATCATTGAGCAGATTTCCGAGTTTTCTAAACGCGTCATTCTCGCTGATGCCAACCTCGATAAAGTCCTTAGCGCATACAATAAGCACAGAGACAAACCAATTCGGGAGCAGGCTGAGAATATCATCCACCTGTTCACCCTCACCAATCTTGGTGCCCCTGCCGCATTCAAGTACTTCGACACCACCATCGACCGGAAACGCTATACCTCCACCAAAGAAGTGCTGGACGCCACCCTCATCCACCAGAGCATCACCGGACTTTACGAAACTCGGATTGACCTCTCACAGCTCGGAGGGGATGAGGGAGCTGATCCAAAAAAGAAGAGAAAGGTAGATCCAAAAAAGAAGAGAAAGGTAGATCCAAAAAAGAAGAGAAAGGTATAG

- 1. Amino acid sequence

MDYKDHDGDYKDHDIDYKDDDDKMAPKKKRKVGIHRGVPGGSMGSQLVKSELEEKKSELRHKLKYVPHEYIELIEIARNSTQDRILEMKVMEFFMKVYGYRGKHLGGSRKPDGAIYTVGSPIDYGVIVDTKAYSGGYNLPIGQADEMQRYVEENQTRNKHINPNEWWKVYPSSVTEFKFLFVSGHFKGNYKAQLTRLNHITNCNGAVLSVEELLIGGEMIKAGTLTLEEVRRKFNNGEINFSGSETPGTSESATPETMDKKYSIGLAIGTNSVGWAVITDEYKVPSKKFKVLGNTDRHSIKKNLIGALLFDSGETAEATRLKRTARRRYTRRKNRICYLQEIFSNEMAKVDDSFFHRLEESFLVEEDKKHERHPIFGNIVDEVAYHEKYPTIYHLRKKLVDSTDKADLRLIYLALAHMIKFRGHFLIEGDLNPDNSDVDKLFIQLVQTYNQLFEENPINASGVDAKAILSARLSKSRRLENLIAQLPGEKKNGLFGNLIALSLGLTPNFKSNFDLAEDAKLQLSKDTYDDDLDNLLAQIGDQYADLFLAAKNLSDAILLSDILRVNTEITKAPLSASMIKRYDEHHQDLTLLKALVRQQLPEKYKEIFFDQSKNGYAGYIDGGASQEEFYKFIKPILEKMDGTEELLVKLNREDLLRKQRTFDNGSIPHQIHLGELHAILRRQEDFYPFLKDNREKIEKILTFRIPYYVGPLARGNSRFAWMTRKSEETITPWNFEEVVDKGASAQSFIERMTNFDKNLPNEKVLPKHSLLYEYFTVYNELTKVKYVTEGMRKPAFLSGEQKKAIVDLLFKTNRKVTVKQLKEDYFKKIECFDSVEISGVEDRFNASLGTYHDLLKIIKDKDFLDNEENEDILEDIVLTLTLFEDREMIEERLKTYAHLFDDKVMKQLKRRRYTGWGRLSRKLINGIRDKQSGKTILDFLKSDGFANRNFMQLIHDDSLTFKEDIQKAQVSGQGDSLHEHIANLAGSPAIKKGILQTVKVVDELVKVMGRHKPENIVIEMARENQTTQKGQKNSRERMKRIEEGIKELGSQILKEHPVENTQLQNEKLYLYYLQNGRDMYVDQELDINRLSDYDVDAIVPQSFLKDDSIDNKVLTRSDKNRGKSDNVPSEEVVKKMKNYWRQLLNAKLITQRKFDNLTKAERGGLSELDKAGFIKRQLVETRQITKHVAQILDSRMNTKYDENDKLIREVKVITLKSKLVSDFRKDFQFYKVREINNYHHAHDAYLNAVVGTALIKKYPKLESEFVYGDYKVYDVRKMIAKSEQEIGKATAKYFFYSNIMNFFKTEITLANGEIRKRPLIETNGETGEIVWDKGRDFATVRKVLSMPQVNIVKKTEVQTGGFSKESILPKRNSDKLIARKKDWDPKKYGGFDSPTVAYSVLVVAKVEKGKSKKLKSVKELLGITIMERSSFEKNPIDFLEAKGYKEVKKDLIIKLPKYSLFELENGRKRMLASAGELQKGNELALPSKYVNFLYLASHYEKLKGSPEDNEQKQLFVEQHKHYLDEIIEQISEFSKRVILADANLDKVLSAYNKHRDKPIREQAENIIHLFTLTNLGAPAAFKYFDTTIDRKRYTSTKEVLDATLIHQSITGLYETRIDLSQLGGDEGADPKKKRKVDPKKKRKVDPKKKRKV*

1. Sharky.fdCas9
   1. DNA sequence

ATGGACTACAAAGACCATGACGGTGATTATAAAGATCATGACATCGATTACAAGGATGACGATGACAAGATGGCCCCCAAGAAGAAGAGGAAGGTGGGCATTCACCGCGGGGTACCTGGAGGTTCTATGGGATCCCAACTAGTCAAAAGTGAACTGGAGGAGAAGAAATCTGAACTTCGTCATAAATTGAAATATGTGCCTCATGAATATATTGAATTAATTGAAATTGCCAGAAATCCCACTCAGGATAGAATTCTTGAAATGAAGGTAATGGAATTTTTTATGAAAGTTTATGGATATAGAGGTGAACATTTGGGTGGATCAAGGAAACCGGACGGAGCAATTTATACTGTCGGATCTCCTATTGATTACGGTGTGATCGTGGATACTAAAGCTTATAGCGGAGGTTATAATCTGCCAATTGGCCAAGCAGATGAAATGCAACGATATGTCGAAGAAAATCAAACACGAAACAAACATATCAACCCTAATGAATGGTGGAAAGTCTATCCATCTTCTGTAACGGAATTTAAGTTTTTATTTGTGAGTGGTCACTTTAAAGGAAACTACAAAGCTCAGCTTACACGATTAAATCATATCACTAATTGTAATGGAGCTGTTCTTAGTGTAGAAGAGCTTTTAATTGGTGGAGAAATGATTAAAGCCGGCACATTAACCTTAGAGGAAGTGAGACGGAAATTTAATAACGGCGAGATAAACTTTAGCGGCAGCGAGACTCCCGGGACCTCAGAGTCCGCCACACCCGAAACCATGGACAAGAAGTATTCTATCGGACTGGCCATCGGGACTAATAGCGTCGGGTGGGCCGTGATCACTGACGAGTACAAGGTGCCCTCTAAGAAGTTCAAGGTGCTCGGGAACACCGACCGGCATTCCATCAAGAAAAATCTGATCGGAGCTCTCCTCTTTGATTCAGGGGAGACCGCTGAAGCAACCCGCCTCAAGCGGACTGCTAGACGGCGGTACACCAGGAGGAAGAACCGGATTTGTTACCTTCAAGAGATATTCTCCAACGAAATGGCAAAGGTCGACGACAGCTTCTTCCATAGGCTGGAAGAATCATTCCTCGTGGAAGAGGATAAGAAGCATGAACGGCATCCCATCTTCGGTAATATCGTCGACGAGGTGGCCTATCACGAGAAATACCCAACCATCTACCATCTTCGCAAAAAGCTGGTGGACTCAACCGACAAGGCAGACCTCCGGCTTATCTACCTGGCCCTGGCCCACATGATCAAGTTCAGAGGCCACTTCCTGATCGAGGGCGACCTCAATCCTGACAATAGCGATGTGGATAAACTGTTCATCCAGCTGGTGCAGACTTACAACCAGCTCTTTGAAGAGAACCCCATCAATGCAAGCGGAGTCGATGCCAAGGCCATTCTGTCAGCCCGGCTGTCAAAGAGCCGCAGACTTGAGAATCTTATCGCTCAGCTGCCGGGTGAAAAGAAAAATGGACTGTTCGGGAACCTGATTGCTCTTTCACTTGGGCTGACTCCCAATTTCAAGTCTAATTTCGACCTGGCAGAGGATGCCAAGCTGCAACTGTCCAAGGACACCTATGATGACGATCTCGACAACCTCCTGGCCCAGATCGGTGACCAATACGCCGACCTTTTCCTTGCTGCTAAGAATCTTTCTGACGCCATCCTGCTGTCTGACATTCTCCGCGTGAACACTGAAATCACCAAGGCCCCTCTTTCAGCTTCAATGATTAAGCGGTATGATGAGCACCACCAGGACCTGACCCTGCTTAAGGCACTCGTCCGGCAGCAGCTTCCGGAGAAGTACAAGGAAATCTTCTTTGACCAGTCAAAGAATGGATACGCCGGCTACATCGACGGAGGTGCCTCCCAAGAGGAATTTTATAAGTTTATCAAACCTATCCTTGAGAAGATGGACGGCACCGAAGAGCTCCTCGTGAAACTGAATCGGGAGGATCTGCTGCGGAAGCAGCGCACTTTCGACAATGGGAGCATTCCCCACCAGATCCATCTTGGGGAGCTTCACGCCATCCTTCGGCGCCAAGAGGACTTCTACCCCTTTCTTAAGGACAACAGGGAGAAGATTGAGAAAATTCTCACTTTCCGCATCCCCTACTACGTGGGACCCCTCGCCAGAGGAAATAGCCGGTTTGCTTGGATGACCAGAAAGTCAGAAGAAACTATCACTCCCTGGAACTTCGAAGAGGTGGTGGACAAGGGAGCCAGCGCTCAGTCATTCATCGAACGGATGACTAACTTCGATAAGAACCTCCCCAATGAGAAGGTCCTGCCGAAACATTCCCTGCTCTACGAGTACTTTACCGTGTACAACGAGCTGACCAAGGTGAAATATGTCACCGAAGGGATGAGGAAGCCCGCATTCCTGTCAGGCGAACAAAAGAAGGCAATTGTGGACCTTCTGTTCAAGACCAATAGAAAGGTGACCGTGAAGCAGCTGAAGGAGGACTATTTCAAGAAAATTGAATGCTTCGACTCTGTGGAGATTAGCGGGGTCGAAGATCGGTTCAACGCAAGCCTGGGTACCTACCATGATCTGCTTAAGATCATCAAGGACAAGGATTTTCTGGACAATGAGGAGAACGAGGACATCCTTGAGGACATTGTCCTGACTCTCACTCTGTTCGAGGACCGGGAAATGATCGAGGAGAGGCTTAAGACCTACGCCCATCTGTTCGACGATAAAGTGATGAAGCAACTTAAACGGAGAAGATATACCGGATGGGGACGCCTTAGCCGCAAACTCATCAACGGAATCCGGGACAAACAGAGCGGAAAGACCATTCTTGATTTCCTTAAGAGCGACGGATTCGCTAATCGCAACTTCATGCAACTTATCCATGATGATTCCCTGACCTTTAAGGAGGACATCCAGAAGGCCCAAGTGTCTGGACAAGGTGACTCACTGCACGAGCATATCGCAAATCTGGCTGGTTCACCCGCTATTAAGAAGGGTATTCTCCAGACCGTGAAAGTCGTGGACGAGCTGGTCAAGGTGATGGGTCGCCATAAACCAGAGAACATTGTCATCGAGATGGCCAGGGAAAACCAGACTACCCAGAAGGGACAGAAGAACAGCAGGGAGCGGATGAAAAGAATTGAGGAAGGGATTAAGGAGCTCGGGTCACAGATCCTTAAAGAGCACCCGGTGGAAAACACCCAGCTTCAGAATGAGAAGCTCTATCTGTACTACCTTCAAAATGGACGCGATATGTATGTGGACCAAGAGCTTGATATCAACAGGCTCTCAGACTACGACGTGGACGCCATCGTCCCTCAGAGCTTCCTCAAAGACGACTCAATTGACAATAAGGTGCTGACTCGCTCAGACAAGAACCGGGGAAAGTCAGATAACGTGCCCTCAGAGGAAGTCGTGAAAAAGATGAAGAACTATTGGCGCCAGCTTCTGAACGCAAAGCTGATCACTCAGCGGAAGTTCGACAATCTCACTAAGGCTGAGAGGGGCGGACTGAGCGAACTGGACAAAGCAGGATTCATTAAACGGCAACTTGTGGAGACTCGGCAGATTACTAAACATGTCGCCCAAATCCTTGACTCACGCATGAATACCAAGTACGACGAAAACGACAAACTTATCCGCGAGGTGAAGGTGATTACCCTGAAGTCCAAGCTGGTCAGCGATTTCAGAAAGGACTTTCAATTCTACAAAGTGCGGGAGATCAATAACTATCATCATGCTCATGACGCATATCTGAATGCCGTGGTGGGAACCGCCCTGATCAAGAAGTACCCAAAGCTGGAAAGCGAGTTCGTGTACGGAGACTACAAGGTCTACGACGTGCGCAAGATGATTGCCAAATCTGAGCAGGAGATCGGAAAGGCCACCGCAAAGTACTTCTTCTACAGCAACATCATGAATTTCTTCAAGACCGAAATCACCCTTGCAAACGGTGAGATCCGGAAGAGGCCGCTCATCGAGACTAATGGGGAGACTGGCGAAATCGTGTGGGACAAGGGCAGAGATTTCGCTACCGTGCGCAAAGTGCTTTCTATGCCTCAAGTGAACATCGTGAAGAAAACCGAGGTGCAAACCGGAGGCTTTTCTAAGGAATCAATCCTCCCCAAGCGCAACTCCGACAAGCTCATTGCAAGGAAGAAGGATTGGGACCCTAAGAAGTACGGCGGATTCGATTCACCAACTGTGGCTTATTCTGTCCTGGTCGTGGCTAAGGTGGAAAAAGGAAAGTCTAAGAAGCTCAAGAGCGTGAAGGAACTGCTGGGTATCACCATTATGGAGCGCAGCTCCTTCGAGAAGAACCCAATTGACTTTCTCGAAGCCAAAGGTTACAAGGAAGTCAAGAAGGACCTTATCATCAAGCTCCCAAAGTATAGCCTGTTCGAACTGGAGAATGGGCGGAAGCGGATGCTCGCCTCCGCTGGCGAACTTCAGAAGGGTAATGAGCTGGCTCTCCCCTCCAAGTACGTGAATTTCCTCTACCTTGCAAGCCATTACGAGAAGCTGAAGGGGAGCCCCGAGGACAACGAGCAAAAGCAACTGTTTGTGGAGCAGCATAAGCATTATCTGGACGAGATCATTGAGCAGATTTCCGAGTTTTCTAAACGCGTCATTCTCGCTGATGCCAACCTCGATAAAGTCCTTAGCGCATACAATAAGCACAGAGACAAACCAATTCGGGAGCAGGCTGAGAATATCATCCACCTGTTCACCCTCACCAATCTTGGTGCCCCTGCCGCATTCAAGTACTTCGACACCACCATCGACCGGAAACGCTATACCTCCACCAAAGAAGTGCTGGACGCCACCCTCATCCACCAGAGCATCACCGGACTTTACGAAACTCGGATTGACCTCTCACAGCTCGGAGGGGATGAGGGAGCTGATCCAAAAAAGAAGAGAAAGGTAGATCCAAAAAAGAAGAGAAAGGTAGATCCAAAAAAGAAGAGAAAGGTATAG

- 1. Amino acid sequence

MDYKDHDGDYKDHDIDYKDDDDKMAPKKKRKVGIHRGVPGGSMGSQLVKSELEEKKSELRHKLKYVPHEYIELIEIARNPTQDRILEMKVMEFFMKVYGYRGEHLGGSRKPDGAIYTVGSPIDYGVIVDTKAYSGGYNLPIGQADEMQRYVEENQTRNKHINPNEWWKVYPSSVTEFKFLFVSGHFKGNYKAQLTRLNHITNCNGAVLSVEELLIGGEMIKAGTLTLEEVRRKFNNGEINFSGSETPGTSESATPETMDKKYSIGLAIGTNSVGWAVITDEYKVPSKKFKVLGNTDRHSIKKNLIGALLFDSGETAEATRLKRTARRRYTRRKNRICYLQEIFSNEMAKVDDSFFHRLEESFLVEEDKKHERHPIFGNIVDEVAYHEKYPTIYHLRKKLVDSTDKADLRLIYLALAHMIKFRGHFLIEGDLNPDNSDVDKLFIQLVQTYNQLFEENPINASGVDAKAILSARLSKSRRLENLIAQLPGEKKNGLFGNLIALSLGLTPNFKSNFDLAEDAKLQLSKDTYDDDLDNLLAQIGDQYADLFLAAKNLSDAILLSDILRVNTEITKAPLSASMIKRYDEHHQDLTLLKALVRQQLPEKYKEIFFDQSKNGYAGYIDGGASQEEFYKFIKPILEKMDGTEELLVKLNREDLLRKQRTFDNGSIPHQIHLGELHAILRRQEDFYPFLKDNREKIEKILTFRIPYYVGPLARGNSRFAWMTRKSEETITPWNFEEVVDKGASAQSFIERMTNFDKNLPNEKVLPKHSLLYEYFTVYNELTKVKYVTEGMRKPAFLSGEQKKAIVDLLFKTNRKVTVKQLKEDYFKKIECFDSVEISGVEDRFNASLGTYHDLLKIIKDKDFLDNEENEDILEDIVLTLTLFEDREMIEERLKTYAHLFDDKVMKQLKRRRYTGWGRLSRKLINGIRDKQSGKTILDFLKSDGFANRNFMQLIHDDSLTFKEDIQKAQVSGQGDSLHEHIANLAGSPAIKKGILQTVKVVDELVKVMGRHKPENIVIEMARENQTTQKGQKNSRERMKRIEEGIKELGSQILKEHPVENTQLQNEKLYLYYLQNGRDMYVDQELDINRLSDYDVDAIVPQSFLKDDSIDNKVLTRSDKNRGKSDNVPSEEVVKKMKNYWRQLLNAKLITQRKFDNLTKAERGGLSELDKAGFIKRQLVETRQITKHVAQILDSRMNTKYDENDKLIREVKVITLKSKLVSDFRKDFQFYKVREINNYHHAHDAYLNAVVGTALIKKYPKLESEFVYGDYKVYDVRKMIAKSEQEIGKATAKYFFYSNIMNFFKTEITLANGEIRKRPLIETNGETGEIVWDKGRDFATVRKVLSMPQVNIVKKTEVQTGGFSKESILPKRNSDKLIARKKDWDPKKYGGFDSPTVAYSVLVVAKVEKGKSKKLKSVKELLGITIMERSSFEKNPIDFLEAKGYKEVKKDLIIKLPKYSLFELENGRKRMLASAGELQKGNELALPSKYVNFLYLASHYEKLKGSPEDNEQKQLFVEQHKHYLDEIIEQISEFSKRVILADANLDKVLSAYNKHRDKPIREQAENIIHLFTLTNLGAPAAFKYFDTTIDRKRYTSTKEVLDATLIHQSITGLYETRIDLSQLGGDEGADPKKKRKVDPKKKRKVDPKKKRKV*

1. dCas9f (3GS-3NLS)
   1. DNA sequence

ATGGACAAGAAGTATTCTATCGGACTGGCCATCGGGACTAATAGCGTCGGGTGGGCCGTGATCACTGACGAGTACAAGGTGCCCTCTAAGAAGTTCAAGGTGCTCGGGAACACCGACCGGCATTCCATCAAGAAAAATCTGATCGGAGCTCTCCTCTTTGATTCAGGGGAGACCGCTGAAGCAACCCGCCTCAAGCGGACTGCTAGACGGCGGTACACCAGGAGGAAGAACCGGATTTGTTACCTTCAAGAGATATTCTCCAACGAAATGGCAAAGGTCGACGACAGCTTCTTCCATAGGCTGGAAGAATCATTCCTCGTGGAAGAGGATAAGAAGCATGAACGGCATCCCATCTTCGGTAATATCGTCGACGAGGTGGCCTATCACGAGAAATACCCAACCATCTACCATCTTCGCAAAAAGCTGGTGGACTCAACCGACAAGGCAGACCTCCGGCTTATCTACCTGGCCCTGGCCCACATGATCAAGTTCAGAGGCCACTTCCTGATCGAGGGCGACCTCAATCCTGACAATAGCGATGTGGATAAACTGTTCATCCAGCTGGTGCAGACTTACAACCAGCTCTTTGAAGAGAACCCCATCAATGCAAGCGGAGTCGATGCCAAGGCCATTCTGTCAGCCCGGCTGTCAAAGAGCCGCAGACTTGAGAATCTTATCGCTCAGCTGCCGGGTGAAAAGAAAAATGGACTGTTCGGGAACCTGATTGCTCTTTCACTTGGGCTGACTCCCAATTTCAAGTCTAATTTCGACCTGGCAGAGGATGCCAAGCTGCAACTGTCCAAGGACACCTATGATGACGATCTCGACAACCTCCTGGCCCAGATCGGTGACCAATACGCCGACCTTTTCCTTGCTGCTAAGAATCTTTCTGACGCCATCCTGCTGTCTGACATTCTCCGCGTGAACACTGAAATCACCAAGGCCCCTCTTTCAGCTTCAATGATTAAGCGGTATGATGAGCACCACCAGGACCTGACCCTGCTTAAGGCACTCGTCCGGCAGCAGCTTCCGGAGAAGTACAAGGAAATCTTCTTTGACCAGTCAAAGAATGGATACGCCGGCTACATCGACGGAGGTGCCTCCCAAGAGGAATTTTATAAGTTTATCAAACCTATCCTTGAGAAGATGGACGGCACCGAAGAGCTCCTCGTGAAACTGAATCGGGAGGATCTGCTGCGGAAGCAGCGCACTTTCGACAATGGGAGCATTCCCCACCAGATCCATCTTGGGGAGCTTCACGCCATCCTTCGGCGCCAAGAGGACTTCTACCCCTTTCTTAAGGACAACAGGGAGAAGATTGAGAAAATTCTCACTTTCCGCATCCCCTACTACGTGGGACCCCTCGCCAGAGGAAATAGCCGGTTTGCTTGGATGACCAGAAAGTCAGAAGAAACTATCACTCCCTGGAACTTCGAAGAGGTGGTGGACAAGGGAGCCAGCGCTCAGTCATTCATCGAACGGATGACTAACTTCGATAAGAACCTCCCCAATGAGAAGGTCCTGCCGAAACATTCCCTGCTCTACGAGTACTTTACCGTGTACAACGAGCTGACCAAGGTGAAATATGTCACCGAAGGGATGAGGAAGCCCGCATTCCTGTCAGGCGAACAAAAGAAGGCAATTGTGGACCTTCTGTTCAAGACCAATAGAAAGGTGACCGTGAAGCAGCTGAAGGAGGACTATTTCAAGAAAATTGAATGCTTCGACTCTGTGGAGATTAGCGGGGTCGAAGATCGGTTCAACGCAAGCCTGGGTACCTACCATGATCTGCTTAAGATCATCAAGGACAAGGATTTTCTGGACAATGAGGAGAACGAGGACATCCTTGAGGACATTGTCCTGACTCTCACTCTGTTCGAGGACCGGGAAATGATCGAGGAGAGGCTTAAGACCTACGCCCATCTGTTCGACGATAAAGTGATGAAGCAACTTAAACGGAGAAGATATACCGGATGGGGACGCCTTAGCCGCAAACTCATCAACGGAATCCGGGACAAACAGAGCGGAAAGACCATTCTTGATTTCCTTAAGAGCGACGGATTCGCTAATCGCAACTTCATGCAACTTATCCATGATGATTCCCTGACCTTTAAGGAGGACATCCAGAAGGCCCAAGTGTCTGGACAAGGTGACTCACTGCACGAGCATATCGCAAATCTGGCTGGTTCACCCGCTATTAAGAAGGGTATTCTCCAGACCGTGAAAGTCGTGGACGAGCTGGTCAAGGTGATGGGTCGCCATAAACCAGAGAACATTGTCATCGAGATGGCCAGGGAAAACCAGACTACCCAGAAGGGACAGAAGAACAGCAGGGAGCGGATGAAAAGAATTGAGGAAGGGATTAAGGAGCTCGGGTCACAGATCCTTAAAGAGCACCCGGTGGAAAACACCCAGCTTCAGAATGAGAAGCTCTATCTGTACTACCTTCAAAATGGACGCGATATGTATGTGGACCAAGAGCTTGATATCAACAGGCTCTCAGACTACGACGTGGACGCCATCGTCCCTCAGAGCTTCCTCAAAGACGACTCAATTGACAATAAGGTGCTGACTCGCTCAGACAAGAACCGGGGAAAGTCAGATAACGTGCCCTCAGAGGAAGTCGTGAAAAAGATGAAGAACTATTGGCGCCAGCTTCTGAACGCAAAGCTGATCACTCAGCGGAAGTTCGACAATCTCACTAAGGCTGAGAGGGGCGGACTGAGCGAACTGGACAAAGCAGGATTCATTAAACGGCAACTTGTGGAGACTCGGCAGATTACTAAACATGTCGCCCAAATCCTTGACTCACGCATGAATACCAAGTACGACGAAAACGACAAACTTATCCGCGAGGTGAAGGTGATTACCCTGAAGTCCAAGCTGGTCAGCGATTTCAGAAAGGACTTTCAATTCTACAAAGTGCGGGAGATCAATAACTATCATCATGCTCATGACGCATATCTGAATGCCGTGGTGGGAACCGCCCTGATCAAGAAGTACCCAAAGCTGGAAAGCGAGTTCGTGTACGGAGACTACAAGGTCTACGACGTGCGCAAGATGATTGCCAAATCTGAGCAGGAGATCGGAAAGGCCACCGCAAAGTACTTCTTCTACAGCAACATCATGAATTTCTTCAAGACCGAAATCACCCTTGCAAACGGTGAGATCCGGAAGAGGCCGCTCATCGAGACTAATGGGGAGACTGGCGAAATCGTGTGGGACAAGGGCAGAGATTTCGCTACCGTGCGCAAAGTGCTTTCTATGCCTCAAGTGAACATCGTGAAGAAAACCGAGGTGCAAACCGGAGGCTTTTCTAAGGAATCAATCCTCCCCAAGCGCAACTCCGACAAGCTCATTGCAAGGAAGAAGGATTGGGACCCTAAGAAGTACGGCGGATTCGATTCACCAACTGTGGCTTATTCTGTCCTGGTCGTGGCTAAGGTGGAAAAAGGAAAGTCTAAGAAGCTCAAGAGCGTGAAGGAACTGCTGGGTATCACCATTATGGAGCGCAGCTCCTTCGAGAAGAACCCAATTGACTTTCTCGAAGCCAAAGGTTACAAGGAAGTCAAGAAGGACCTTATCATCAAGCTCCCAAAGTATAGCCTGTTCGAACTGGAGAATGGGCGGAAGCGGATGCTCGCCTCCGCTGGCGAACTTCAGAAGGGTAATGAGCTGGCTCTCCCCTCCAAGTACGTGAATTTCCTCTACCTTGCAAGCCATTACGAGAAGCTGAAGGGGAGCCCCGAGGACAACGAGCAAAAGCAACTGTTTGTGGAGCAGCATAAGCATTATCTGGACGAGATCATTGAGCAGATTTCCGAGTTTTCTAAACGCGTCATTCTCGCTGATGCCAACCTCGATAAAGTCCTTAGCGCATACAATAAGCACAGAGACAAACCAATTCGGGAGCAGGCTGAGAATATCATCCACCTGTTCACCCTCACCAATCTTGGTGCCCCTGCCGCATTCAAGTACTTCGACACCACCATCGACCGGAAACGCTATACCTCCACCAAAGAAGTGCTGGACGCCACCCTCATCCACCAGAGCATCACCGGACTTTACGAAACTCGGATTGACCTCTCACAGCTCGGAGGGGATGAGGGAGCTGATGATCCAAAAAAGAAGAGAAAGGTAGATCCAAAAAAGAAGAGAAAGGTAGATCCAAAAAAGAAGAGAAAGGTAGGGCCcGGTTCCGGTTCCGGTTCCCAACTCGTGAAGAGTGAACTTGAGGAGAAAAAGTCGGAGCTGCGGCACAAATTGAAATACGTACCGCATGAATACATCGAACTTATCGAAATTGCTAGGAACTCGACTCAAGACAGAATCCTTGAGATGAAGGTAATGGAGTTCTTTATGAAGGTTTATGGATACCGAGGGAAGCATCTCGGTGGATCACGAAAACCCGACGGAGCAATCTATACGGTGGGGAGCCCGATTGATTACGGAGTGATCGTCGACACGAAAGCCTACAGCGGTGGGTACAATCTTCCCATCGGGCAGGCAGATGAGATGCAACGTTATGTCGAAGAAAATCAGACCAGGAACAAACACATCAATCCAAATGAGTGGTGGAAAGTGTATCCTTCATCAGTGACCGAGTTTAAGTTTTTGTTTGTCTCTGGGCATTTCAAAGGCAACTATAAGGCCCAGCTCACACGGTTGAATCACATTACGAACTGCAATGGTGCGGTTTTGTCCGTAGAGGAACTGCTCATTGGTGGAGAAATGATCAAAGCGGGAACTCTGACACTGGAAGAAGTCAGACGCAAGTTTAACAATGGCGAGATCAATTTCCGCTCATAA

- 1. Amino acid sequence

MDKKYSIGLAIGTNSVGWAVITDEYKVPSKKFKVLGNTDRHSIKKNLIGALLFDSGETAEATRLKRTARRRYTRRKNRICYLQEIFSNEMAKVDDSFFHRLEESFLVEEDKKHERHPIFGNIVDEVAYHEKYPTIYHLRKKLVDSTDKADLRLIYLALAHMIKFRGHFLIEGDLNPDNSDVDKLFIQLVQTYNQLFEENPINASGVDAKAILSARLSKSRRLENLIAQLPGEKKNGLFGNLIALSLGLTPNFKSNFDLAEDAKLQLSKDTYDDDLDNLLAQIGDQYADLFLAAKNLSDAILLSDILRVNTEITKAPLSASMIKRYDEHHQDLTLLKALVRQQLPEKYKEIFFDQSKNGYAGYIDGGASQEEFYKFIKPILEKMDGTEELLVKLNREDLLRKQRTFDNGSIPHQIHLGELHAILRRQEDFYPFLKDNREKIEKILTFRIPYYVGPLARGNSRFAWMTRKSEETITPWNFEEVVDKGASAQSFIERMTNFDKNLPNEKVLPKHSLLYEYFTVYNELTKVKYVTEGMRKPAFLSGEQKKAIVDLLFKTNRKVTVKQLKEDYFKKIECFDSVEISGVEDRFNASLGTYHDLLKIIKDKDFLDNEENEDILEDIVLTLTLFEDREMIEERLKTYAHLFDDKVMKQLKRRRYTGWGRLSRKLINGIRDKQSGKTILDFLKSDGFANRNFMQLIHDDSLTFKEDIQKAQVSGQGDSLHEHIANLAGSPAIKKGILQTVKVVDELVKVMGRHKPENIVIEMARENQTTQKGQKNSRERMKRIEEGIKELGSQILKEHPVENTQLQNEKLYLYYLQNGRDMYVDQELDINRLSDYDVDAIVPQSFLKDDSIDNKVLTRSDKNRGKSDNVPSEEVVKKMKNYWRQLLNAKLITQRKFDNLTKAERGGLSELDKAGFIKRQLVETRQITKHVAQILDSRMNTKYDENDKLIREVKVITLKSKLVSDFRKDFQFYKVREINNYHHAHDAYLNAVVGTALIKKYPKLESEFVYGDYKVYDVRKMIAKSEQEIGKATAKYFFYSNIMNFFKTEITLANGEIRKRPLIETNGETGEIVWDKGRDFATVRKVLSMPQVNIVKKTEVQTGGFSKESILPKRNSDKLIARKKDWDPKKYGGFDSPTVAYSVLVVAKVEKGKSKKLKSVKELLGITIMERSSFEKNPIDFLEAKGYKEVKKDLIIKLPKYSLFELENGRKRMLASAGELQKGNELALPSKYVNFLYLASHYEKLKGSPEDNEQKQLFVEQHKHYLDEIIEQISEFSKRVILADANLDKVLSAYNKHRDKPIREQAENIIHLFTLTNLGAPAAFKYFDTTIDRKRYTSTKEVLDATLIHQSITGLYETRIDLSQLGGDEGADDPKKKRKVDPKKKRKVDPKKKRKVGPGSGSGSQLVKSELEEKKSELRHKLKYVPHEYIELIEIARNSTQDRILEMKVMEFFMKVYGYRGKHLGGSRKPDGAIYTVGSPIDYGVIVDTKAYSGGYNLPIGQADEMQRYVEENQTRNKHINPNEWWKVYPSSVTEFKFLFVSGHFKGNYKAQLTRLNHITNCNGAVLSVEELLIGGEMIKAGTLTLEEVRRKFNNGEINFRS*

1. dCas9f (3GS-3NLS)
   1. DNA sequence

ATGGACAAGAAGTATTCTATCGGACTGGCCATCGGGACTAATAGCGTCGGGTGGGCCGTGATCACTGACGAGTACAAGGTGCCCTCTAAGAAGTTCAAGGTGCTCGGGAACACCGACCGGCATTCCATCAAGAAAAATCTGATCGGAGCTCTCCTCTTTGATTCAGGGGAGACCGCTGAAGCAACCCGCCTCAAGCGGACTGCTAGACGGCGGTACACCAGGAGGAAGAACCGGATTTGTTACCTTCAAGAGATATTCTCCAACGAAATGGCAAAGGTCGACGACAGCTTCTTCCATAGGCTGGAAGAATCATTCCTCGTGGAAGAGGATAAGAAGCATGAACGGCATCCCATCTTCGGTAATATCGTCGACGAGGTGGCCTATCACGAGAAATACCCAACCATCTACCATCTTCGCAAAAAGCTGGTGGACTCAACCGACAAGGCAGACCTCCGGCTTATCTACCTGGCCCTGGCCCACATGATCAAGTTCAGAGGCCACTTCCTGATCGAGGGCGACCTCAATCCTGACAATAGCGATGTGGATAAACTGTTCATCCAGCTGGTGCAGACTTACAACCAGCTCTTTGAAGAGAACCCCATCAATGCAAGCGGAGTCGATGCCAAGGCCATTCTGTCAGCCCGGCTGTCAAAGAGCCGCAGACTTGAGAATCTTATCGCTCAGCTGCCGGGTGAAAAGAAAAATGGACTGTTCGGGAACCTGATTGCTCTTTCACTTGGGCTGACTCCCAATTTCAAGTCTAATTTCGACCTGGCAGAGGATGCCAAGCTGCAACTGTCCAAGGACACCTATGATGACGATCTCGACAACCTCCTGGCCCAGATCGGTGACCAATACGCCGACCTTTTCCTTGCTGCTAAGAATCTTTCTGACGCCATCCTGCTGTCTGACATTCTCCGCGTGAACACTGAAATCACCAAGGCCCCTCTTTCAGCTTCAATGATTAAGCGGTATGATGAGCACCACCAGGACCTGACCCTGCTTAAGGCACTCGTCCGGCAGCAGCTTCCGGAGAAGTACAAGGAAATCTTCTTTGACCAGTCAAAGAATGGATACGCCGGCTACATCGACGGAGGTGCCTCCCAAGAGGAATTTTATAAGTTTATCAAACCTATCCTTGAGAAGATGGACGGCACCGAAGAGCTCCTCGTGAAACTGAATCGGGAGGATCTGCTGCGGAAGCAGCGCACTTTCGACAATGGGAGCATTCCCCACCAGATCCATCTTGGGGAGCTTCACGCCATCCTTCGGCGCCAAGAGGACTTCTACCCCTTTCTTAAGGACAACAGGGAGAAGATTGAGAAAATTCTCACTTTCCGCATCCCCTACTACGTGGGACCCCTCGCCAGAGGAAATAGCCGGTTTGCTTGGATGACCAGAAAGTCAGAAGAAACTATCACTCCCTGGAACTTCGAAGAGGTGGTGGACAAGGGAGCCAGCGCTCAGTCATTCATCGAACGGATGACTAACTTCGATAAGAACCTCCCCAATGAGAAGGTCCTGCCGAAACATTCCCTGCTCTACGAGTACTTTACCGTGTACAACGAGCTGACCAAGGTGAAATATGTCACCGAAGGGATGAGGAAGCCCGCATTCCTGTCAGGCGAACAAAAGAAGGCAATTGTGGACCTTCTGTTCAAGACCAATAGAAAGGTGACCGTGAAGCAGCTGAAGGAGGACTATTTCAAGAAAATTGAATGCTTCGACTCTGTGGAGATTAGCGGGGTCGAAGATCGGTTCAACGCAAGCCTGGGTACCTACCATGATCTGCTTAAGATCATCAAGGACAAGGATTTTCTGGACAATGAGGAGAACGAGGACATCCTTGAGGACATTGTCCTGACTCTCACTCTGTTCGAGGACCGGGAAATGATCGAGGAGAGGCTTAAGACCTACGCCCATCTGTTCGACGATAAAGTGATGAAGCAACTTAAACGGAGAAGATATACCGGATGGGGACGCCTTAGCCGCAAACTCATCAACGGAATCCGGGACAAACAGAGCGGAAAGACCATTCTTGATTTCCTTAAGAGCGACGGATTCGCTAATCGCAACTTCATGCAACTTATCCATGATGATTCCCTGACCTTTAAGGAGGACATCCAGAAGGCCCAAGTGTCTGGACAAGGTGACTCACTGCACGAGCATATCGCAAATCTGGCTGGTTCACCCGCTATTAAGAAGGGTATTCTCCAGACCGTGAAAGTCGTGGACGAGCTGGTCAAGGTGATGGGTCGCCATAAACCAGAGAACATTGTCATCGAGATGGCCAGGGAAAACCAGACTACCCAGAAGGGACAGAAGAACAGCAGGGAGCGGATGAAAAGAATTGAGGAAGGGATTAAGGAGCTCGGGTCACAGATCCTTAAAGAGCACCCGGTGGAAAACACCCAGCTTCAGAATGAGAAGCTCTATCTGTACTACCTTCAAAATGGACGCGATATGTATGTGGACCAAGAGCTTGATATCAACAGGCTCTCAGACTACGACGTGGACGCCATCGTCCCTCAGAGCTTCCTCAAAGACGACTCAATTGACAATAAGGTGCTGACTCGCTCAGACAAGAACCGGGGAAAGTCAGATAACGTGCCCTCAGAGGAAGTCGTGAAAAAGATGAAGAACTATTGGCGCCAGCTTCTGAACGCAAAGCTGATCACTCAGCGGAAGTTCGACAATCTCACTAAGGCTGAGAGGGGCGGACTGAGCGAACTGGACAAAGCAGGATTCATTAAACGGCAACTTGTGGAGACTCGGCAGATTACTAAACATGTCGCCCAAATCCTTGACTCACGCATGAATACCAAGTACGACGAAAACGACAAACTTATCCGCGAGGTGAAGGTGATTACCCTGAAGTCCAAGCTGGTCAGCGATTTCAGAAAGGACTTTCAATTCTACAAAGTGCGGGAGATCAATAACTATCATCATGCTCATGACGCATATCTGAATGCCGTGGTGGGAACCGCCCTGATCAAGAAGTACCCAAAGCTGGAAAGCGAGTTCGTGTACGGAGACTACAAGGTCTACGACGTGCGCAAGATGATTGCCAAATCTGAGCAGGAGATCGGAAAGGCCACCGCAAAGTACTTCTTCTACAGCAACATCATGAATTTCTTCAAGACCGAAATCACCCTTGCAAACGGTGAGATCCGGAAGAGGCCGCTCATCGAGACTAATGGGGAGACTGGCGAAATCGTGTGGGACAAGGGCAGAGATTTCGCTACCGTGCGCAAAGTGCTTTCTATGCCTCAAGTGAACATCGTGAAGAAAACCGAGGTGCAAACCGGAGGCTTTTCTAAGGAATCAATCCTCCCCAAGCGCAACTCCGACAAGCTCATTGCAAGGAAGAAGGATTGGGACCCTAAGAAGTACGGCGGATTCGATTCACCAACTGTGGCTTATTCTGTCCTGGTCGTGGCTAAGGTGGAAAAAGGAAAGTCTAAGAAGCTCAAGAGCGTGAAGGAACTGCTGGGTATCACCATTATGGAGCGCAGCTCCTTCGAGAAGAACCCAATTGACTTTCTCGAAGCCAAAGGTTACAAGGAAGTCAAGAAGGACCTTATCATCAAGCTCCCAAAGTATAGCCTGTTCGAACTGGAGAATGGGCGGAAGCGGATGCTCGCCTCCGCTGGCGAACTTCAGAAGGGTAATGAGCTGGCTCTCCCCTCCAAGTACGTGAATTTCCTCTACCTTGCAAGCCATTACGAGAAGCTGAAGGGGAGCCCCGAGGACAACGAGCAAAAGCAACTGTTTGTGGAGCAGCATAAGCATTATCTGGACGAGATCATTGAGCAGATTTCCGAGTTTTCTAAACGCGTCATTCTCGCTGATGCCAACCTCGATAAAGTCCTTAGCGCATACAATAAGCACAGAGACAAACCAATTCGGGAGCAGGCTGAGAATATCATCCACCTGTTCACCCTCACCAATCTTGGTGCCCCTGCCGCATTCAAGTACTTCGACACCACCATCGACCGGAAACGCTATACCTCCACCAAAGAAGTGCTGGACGCCACCCTCATCCACCAGAGCATCACCGGACTTTACGAAACTCGGATTGACCTCTCACAGCTCGGAGGGGATGAGGGAGCTGATGATCCAAAAAAGAAGAGAAAGGTAGATCCAAAAAAGAAGAGAAAGGTAGATCCAAAAAAGAAGAGAAAGGTAGGGCCcGGTTCCGGTTCCGGTTCCGGTTCCGGTTCCCAACTCGTGAAGAGTGAACTTGAGGAGAAAAAGTCGGAGCTGCGGCACAAATTGAAATACGTACCGCATGAATACATCGAACTTATCGAAATTGCTAGGAACTCGACTCAAGACAGAATCCTTGAGATGAAGGTAATGGAGTTCTTTATGAAGGTTTATGGATACCGAGGGAAGCATCTCGGTGGATCACGAAAACCCGACGGAGCAATCTATACGGTGGGGAGCCCGATTGATTACGGAGTGATCGTCGACACGAAAGCCTACAGCGGTGGGTACAATCTTCCCATCGGGCAGGCAGATGAGATGCAACGTTATGTCGAAGAAAATCAGACCAGGAACAAACACATCAATCCAAATGAGTGGTGGAAAGTGTATCCTTCATCAGTGACCGAGTTTAAGTTTTTGTTTGTCTCTGGGCATTTCAAAGGCAACTATAAGGCCCAGCTCACACGGTTGAATCACATTACGAACTGCAATGGTGCGGTTTTGTCCGTAGAGGAACTGCTCATTGGTGGAGAAATGATCAAAGCGGGAACTCTGACACTGGAAGAAGTCAGACGCAAGTTTAACAATGGCGAGATCAATTTCCGCTCATAA

- 1. Amino acid sequence

MDKKYSIGLAIGTNSVGWAVITDEYKVPSKKFKVLGNTDRHSIKKNLIGALLFDSGETAEATRLKRTARRRYTRRKNRICYLQEIFSNEMAKVDDSFFHRLEESFLVEEDKKHERHPIFGNIVDEVAYHEKYPTIYHLRKKLVDSTDKADLRLIYLALAHMIKFRGHFLIEGDLNPDNSDVDKLFIQLVQTYNQLFEENPINASGVDAKAILSARLSKSRRLENLIAQLPGEKKNGLFGNLIALSLGLTPNFKSNFDLAEDAKLQLSKDTYDDDLDNLLAQIGDQYADLFLAAKNLSDAILLSDILRVNTEITKAPLSASMIKRYDEHHQDLTLLKALVRQQLPEKYKEIFFDQSKNGYAGYIDGGASQEEFYKFIKPILEKMDGTEELLVKLNREDLLRKQRTFDNGSIPHQIHLGELHAILRRQEDFYPFLKDNREKIEKILTFRIPYYVGPLARGNSRFAWMTRKSEETITPWNFEEVVDKGASAQSFIERMTNFDKNLPNEKVLPKHSLLYEYFTVYNELTKVKYVTEGMRKPAFLSGEQKKAIVDLLFKTNRKVTVKQLKEDYFKKIECFDSVEISGVEDRFNASLGTYHDLLKIIKDKDFLDNEENEDILEDIVLTLTLFEDREMIEERLKTYAHLFDDKVMKQLKRRRYTGWGRLSRKLINGIRDKQSGKTILDFLKSDGFANRNFMQLIHDDSLTFKEDIQKAQVSGQGDSLHEHIANLAGSPAIKKGILQTVKVVDELVKVMGRHKPENIVIEMARENQTTQKGQKNSRERMKRIEEGIKELGSQILKEHPVENTQLQNEKLYLYYLQNGRDMYVDQELDINRLSDYDVDAIVPQSFLKDDSIDNKVLTRSDKNRGKSDNVPSEEVVKKMKNYWRQLLNAKLITQRKFDNLTKAERGGLSELDKAGFIKRQLVETRQITKHVAQILDSRMNTKYDENDKLIREVKVITLKSKLVSDFRKDFQFYKVREINNYHHAHDAYLNAVVGTALIKKYPKLESEFVYGDYKVYDVRKMIAKSEQEIGKATAKYFFYSNIMNFFKTEITLANGEIRKRPLIETNGETGEIVWDKGRDFATVRKVLSMPQVNIVKKTEVQTGGFSKESILPKRNSDKLIARKKDWDPKKYGGFDSPTVAYSVLVVAKVEKGKSKKLKSVKELLGITIMERSSFEKNPIDFLEAKGYKEVKKDLIIKLPKYSLFELENGRKRMLASAGELQKGNELALPSKYVNFLYLASHYEKLKGSPEDNEQKQLFVEQHKHYLDEIIEQISEFSKRVILADANLDKVLSAYNKHRDKPIREQAENIIHLFTLTNLGAPAAFKYFDTTIDRKRYTSTKEVLDATLIHQSITGLYETRIDLSQLGGDEGADDPKKKRKVDPKKKRKVDPKKKRKVGPGSGSGSGSGSQLVKSELEEKKSELRHKLKYVPHEYIELIEIARNSTQDRILEMKVMEFFMKVYGYRGKHLGGSRKPDGAIYTVGSPIDYGVIVDTKAYSGGYNLPIGQADEMQRYVEENQTRNKHINPNEWWKVYPSSVTEFKFLFVSGHFKGNYKAQLTRLNHITNCNGAVLSVEELLIGGEMIKAGTLTLEEVRRKFNNGEINFRS*


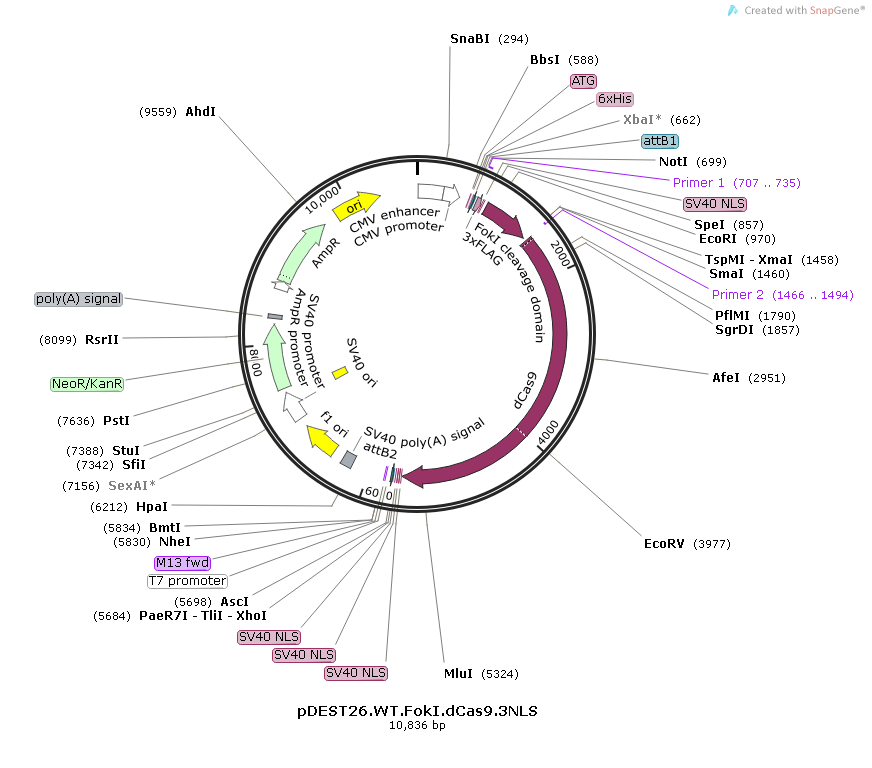


Map of the human plasmid carrying WT.FOK.dCas9


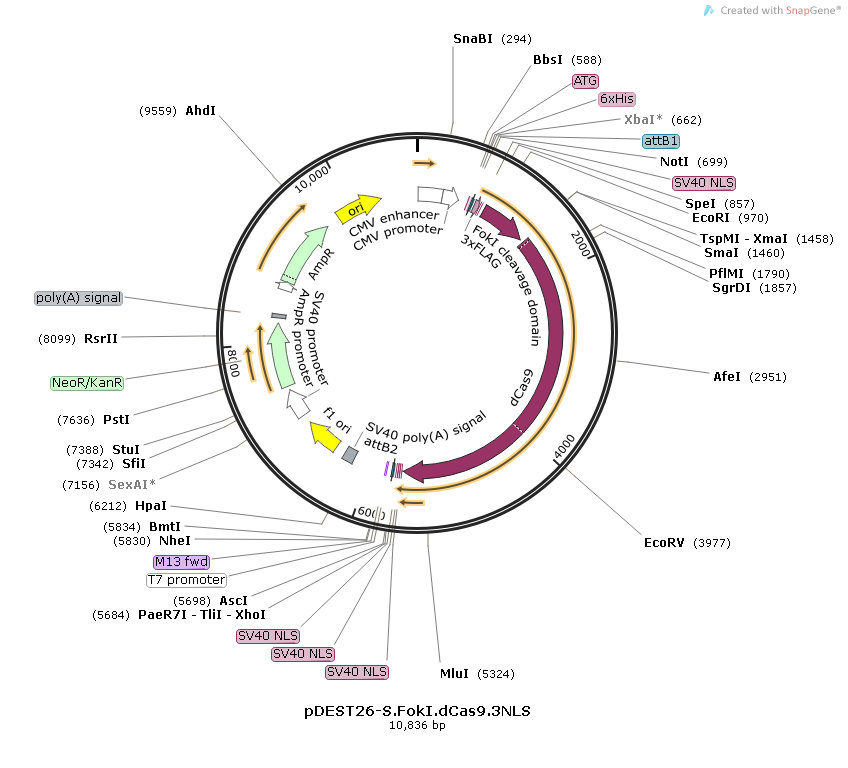


Map of the human plasmid carrying Sharky.FOK.dCas9


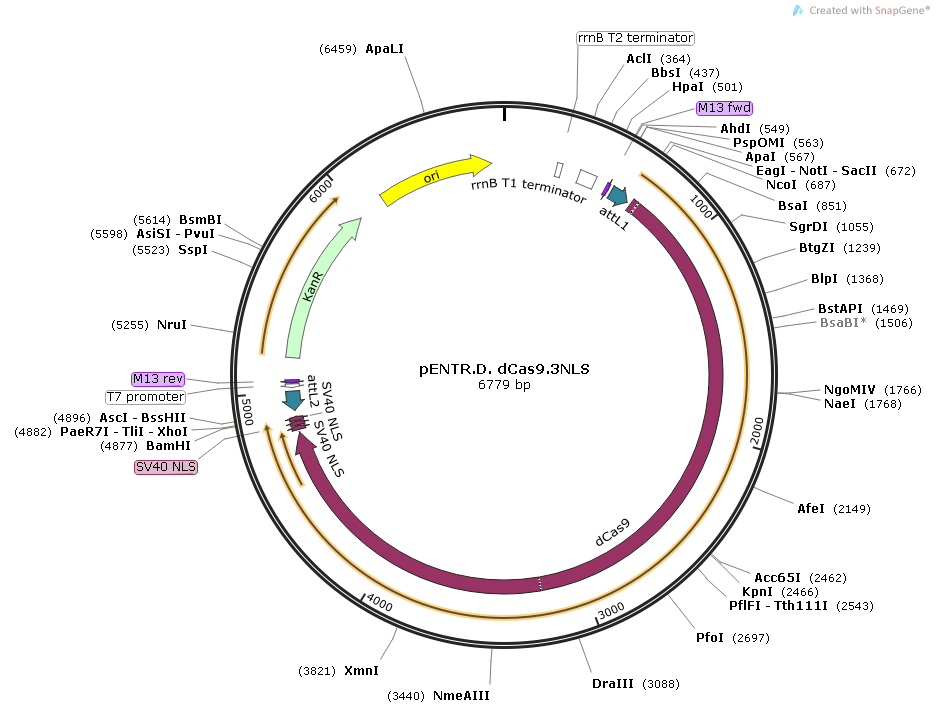


pENTRD.Topo.dCas9 Map


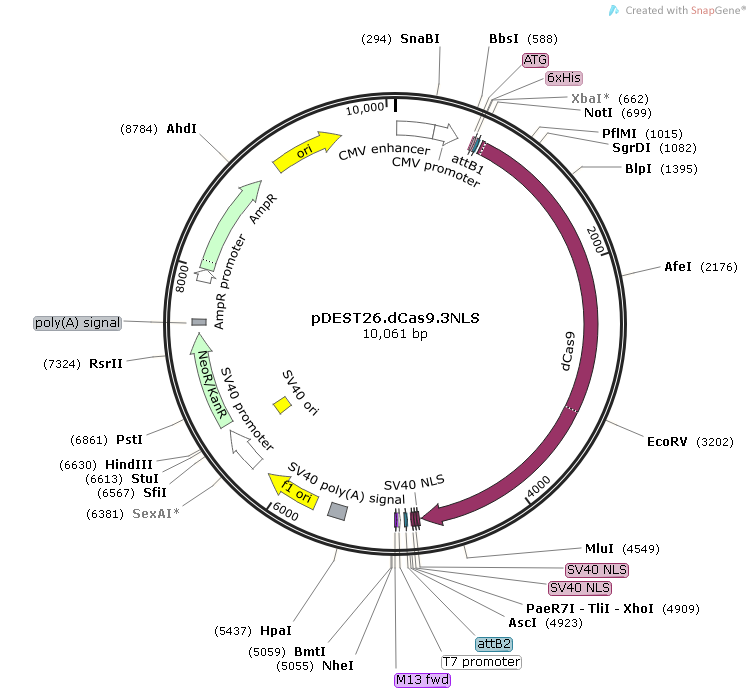


pDEST26.dCas9 Map
